# Characterizing chemical signaling between engineered “microbial sentinels” in porous microplates

**DOI:** 10.15252/msb.202110785

**Published:** 2022-03-22

**Authors:** Christopher A Vaiana, Hyungseok Kim, Jonathan Cottet, Keiko Oai, Zhifei Ge, Kameron Conforti, Andrew M King, Adam J Meyer, Haorong Chen, Christopher A Voigt, Cullen R Buie

**Affiliations:** ^1^ Department of Mechanical Engineering Massachusetts Institute of Technology Cambridge MA USA; ^2^ Synthetic Biology Center Department of Biological Engineering Massachusetts Institute of Technology Cambridge MA USA

**Keywords:** hydrogels, living materials, microbial communication, quorum signaling, synthetic biology, Biotechnology & Synthetic Biology, Signal Transduction

## Abstract

Living materials combine a material scaffold, that is often porous, with engineered cells that perform sensing, computing, and biosynthetic tasks. Designing such systems is difficult because little is known regarding signaling transport parameters in the material. Here, the development of a porous microplate is presented. Hydrogel barriers between wells have a porosity of 60% and a tortuosity factor of 1.6, allowing molecular diffusion between wells. The permeability of dyes, antibiotics, inducers, and quorum signals between wells were characterized. A “sentinel” strain was constructed by introducing orthogonal sensors into the genome of *Escherichia coli* MG1655 for IPTG, anhydrotetracycline, L‐arabinose, and four quorum signals. The strain’s response to inducer diffusion through the wells was quantified up to 14 mm, and quorum and antibacterial signaling were measured over 16 h. Signaling distance is dictated by hydrogel adsorption, quantified using a linear finite element model that yields adsorption coefficients from 0 to 0.1 mol m^−3^. Parameters derived herein will aid the design of living materials for pathogen remediation, computation, and self‐organizing biofilms.

## Graphical abstract

Microbes interact by chemical diffusion through materials, a concept that inspires “engineered living materials”. To parameterize signaling through materials, a porous culture microplate is fabricated to measure chemical signaling between isolated wells of engineered *E*. *coli*.

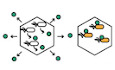

## Introduction

Living materials are a promising tool to be applied in such diverse applications as therapeutics (Duraj‐Thatte *et al*, 2019), construction (Heveran *et al*, 2020), fashion (Bader *et al*, 2016), and extraterrestrial exploration (Menezes *et al*, 2015). They consist of cells embedded in a scaffold, such as textiles (Yao *et al*, 2015; Moser *et al*, 2019; Nguyen *et al*, 2021), 3D‐printed substrates (Connell *et al*, 2013; Axpe & Oyen, 2016; Schaffner *et al*, 2017; Liu *et al*, 2018; Correia Carreira *et al*, 2020; Smith *et al*, 2020), or cement (Wiktor & Jonkers, 2011; Reddy *et al*, 2015; Başaran Bundur *et al*, 2017). It is challenging to design cells that function reliably in these environmental contexts. For example, bacteria serving as chemical sensors are typically characterized in liquid culture, but the diffusion of the chemical and the state of the cell will be different than in a more complex matrix (Masiello *et al*, 2013; Bereza‐Malcolm *et al*, 2015; Del Valle *et al*, 2020). In particular, bacteria consume and produce molecules, including metabolites and cell‐cell communication (quorum) signals. The interaction of these molecules with the materials and their transport properties impact how a cell will perform, but it is difficult to parameterize these properties to inform the design process.

Materials differ in their molecular transport properties. Porous hydrogel scaffolds that have been investigated for living materials include agarose (Gerber *et al*, 2012; Gonzalez *et al*, 2020), alginate (Kim *et al*, 2011), acrylamide (Lin *et al*, 2016; Liu *et al*, 2017, 2018; Sankaran *et al*, 2019), dextran (Guo *et al*, 2020), methacrylates (Liu *et al*, 2018), and bacterial cellulose (Gilbert & Ellis, 2019; Birnbaum *et al*, 2020). Cross‐linked agarose has pores that range from 0.5 to 500 µm depending on the fabrication processes, with porosity (the ratio of void volume to total volume of a material) of about 30% (Annabi *et al*, 2010; Rodriguez Corral *et al*, 2020). Dextran hydrogel pores are 10–100 µm and have been covalently functionalized with antibiotic‐producing *Escherichia Coli* (*E. coli*); the large pore size provides free diffusion of Isopropyl β‐d‐1‐thiogalactopyranoside (IPTG) and arabinose (ara) inducers and allows the delivery of the antibiotic lysostaphin (Guo *et al*, 2020). Pores of this size allow cell migration as well, unless the microbes are actively tethered to the matrix. Methacrylate hydrogels derived from Pluronic® F127 were used to print a spatially compartmentalized biosynthetic consortia; the smaller pore size of methacrylates can be less than 10 µm and can physically trap cells (Liu *et al*, 2018; Saha *et al*, 2018). Using a methacrylate “bio‐ink”, a spatially segregated co‐culture of *E. coli* and *Saccharomyces cerevisiae* was 3D‐printed; the strains worked together to produce betaxanthins (a commercially valuable food colorant) (Johnston *et al*, 2020). Entrapment of cells in the ink enables their lyophilization, storage, and long‐term use, while segregation of species improves their coexistence.

The diffusion of chemicals through a material can be effectively modeled as its “tortuosity”—a measure of the twists and turns a solute makes during its “zig‐zag” movement through the material. The tortuosity has been experimentally measured for a number of solutes, with variations in the tortuosity factor ranging from 0.5 to 4.0 (Zhang & Bishop, 1994; Melo, 2005). The diffusion coefficients in water *D* *_aq_* of common signaling molecules range from 5 to 50 x 10^‐6^ cm^2^ s^‐1^ (Stewart, 1998, 2003). The environment through which these molecules travel dictates how far the effective diffusion *D* *_e_* deviates from diffusion through water. It has been suggested to use a mean *D* *_e_* */D* *_aq_* of 0.25 for organic solutes in biofilms (Stewart, 2003), although the exact value is predicted to fluctuate by approximately 50% as the biofilm porosity changes (Zhang & Bishop, 1994).

Cells can perform sensing and computing functions within a living material that are otherwise difficult operations to encode in the inorganic scaffold. Genetic sensors respond to an environmental signal and control the activity of a promoter, and circuits integrate the information from sensors or enact a dynamic response. Sensors have been designed to operate in a living material by mathematically considering the impact of signaling molecule transport on the dynamics of activating the sensor. For example, the effective diffusion of small molecules through acrylamide is estimated to be 1.5 × 10^−10^ m^2^ s^−1^ (Lin *et al*, 2016; Liu *et al*, 2018). This was mathematically combined with the response of the promoter to predict the activation by the small molecules IPTG and N‐hexanoyl‐L‐homoserine lactone (OC6‐HSL, or OC6) as they diffused through the hydrogel (Liu *et al*, 2017).

Bacteria communicate by exchanging small molecules, referred to as quorum signals (Waters & Bassler, 2005). These systems are commonly used to program cell‐cell communication, where a sender cell produces the molecule and the receiver cell has sensor that responds to it (Balagadde *et al*, 2008; Bischofs *et al*, 2009; Wu *et al*, 2014; Perry *et al*, 2016; Scott & Hasty, 2016; preprint: Doong *et al*, 2017; He *et al*, 2017; Scott *et al*, 2017; Halleran & Murray, 2018; Kylilis *et al*, 2018; Leaman *et al*, 2018; preprint: Parkin & Murray, 2018; Alnahhas *et al*, 2020; preprint: Fedorec *et al*, 2020; preprint: Karkaria *et al*, 2020; Miano *et al*, 2020; Stephens & Bentley, 2020). These have been used to create many multicellular patterns in the field and could be used to differentiate cells within a living material; however, their use within complex matrixes poses a challenge (Dilanji *et al*, 2012; Borek *et al*, 2016; Gao *et al*, 2016a; Grant *et al*, 2016). LuxR‐family regulators respond to acyl‐homoserine lactones (HSLs), which are very small and can adsorb to materials (Kaeberlein *et al*, 2002; Bollmann *et al*, 2007; Gao *et al*, 2016b; Dade‐Robertson *et al*, 2018; Mukherjee & Bassler, 2019). Between colonies, the effective distance of quorum signaling through agar or *in situ* is over 4 cm after 10 h (Dilanji *et al*, 2012; preprint: Doong *et al*, 2017). Material adsorption at a boundary can produce a 100‐fold lower effector concentration at the receiving cell (Trovato *et al*, 2014).

Interactive co‐culture platforms have been instrumental in understanding how molecular diffusion plays a role in collective microbial function. Bulk liquid co‐cultures suffer from limited throughput and one strain can overtake the other in growth, while on agar colonies do not interact until reaching late stationary growth (Zhang & Wang, 2016; Fortuin, 2020). Membrane‐based co‐cultures allow chemicals to diffuse between strains. For example, the isolation chip—a culture chamber sandwiched between membranes with 30 nm pores—has increased the cultivability of soil microbes by allowing microbes to exchange chemicals with their native environment (Kaeberlein *et al*, 2002; Nichols *et al*, 2010; Berdy *et al*, 2017). This has been used to induce antibiotic production from otherwise silent gene clusters (Ling *et al*, 2015; Shi *et al*, 2017; Lodhi *et al*, 2018). The throughput can be massively improved using microfluidics, but these can be difficult to use, requiring specialized pumps and channels (Rogers *et al*, 2021).

In this manuscript, we describe a “porous microplate” consisting of spatially isolated culture wells separated by porous methacrylate barriers (Ge *et al*, 2016; Kim *et al*, 2021). The methacrylate we choose, hydroxyethyl methacrylate‐*co*‐ethylene glycol dimethacrylate (HEMA‐EDMA), has amorphous pores of 10–100 nm in size and a porosity of 60 percent. This culturing system allows the exchange of small molecules, but not cells, between wells. Unlike membrane‐based co‐cultures, the geometry of our plate has each well surrounded by six wells, each of which could isolate a separate strain. It is also easy to fabricate and pipette into the wells and does not require specialized equipment, in contrast to microfluidic approaches. To evaluate this system, we constructed a strain of *E. coli* that contains genetically‐encoded sensors for IPTG, anhydrotetracycline (aTc), and ara as well as four orthogonal HSLs. Using our system, we measured the effective diffusion through the HEMA‐EDMA barrier as well as an adsorption coefficient and tortuosity. These data parameterize a quantitative model of diffusion through a porous medium. In addition, we use the system to parameterize the diffusion of antibiotics. This work provides a method to separate strains as a means to study interactions between species making up a microbiota or to parameter models to aid the design of cells that functionalize living materials.

## Results

### Microplate fabrication and characterization

**Figure 1:**
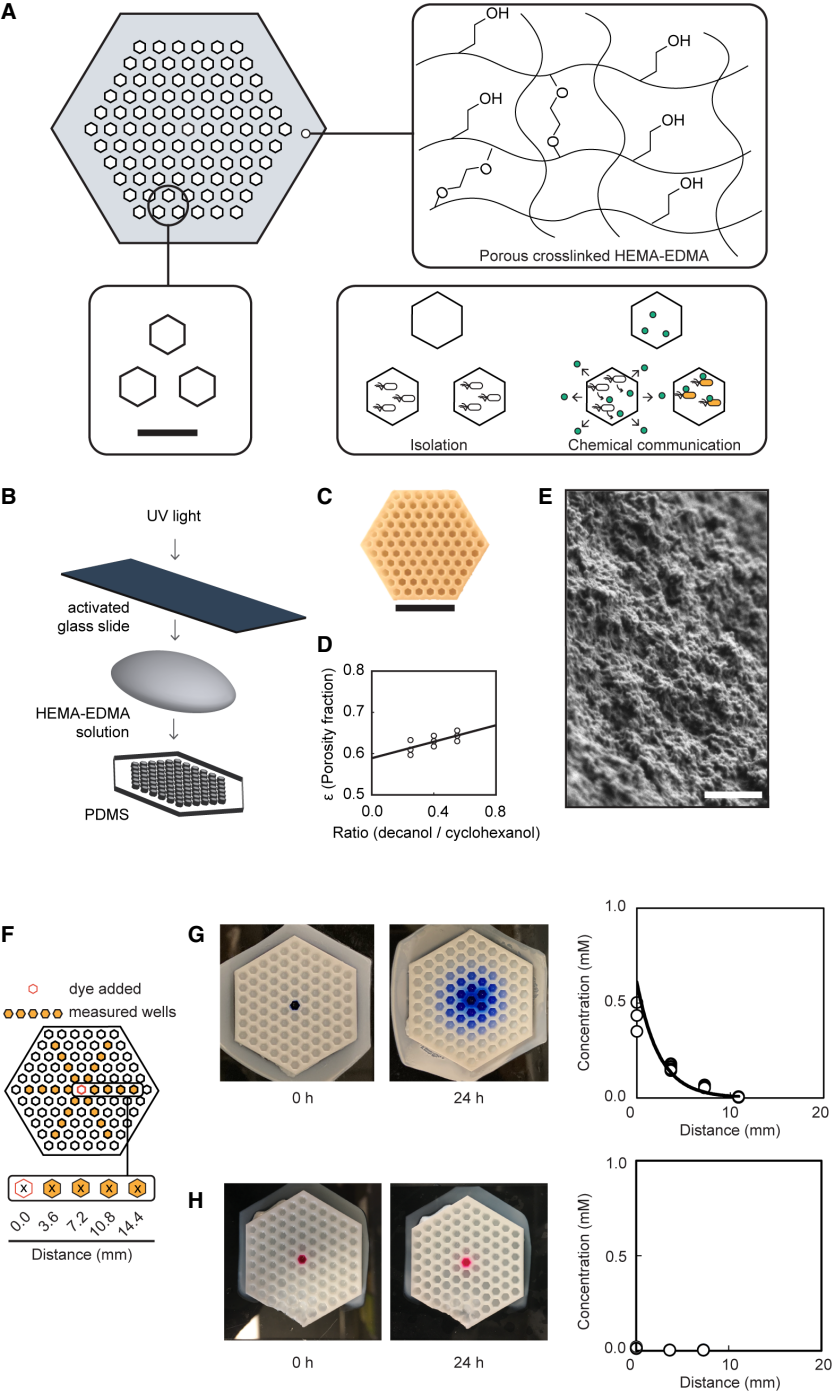
A porous microplate for chemically interactive cell culture ATwo‐dimensional graphical depiction of a hexagonal porous microplate is shown. The plate is constructed from cross‐linked hydroxyethyl methacrylate‐co‐ethylene glycol dimethacrylate (HEMA‐EDMA, large filled hexagon). Liquid culture wells hold a volume of 10.0 µl (small open hexagons). The lower left inset depicts well dimensions. Scale bar = 3.6 mm. The lower right inset describes the utility of the plate to isolate culture wells and allow chemical communication between cultures.BSchematic depiction of the microplate fabrication process.CPhotograph of a porous microplate; scale bar = 20.0 mm.DThe experimentally determined porosity *ε* is plotted as a function of the ratio of 1‐decanol to cyclohexanol used during fabrication. Three devices for each condition were fabricated and measured. Each data point represents a single measured device. All collect data points are plotted. A linear equation was fit to the data; *y* = 0.1*x* + 0.5887, *R* ^2^ = 0.5 based on a regression analysis.EScanning electron micrograph of cross‐linked HEMA‐EMDA; scale bar = 1.0 µm.FSchematic depiction of dye diffusion measurements is shown.G, HDiffusion of cotton blue (G) and rhodamine B (H) through the microplate. After 24 h, the concentration of dye in a selection of wells was measured and plotted as a function of distance from the center well. The experiment was repeated on different days, and all collected data are reported (open circles, *n* = 3 total data points for the center well, and *n* = 6 data points for the remainder wells). An exponential equation was fit to the average cotton blue data; *y* = 0.3 e^−0.29^ *^x^*, *R* ^2^ = 0.96).

The dimensions of the porous microplate were designed with a hexagonal layout to maximize neighboring well interactions and to facilitate manual cell recovery (Fig 1A, Appendix Fig S1). A hexagonal layout allows each well to have six immediate neighbors. The well centers were spaced out 3.6 mm apart horizontally which matches the spacing of a conventional 364‐well plate. The well width of 2.2 mm is large enough to fit a standard 20‐µl pipette tip for manual loading. In addition to the hexagonal well layout, alternative microplate designs can be fabricated that conform to standard commercial microplate dimensions. These include 96‐well, 384‐well, and 1536‐well formats (Appendix Fig S2). A range of feature sizes can be made, with a minimum of 0.56 mm observed in this work (the width of the walls between the wells in the 1536‐well dimensioned microplate (Appendix Fig S2C)).

The co‐polymer HEMA‐EDMA was chosen due to its biocompatibility and tunable porosity. The ratio of the two precursor monomers dictates the porosity of the final product. In this work, a 24 percentage by weight (wt %) of HEMA and 16 wt % of EDMA were used. The solvents 1‐decanol and cyclohexanol were added to the hydrogel precursor to act as “porogens” as the methacrylates cross‐linked around them. Different solvent choices and combinations can tune the porosity and pore size of the final material (Geyer *et al*, 2011; Ge *et al*, 2016). To fabricate the microplates, conventional soft lithography techniques were used. An acrylic mold was first laser cut to the proper dimensions and used to generate a PDMS master. The PDMS master then shapes the hydrogel, and the molded hydrogel precursor was cross‐linked with ultraviolet light to bond to a methacrylate‐functionalized glass slide (Fig 1B and C). Several methanol washes were employed to remove excess hydrogel precursors before the microplate was incubated in an appropriate buffer or culture medium for further use.

To explore the effect of solvent ratio on porosity, the 1‐decanol‐to‐cyclohexanol ratio was adjusted between 0.25 and 0.60, and an upward linear trend was observed between increasing solvent ratio and porosity (Fig 1D). As the ratio of 1‐decanol to cyclohexanol increases beyond 0.60, the integrity of the device suffers. Therefore, a ratio of 0.25 was used throughout the remainder of the work. The pores of the final cross‐linked material were examined using scanning electron microscopy. The amorphous porous mesh appears to have a distribution of pores of less than 1 µm in diameter, which is smaller than *E. coli* cells, and is consistent with previous measurements (Fig 1E, Appendix Fig S3) (Ge *et al*, 2016).

To test the permeability of the microplate walls, we measured the effective diffusion of the visible dyes cotton blue and rhodamine B from the center well (Fig 1F). These dyes were selected because of their comparable molecular weight, but different water solubilities. To the center well of separate microplates was added an aqueous solution of dye, and to the remaining wells was added Milli‐Q water. Each device was incubated at 30°C for 24 h before being assayed for the presence of the dyes. After incubation, cotton blue was present in the outer wells and its concentration as function of distance from the center exponentially decays (Fig 1G). In contrast, rhodamine B was not detected in any outer wells, and the center well was nearly depleted of the starting dye solution (Fig 1H). While rhodamine was not detected in solution in the well, the hydrogel surface was visibly stained with the dye, suggesting a strong attraction of rhodamine to the polymer. Our observations suggest that interactions between the solute and the HEMA‐EDMA microplate walls are molecule‐dependent and that rhodamine B was adsorbed by the polymer.

### Construction and evaluation of a *E. coli* sensor strain

**Figure 2:**
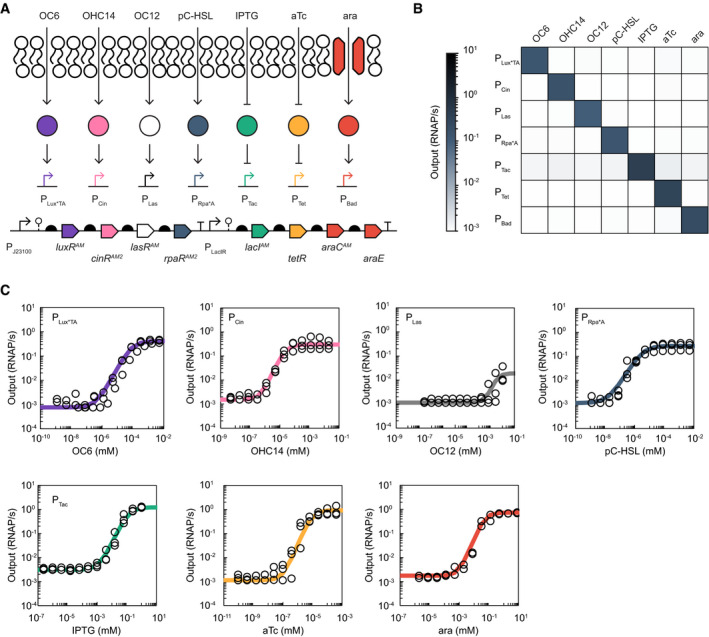
The sentinel strain responds orthogonally to small molecule inducers and quorum signals The genetic and molecule schematic depictions of the Marionette‐Q cluster are shown.The chemical cross‐reactivity heatmap of the 7 output promoters with each inducer is shown. The mean promoter activity of three replicates done on different days is plotted. Individual data points are plotted in Appendix Fig S8.The resulting dose–response curves of the seven sensors and output promoters in response to their cognate inducer: 3‐oxohexanoyl‐homoserine lactone (OC6) with P_Lux*TA_, 3‐hydroxytetradecanoyl‐homoserine lactone (OHC14) with P_Cin_, N‐3‐oxododecanoyl‐homoserine lactone (OC12) with P_Las_, para‐coumaroyl‐homoserine lactone (pC‐HSL) with P_Rpa*A_, isopropyl β‐D‐1‐thiogalactopyranoside (IPTG) with P_Tac_, L‐arabinose (ara) with P_Bad_, and anhydrotetracycline (aTc) with P_Tet_. The experiment was repeated on different days, and all collected data are reported (*n* = at least 3). Response profiles are each fit to a Hill equation using Excel (line; see Table 1 for coefficients). Data (open circles) are collected as median fluorescent signal of at least 20,000 cells and converted to RNAP/s units (see Materials and Methods).

Bacteria can provide a sensing function to a living material. To evaluate a sensor strain in our device, we engineered *E. coli* to contain an array of small molecule sensors. Four LuxR‐family acyl‐homoserine lactone activators were included: *luxR*, *cinR*, *lasR*, and *rpaR*. Specific mutations were made to the sensors, as well as libraries of directed evolution, to reduce cross‐reactivity and improve performance (Appendix Tables S1 and S2, Appendix Fig S4A). Genes for the four mutated quorum sensors were combined with those of three previously optimized sensors tetRAM, lacIAM, and araCA from *E. coli* Marionette, as well as an araE transporter (Meyer *et al*, 2019), to create a seven‐sensor array integrated into landing pads previously placed in the *E. coli* MG1655 genome (Fig 2A, Appendix Fig S5). Receiver strains that respond to each small molecule were built by transforming the strain carrying the array with a p15a plasmid that contains a sensor output promoter driving the expression of YFP (Appendix Fig S6A). The sensors were optimized to increase the dynamic range and tested for cross reactivity (Fig 2B, Appendix Figs S6B and S7). The dose response curves for the final sensors are shown in Fig 2C and parameters from a fit to a response function are provided in Table 1.

**Table 1:** Output promoter response parameters.

Output promoter	*y*_min_ (RNAP/s)	*y*_max_ (RNAP/s)	*K*	*n*
P_Lux*TA_	0.0008	0.4	9.4 × 10^−5^	1.13
P_Cin_	0.0015	0.3	2.9 × 10^−5^	1.46
P_Las_	0.0011	0.02	1.5 × 10^−2^	1.08
P_Rpa*A_	0.0011	0.3	6.6 × 10^−6^	0.98
P_Tac_	0.0030	1.2	1.4 × 10^0^	1.35
P_Tet_	0.0011	0.9	9.0 × 10^−6^	1.50
P_Bad_	0.0017	0.7	1.5 × 10^0^	1.49

### Characterizing the transport of inducers through the microplate

Transport in the device is influenced by diffusion and adsorption. As diffusion of molecules in porous media depends on the structure of the porous material and the phases involved, we considered the effective diffusivity *D_e_* to be that of a saturated porous media:

(1) $$D_{e}=D_{\mathrm{aq}}\varepsilon \tau^{‐1}$$

where *D_aq_* is the diffusion coefficient in water (m^2^ s^−1^), *ε* is the porosity, and *τ* is the tortuosity factor. The tortuosity factor accounts for the reduced diffusivity due to solid grains of the hydrogel that impede Brownian motion.

The adsorption of the molecule to the surface of the porous matrix can be accounted for by different adsorption isotherm models. In our case, a linear model (Henry’s adsorption isotherm) was used,

(2) $$Q_{e}=K_{H}C_{e}$$

where $Q_{e}$ is the mass adsorbed (mol kg^−1^), $C_{e}$ is the adsorbate concentration at equilibrium (mol m^−3^), and K_H_ (m^3^ kg^−1^) is the partition coefficient. We used published values for *D_aq_* of glycine (1.1 × 10^−9^ m^2^ s^−1^) and *D_e_* of glycine through HEMA‐EDMA (3.47 × 10^−10^ m^2^ s^−1^) (Stewart, 2003; Ge *et al*, 2016), and our experimentally determined porosity fraction *ε* = 0.6 to calculate the tortuosity *τ* = 1.9. Diffusion was modeled in the wells using *D_aq_* of glycine (1.1 × 10^−9^ m^2^ s^−1^).

**Figure 3:**
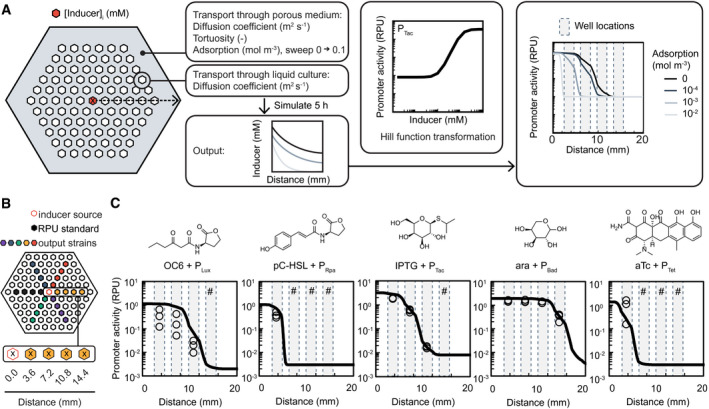
Simulation and experimental measurement of inducer diffusion through the porous microplate Flow chart representing the simulation of inducer transport through the microplate using a linear finite element model of effective diffusion through a porous medium. Inducer concentration in the center well was set to an initial value [inducer]_i_ at time *t* = 0 h filled red hexagon: (20.0 mM IPTG, 0.0004 mM aTc, 160.0 mM L‐arabinose, 0.1 mM OHC6‐HSL, 0.1 mM pC‐HSL, or 0.4 mM OHC12‐HSL), and constants were defined for both the polymer (large filled hexagon) and the liquid culture wells (small open hexagons). The resulting cross‐sectional concentration profile (dashed arrow) after 5‐h simulated diffusion time was collected as inducer concentration (mM) as a function of distance from the center of the center well (“x”). The resulting concentration values were converted to promoter activity (RNAP/s) using the Hill coefficients for each inducer with output strain (Table 1). The resulting output is a plot of the cross‐sectional simulated promoter activity for each inducer with promoter pair as a function of distance from the center well. The gray regions between dotted vertical lines represent the location of wells (not including the center) from which measurements were made. Simulations were run at a range of different adsorption coefficient values using the COMSOL software.Schematic depiction of the inducer diffusion experimental setup. The distances between the center of the center well containing the inducers (red open hexagon) and the center of the wells containing output strains (filled hexagons) are indicated with an “x”. A cocktail of all 5 inducers tested was added, and the respective concentrations are described in A.The simulated and measured promoter activity of each inducer and output strain pair is plotted as a function of distance from the center well after 5‐h incubation. Inducer molecules are shown above each corresponding plot. Data points represent measurements taken from the well of one device (open circles) and represent the mean promoter activity value for a given well. The experiment was repeated on different days, and all collected data are reported (*n* = 3). # = no signal was detected at these wells. The lines represent the resulting simulation from part A for each inducer with output strain pair at the absorbance value that most closely fit the experimental data (see Table 2 for values).

Using these data, we simulated transport through the microplate of the seven inducers that activate the receiver strains (Fig 3A). The diffusion coefficients and tortuosity were held constant while the adsorption coefficient was swept from 0 to 0.1 mol m^−3^. The resulting cross‐sectional values for inducer concentration at 5 h were collected. The inducer concentration was then transformed with a Hill equation using the coefficients listed in Table 1. The result of the simulation was the predicted output of an inducer‐sensor pair as a function of distance from the center well.

The simulations were validated by seeding receiver strains and the RPU reference strain in a radial arrangement around a center well, with the remainder of the wells filled with media (Fig 3B). A concentrated inducer cocktail was added to the center well, and the microplate was incubated for 5 h. The concentrations in the cocktail were selected to be 20 times higher than the maximum concentration in the dose response curves to ensure a high enough concentration several wells away. After incubation, the promoter activity of each well was measured by cytometry (Appendix Fig S8) and overlaid with the simulation resulting from the best matching adsorption value (Fig 3C).

Each of the inducers tested produced a unique effective diffusion profile, due to a combination of the different molecular properties of each inducer and the different response functions of the cognate sensor. We were able to fit adsorption values to five of the seven inducer‐sensor pairs, while the remaining two did not diffuse through the walls at all, and therefore could not be fit with our model. Of the five diffusible inducers, we observed adsorption coefficients ranging from 0 mol m^−3^ (no adsorption) to > 0.1 mol m^−3^ (complete adsorption). The adsorption coefficients that best‐fit each inducer when compared to the experimental measurements through HEMA‐EDMA, and the respective correlation coefficients for their comparison, are listed in Table 2. Source data for the simulations are provided in the Appendix.

**Table 2:** Calculated adsorption and solubility parameters of inducers.

Abbreviation	K_H_ (mol m^−3^)	ClogP
OC6	0.002	−0.49
OHC14	> 0.1	3.70
OC12	> 0.1	2.69
pC‐HSL	0.01	0.30
IPTG	0.0005	−0.32
aTc	0.002	0.31
ara	0.0001	−2.18

A trend that emerges from these data is that the distance traveled by an inducer through the microplate is proportional to the water solubility of the molecule. To support this claim, the ClogP value of each inducer was calculated, and these data were plotted as a function of the distance that inducer was observed to activate the receiver strain (Appendix Fig S9). The ClogP value represents a partitioning coefficient between octanol and water, which can be computationally estimated; molecules with a larger ClogP value prefer octanol and therefore are less water soluble than molecules with a smaller ClogP value. Calculated ClogP values for each inducer molecule are listed in Table 2. The quorum molecules OHC14 and OC12 had the highest predicted ClogP values of 3.70 and 2.69, respectively, and neither diffused through the microplate at all. In contrast, ara has the lowest computed ClogP value of −2.18, the most water soluble of the inducers tested, and traveled the farthest distance. We surmise that the predominant factor determining the effective diffusion distance of each molecule is the propensity of the hydrogel matrix to adsorb that inducer, and this adsorption is related to the solubility of the molecule.

### Quorum communication through the microplate

**Figure 4:**
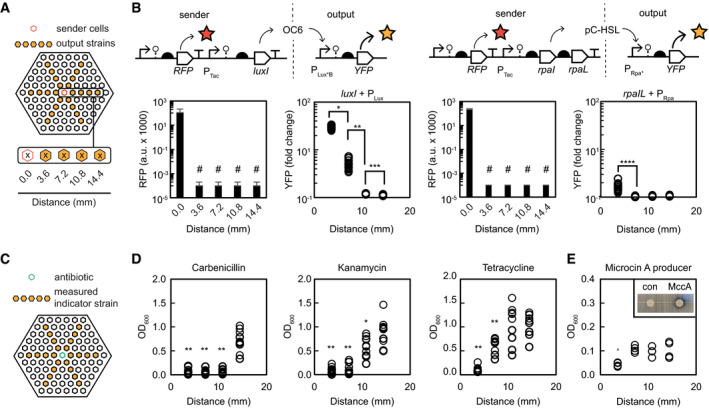
Quorum signaling a growth inhibition can be observed between culture wells Schematic depiction of quorum sender and receiver strains co‐cultured in the porous microplate.The genetic circuits encoding the quorum sender and receiver pairs are shown. A sender strain expresses RFP from a constitutive promoter and expresses a quorum synthase (*luxI* or *rpaIL*) from a P_Tac_ promoter. Quorum molecules (OC6 or pC‐HSL) diffuse through the hydrogel (dashed line) and control YFP expression in a receiver strain via P_Lux_ or P_Rpa_ promoters, respectively. The resulting fluorescent signals after co‐culture are plotted. The RFP signal (arbitrary units) is monitored to show that the sender strain signal is only present in the center well. # = No detectible signal. The YFP signal of the receiver strain is measured from the wells circled in A as a function of distance from the center well, and the signal is divided by that of the same strain cultured in the absence of inducer to obtain fold change. The experiment was repeated in triplicate on different days, and all collected data are reported (open circles). Data statement: RFP data are presented as the average of the median fluorescence of at least 20,000 cells (*n* = 3). YFP data are presented as the fold change of the median fluorescence of at least 20,000 cells (*n* = 18). *P = 3 × 10^−30^, **P = 1 × 10^−5^, ***P = 2.8 × 10^−6^, ****P = 6.5 × 10^−9^ (2‐tailed Student’s *t*‐test).Schematic depiction of a wild‐type *E. coli* indicator strain cultured with antibiotics added to the center well.The optical density (OD_600_) is measured in the wells indicated in C and plotted. The experiment was repeated on different days, and all collected data are reported (open circles, *n* = 9 total data points for each condition). **P < 0.001, *P < 0.1 (2‐tailed Student’s *t*‐test). Exact *P* values are in Appendix Fig S13.The experiment depicted in C was conducted with a strain producing the antibiotic Microcin A in the center well, and the results were plotted as described. All data points collected from a single microplate are plotted (open circles, *n* = 4). Inset: The activity of the Microcin A producer was validated by spotting the producing strain onto a lawn of indicator cells and observing a zone of clearing when compared to a spotted non‐producing strain. ^*P* = 0.2 (Student’s *t*‐test).

We next tested quorum signaling between wells using engineered sender and receiver strains. Our previous characterizations suggested that the quorum signals OC6 and pC‐HSL would be able to travel through the microplate walls, with OC6 reaching a greater distance. Quorum senders were engineered by placing a homoserine lactone (HSL) synthase under control of the inducible P_Tac_ promoter (Appendix Fig S10): *luxI* which produces OC6, or *rpaI* which produces pC‐HSL (along with the transporter protein *rpaL*). The plasmid also contained a red fluorescent protein (mRFP1) under control of a strong constitutive promoter in order to track the location of the sender strains. Sender strains were individually added to the center well of a microplate along with IPTG, while the cognate output strain was added in a radial pattern surrounding the center well (Fig 4A).

After overnight incubation, the wells were assayed for fluorescence signal by cytometry, and the data were plotted as a function of distance from the center well. Both the *luxI* and *rpaIL* sender strains were found to be trapped in the center well, as no RFP signal was detected in the surrounding wells (Fig 4B). Activation of the P_LuxB_ output strain was observed up to 7 mm away, with a 30‐fold activation in the nearest well (3.6 mm) and 8‐fold activation in the next well (7.2 mm). In contrast, activation of the P_Rpa*_ receiver strain was detectible with a less than 2‐fold activation only in the nearest well (3.6 mm from the center). The interaction of each quorum molecule with the hydrogel matrix likely determines the difference in signaling distance through the HEMA‐EDMA.

### Antibiotic diffusion through the microplate

We tested the transport of antibiotics between wells by measuring the cell death of a wild‐type *E. coli* indicator strain. The center well was loaded with a concentrated aqueous solution of a single antibiotic. The indicator strain was seeded in a radial pattern surrounding the center well and cultured overnight (Fig 4C). The next day, cell death was assayed by removing the cultures from the wells and measuring optical density. Each of the three antibiotics tested was able to diffuse through the walls of the microplate, and each induced cell death in a distance‐dependent manner. We observed a relative OD_600_ of less than 10% at a distance of three wells from the center well after culture with carbenicillin; two wells with kanamycin; and one well with tetracycline, suggesting these antibiotics inhibited greater than 90% of cell growth in these wells (Fig 4D, Appendix Fig S11). Additionally, kanamycin was observed to inhibit on average 50% of cell growth in the third well from the center (Fig 4D). These effective killing distances did not correlate with the ClogP values calculated for each antibiotic.

We finally observed the interaction between a microcin‐producing microbe and a wild‐type indicator strain through wells of the microplate. Microcins are a class of small antibacterial molecules, usually peptides, produced by bacteria that also express a cognate resistance gene (Piskunova *et al*, 2017). They are observed to help Gram‐negative bacteria displace neighboring cultures during biofilm development. Recently, the probiotic strain *E. coli* Nissle 1917 was demonstrated to use microcin diffusion to limit the spread of pathogenetic *Enterobacteriaceae* in the inflamed gut during dysbiosis. We built a strain of *E. coli BL21* to express the *mcc7* gene cluster responsible for biosynthesis of the ribosomally synthetized and post‐translationally modified peptide (RiPP) microcin C7 (McC7) and its cognate resistance transporter under the control of the inducible P_T7Lac_ promoter (Appendix Fig S12) (Garcia‐Bustos *et al*, 1984; Metlitskaya *et al*, 2009). Production of McC7 was confirmed on a Kirby‐Bauer radial disk assay, by spotting the producing strain onto a lawn of wild‐type *E. coli* BL21 cells (Fig 4E).

Once McC7 biosynthesis was confirmed, the producing strain was added to the center well of the porous microplate. We seeded the wild‐type *E. coli* BL21 indicator strain in a radial pattern around the center well and the cultures were incubated for 16 h. Cell death was assayed, as before, by removing the cultures from the microplate and quantifying cell viability by optical density. We observed a greater than 50% cell death at a distance of one well away from the center microcin‐producing culture, indicative of microcin diffusion from the center, but no effect was observed two or more wells away (Fig 4E, Appendix Fig S13).

## Discussion

Designing livingmaterials where the embedded cells act as sensors, or when cells communicate tocreate differentiated patterns within the material, will require understandingchemical diffusion through the porous scaffold. This work presents a platformwith which to parameterize the transport properties of libraries of biologicalsignaling molecules through a porous matrix. The porous microplate enables asimple way to characterize distance‐dependent biological signaling parametersof inducers, quorum signals, and antibiotics. Gradients of multiple signalingevents can be explored simultaneously. The engineered receiver strain providesa single strain used to study both exogenous inducer gradients and *in situ* quorum signaling. The HEMA‐EDMAmatrix proves to be a challenging material to signal through for some inducers,based on the higher adsorption coefficients calculated in this study. Knowingthese values, we can assign different effective distances to differentsignaling channels, and envision the design of complex through‐materialcommunication networks as feedback loops, multi‐input computing, andreaction‐diffusion gradients.

Environmental factors like pH and temperature affect the interaction and stability of biological signals within materials. Attractive or repulsive interactions between signals and environment such as electrostatics, hydrogen bonding, and hydrophobic effects, can influence signal availability (Kudaibergenov *et al*, 2016; Hernandez‐Martínez *et al*, 2018). The stability of biological signals are also influenced by the environemt; for example, pH‐dependent lactonolysis of quorum signals influence quorum signaling through the soil (Yates *et al*, 2002; Politi *et al*, 2014; Gao *et al*, 2016b). These confounding factors may contribute to the observed signaling distances within this study (Fig 4A, Appendix Fig S9). Our simplified model summarizes these effects as a single “adsorption coefficient,” acknowledging that each contributing interaction is not individually accounted for. The ability to screen libraries of biological signaling events through living materials has thus far been a challenging case‐by‐case endeavor; the porous microplate and receiver strain enable a standard platform for characterization of signal transport.

## Materials and Methods

### Chemicals and materials for device fabrication

The porous microplate was fabricated using the following: 1/8“ acrylic sheets from McMaster (#8560K275); acrylic cement (Scigrip) from McMaster (#7517A2); polydimethylsiloxane (PDMS) (Sylgard™ 184) from Dow Chemicals via VWR (#102092‐312); 2‐hydroxyethyl methacrylate (HEMA) from Millipore‐Sigma (#128635); ethylene glycol dimethacrylate (EDMA) from Millipore‐Sigma (#335681); 1‐decanol from Millipore‐Sigma (#W236500); cyclohexanol from Millipore‐Sigma (#105899); 2,2‐dimethoxy‐2‐phenylacetophenone (DMPAP) from Millipore‐Sigma (#196118); 3‐(trimethoxysilyl)propyl methacrylate from Millipore‐Sigma (#440159); ethanol from Millipore‐Sigma (#E7023‐1L); acetic acid from Fisher Scientific (#A38S‐500); glass slides (70 mm × 50 mm) from VWR (#89091‐009); acetone from VWR (#BDH1101‐4LP); soda lime slide staining dish set from Electron Microscopy Sciences via VWR (#100492‐932); reagent alcohol from VWR (#BDH1164‐4LP); 37% hydrochloric acid from Millipore‐Sigma (#258148); sodium hydroxide pellets from Millipore‐Sigma (#306576); tridecafluoro‐(1,1,2,2)‐tetrahydrooctyltrichlorosilane from Millipore‐Sigma (#370517); ultraviolet handheld lamp from VWR (#89131‐492); methanol from VWR (#BDH1135‐4LP); clear straight sided jars from VWR (#10861‐940); and 10‐cm‐diameter petri dishes from VWR (#25384‐342).

### Chemicals inducers

Cells were induced with the following chemicals: 3‐oxohexanoyl‐homoserine lactone (OC6 or OC6‐HSL) ≥98% purity from Millipore‐Sigma (K3007); isopropyl β‐D‐1‐thiogalactopyranoside (IPTG) ≥99% purity from Gold Biotechnology (I2481C); anhydrotetracycline (aTc) ≥95% purity from Millipore‐Sigma (37919); L‐arabinose (Ara) ≥99% purity from Millipore‐Sigma (A3256); 3‐hydroxytetradecanoyl‐homoserine lactone (OHC14 or OHC14‐HSL) ≥96% purity from Millipore‐Sigma (51481); para‐coumaroyl‐homoserine lactone (pC‐HSL) from Millipore‐Sigma (07077); and *N*‐(3‐Oxododecanoyl)‐L‐homoserine lactone (OC12 or OC12‐HSL) from Millipore‐Sigma (O9139).

### Strains, plasmids, and media

*Escherichia coli* DH10B [∆(ara‐leu) 7697 araD139 fhuA ∆lacX74 galK16 galE15 e14‐ φ80dlacZ∆M15 recA1 relA1 endA1 nupG rpsL (StrR) rph spoT1 ∆(mrr‐hsdRMS‐mcrBC)] from New England BioLabs (C3019) was used for all routine cloning. Plasmid‐based sensor systems were characterized in *E. coli* DH10B. Receiver strains were derived from *E. coli* MG1655 (NCBI U00096.3) (Blattner *et al*, 1997; Park *et al*, 2020). *E. coli* TransforMax™ EC100D™ pir^+^ from Lucigen (ECP09500) was used to clone RK6 genomic integration vectors. *E. coli* BL21(DE) from New England BioLabs (C2527) was used for microcin cluster expression. *E. coli* K12 wild type from Yale Coli Genetic Storage Center (4404) was used for antibiotic diffusion assays. LB‐Miller broth from BD Difco (244620) was used for cloning and sequencing; SOB media from Teknova (S0210) was used for electro‐competent cell preparation. M9 media (5× M9 Salts from Millipore‐Sigma (M6030), 2 mM MgSO4 from Affymetrix (18651), 0.100 mM CaCl_2_ from Millipore‐Sigma (C1016)), supplemented with 0.4 w/v % D‐glucose from Fisher Chemical (D16‐1), and 0.2 w/v % casamino acids from BD Bacto (2233050) were used for cytometry assays.

### PDMS mold fabrication

Positive mold designs were constructed as vector images using Adobe Illustrator and transferred to a VersaLASER System 4.60 (Universal Laser Systems) loaded with a 3.18‐mm acrylic sheet. After adjusting the height of cutting table to focus the laser on the top, the acrylic sheet was laser cut in “cutting” mode. To remove any remaining dust on the acrylic surface, the molds were thoroughly rinsed with Milli‐Q® water (Millipore) (MQ‐H_2_O) and dried. Acrylic positive molds were then fixed onto glass slides with acrylic cement and allowed to cure at room temperature for at least ten minutes. Molds were then placed face up in a 10‐cm petri dish which was placed in a desiccator along with a glass slide containing 100 µl tridecafluoro‐(1,1,2,2)‐tetrahydrooctyltrichlorosilane dropped on the surface, and the desiccator was held under static vacuum for one h. The PDMS solution was prepared according to the manufacturer’s protocol and mixed in a Thinky Mixer (supplier) in batches of 55 g (1 min mixing time, 3 min degassing time). A total of 165 g mixed PDMS solution was added to the 10 cm petri dish containing the acrylic mold and degassed in a desiccator under active vacuum for 1 h, ensuring that visible air bubbles were removed from the wells of the mold. The dish containing PDMS and acrylic mold was then cured at 75°C from 3 h to overnight. The solidified negative PDMS molds were cut out using a razor blade and carefully detached from the acrylic. Isopropanol was sporadically added to the PDMS–acrylic interface to aid in separation.

### Glass slide preparation

Device fabrication was adapted from previous methods (Geyer *et al*, 2011; Ge *et al*, 2016). Glass slides were submerged in reagent alcohol in the soda lime chamber for 30 min, rinsed with acetone, and dried with a N_2_ stream. Slides were then activated by submersion in the following sequence: MQ‐H_2_O, 5 min; fresh MQ‐H_2_O, 5 min; 1 M sodium hydroxide, 60 min; MQ‐H_2_O, 5 min; 1 M hydrochloric acid, 30 min; MQ‐H_2_O, 5 min; 1 M sodium hydroxide, 60 min; MQ‐H_2_O, 5 min; 1 M hydrochloric acid, 30 min; and MQ‐H_2_O, 5 min. Slides were then dried with a N_2_ gas stream (AirGas) and placed flat on the benchtop. A fresh 5.0 ml solution was prepared of 3‐(trimethylsilyl) propyl methacrylate (20% by weight) in ethanol, and the pH was adjusted to 5.0 with approximately 250 µl acetic acid. A 100 µl volume of 3‐(trimethylsilyl) propyl methacrylate solution was added to a first glass slide, and a second glass side was sandwiched on top and incubated for 30 min. The slides were separated, the treated sides were washed with acetone and dried with a Kimwipe, and methacrylate treatment was re‐applied in the same manned to the same sides. The slides were again washed with acetone and dried until visually transparent.

### HEMA‐EDMA cross‐linking

A precursor solution of HEMA‐EDMA was prepared by stirring with a stir bar the following in a glass jar protected from light at the following weight % ratio (unless otherwise noted): HEMA (24 wt %); EDMA (16 wt %); 1‐decanol (12 wt %); cyclohexanol (48 wt %). Next, DMPAP (1 wt %) was dissolved into the mixture by stirring. A PDMS mold placed face up was filled with precursor HEMA‐EDMA solution, and a glass slide was gently sandwiched on top, ensuring that no bubbles were formed. The sandwiched device was illuminated with (365 nm) ultraviolet for 15 min; the device was inverted, and re‐illuminated for 15 min. The PDMS mold was gently separated from the solidified HEMA‐EDMA device. The solidified device was added to 300 ml methanol in a glass incubation jar to wash out the solvents and incubated at room temperature for at least 16 h. The methanol was then discarded and exchanged for fresh methanol, and the device was incubated again for at least 16 h; the methanol was exchanged once more, the device incubated for at least 16 h and remained stored in methanol until needed.

### Porosity measurements

HEMA‐EDMA blocks of 1 cm^3^ were fabricated as described. After washing in methanol, blocks were transferred to pre‐sterilized 250 ml Milli‐Q® water (Millipore) (MQ‐H_2_O) in glass incubation jars and incubated at room temperature for 48 h. After incubation, devices were transferred to a 10‐cm petri dish and the glass platform of the device was dried with a laboratory wipe. The hydrated mass of the device + petri dish was recorded using a gravity balance. The device was then dehydrated by baking at 80°C for 72 h, and the dehydrated mass of the device + petri dish was measured again. The measurements were thrice repeated with separate devices. The porosity *ε* was calculated as follows:

(3) $$M_{\text{total}}=V_{\text{total}}\left[\varepsilon \rho_{\mathrm{aq}}+\left(1‐\varepsilon \right)\rho_{\text{HEMA - EDMA}}\right]$$

(4) $$M_{\text{HEMA - EDMA}}=V_{\text{total}}\left[\left(1‐\varepsilon \right)\rho_{\text{HEMA - EDMA}}\right]$$

(5) $$M_{\mathrm{aq}}=V_{\text{total}}\;\varepsilon \;\rho_{\mathrm{aq}}$$

where *M* is mass, *V* is volume of the device, *ρ* is density, and *ε* is porosity.

### Scanning electron microscopy

A sample of polymerized HEMA−EDMA was taken out from a methanol jar, and any remaining solvent was removed by air drying for > 1 week. The sample was attached to a stub, loaded on MERLIN SEM (Zeiss) and imaged with SmartSEM software (Zeiss) with 100 – 16 270 times of magnification at the Electron Microscopy Facility in the Massachusetts Institute of Technology Materials Research Science and Engineering Centers (MIT MRSEC).

### Dye permeability experiments

Molecular transport was measured using the following chemicals: methyl blue (cotton blue, CAS #28983‐56‐4) from Millipore‐Sigma (M6900); rhodamine‐b base (CAS #509‐34‐2) from Millipore‐Sigma (234141). Cotton blue is considered water soluble, while rhodamine‐b base is considered alcohol soluble (Chemicalbook.com). After incubation in methanol, microplate devices were added to glass jars containing 250 ml autoclaved MQ‐H_2_O and incubated at room temperature for 48 h. Water was removed from the wells of the microplate using a p20 pipette. An aqueous 15.7 mM solution of dye was added to the center well at a volume of 10 µl (Fig 3A and C). Sterile MQ‐H_2_O was added to the remaining wells at a volume of 10 µl. The microplate was sealed in a makeshift incubation chamber made from an empty p100 disposable pipette tip box containing 20 ml MQ‐H_2_O in the bottom reservoir. The sealed tip box was incubated at 30°C for 24 h in a VWR benchtop shaking incubator with no shaking. After incubation, the adsorption of 1.25 µl of dye solution from each well was measured using a NanoDrop 1000 (600 nm for methyl blue and 554 nm for rhodamine). The adsorption values were converted into millimolar concentration using a standard curve.

### Electro‐competent cell preparation

A single colony of the strain to be transformed was inoculated into 2 ml SOB (with antibiotics when needed) and grown overnight at 37°C and 250 r.p.m. in a New Brunswick Innova 44 Shaker (Eppendorf, USA). Overnight cultures were diluted 1:250 into SOB (with antibiotics when needed) and grown for roughly 2 h. When the early exponential phase was reached (OD_600_ = 0.3–0.6), cultures were placed on ice for 10 min, centrifuged (4,000 *g*, 4°C, 15 min), and thrice washed with ice‐cold 10 % glycerol. After the third wash, the culture was resuspended in ice‐cold 10% glycerol at 1/100^th^ the original diluted culture volume. Cultures were immediately electroporated with DNA payloads at 2500 V (Eppendorf).

### Optimization of luxR, rpaR, and lasR

The sensors in p15A plasmids were transformed into *E. coli* NEB DH10b for negative selection. During negative selection, each quorum activator was cultured either in the absence of inducer or in the presence of one of the three mismatched HSL inducers. The activation of the quorum output promoter was considered “leaky” and the expressed PheS protein facilitated negative selection in the presence of 4‐chloro‐DL‐phenylalanine. Following transformation and outgrowth, cultures diluted into 7 ml LB media with antibiotics, Cl‐Phe and cross‐reactive inducers. Cultures were grown at 37°C for 16 h. Once the leaky quorum activator variants were selected against, the cells were cultured in the presence of the proper cognate HSL inducer, which drives DNAPB expression. Following negative selection, cultures were diluted 1:100 into 2 ml LB media with antibiotics in culture tubes and grown at 37°C, 250 r.p.m. for 2 h in preparation for positive selection. Positive selection was carried out as described in detail in Meyer *et al* (Liu *et al*, 2018). In brief, the cultures were then diluted 1:100 into 2 ml prewarmed LB media with antibiotics and inducers and grown at 37°C, 250 r.p.m. for 4 h. Following induction, 1 ml of culture was centrifuged at (5,000 *g*, 25°C, 10 min). Supernatant was removed, and the cell pellet was resuspended in 50 μl 1x CPR buffer (50 mM Tris–HCl pH 8.8, 10 mM KCl, 2 mM MgSO_4_, 10 mM (NH_4_)_2_SO_4_). The resuspended cells were emulsified with degenerate PCR primers (Meyer *et al*, 2019), and the DNA was purified using Zymo Spin I columns. The library was amplified in a recovery PCR using Accuprime *Pfx* and nested primers, gel purified, and assembled by Golden Gate Assembly (Weber *et al*, 2011).

### Library shuffling

Between some rounds of selection, libraries were shuffled upon themselves (Meyer *et al*, 2014). From the recovery PCR, 1 μg linear library DNA was added to a mild DNAse reaction (500 mM Tris pH 7.4, 100 mM MnCl_2_, 0.5 U DNAse from New England Biolabs and lightly digested for 3 minutes at 15°C. Fragmented DNA was purified using Zymo Spin I columns and reassembled in a primer‐less PCR in 1 × KAPA HiFi Master Mix form KAPA Biosystems by thermal cycling (95°C for 2 min; 35 cycles of [95°C for 30 s, 65°C for 90 s, 62°C for 90 s, 59°C for 90 s, 56°C for 90 s, 53°C for 90 s, 50°C for 90 s, 47°C for 90 s, 44°C for 90 s, 41°C for 90 s, 68°C for 90 s]; and 72°C for 4 min). The reassembly was purified using Zymo Spin I columns, reamplified using Accuprime *Pfx* and CPR primers, gel purified, and assembled as described above.

### Construction of sentinel strain sCV.5073

Landing pad strains and payload delivery are described by Park *et al* (2020). The strain containing the empty landing pads was co‐transformed by electroporation with 0.5–1.0 µg of a plasmid containing three integrases (pYJP.053) and 0.5–1.0 µg of a plasmid containing the DNA payload (pCAV5.063). Immediately after electroporation, 500 µl SOB media were added to the cuvette. The culture was incubated at 30°C for 3 h and plated in its entirety on LB with 1.5% agar plates with tetracycline (5 µg/ml) and incubated overnight at 37°C. Individual colonies were inoculated in 500 µl SOB media with tetracycline (5 µg/ml) and incubated overnight at 37°C.

### Cytometry analysis

Fluorescence characterization with cytometry was performed using a BD LSR Fortessa flow cytometer with HTS attachment (BD, Franklin Lakes, NJ, USA). Cells diluted in PBS with kanamycin were run at a rate of 1.0 μl s^−1^. Measurements were made using a green laser (488 nm) voltage of 400 V, an FSC voltage of 640 V, and an SSC voltage of 289 V. A total of 5 × 10^4^ gated events were collected and used for analysis. For each sample, the median YFP fluorescence was calculated. For a given promoter measurement, the sensor strain is transformed with an output plasmid (Fig S8A). The median autofluorescence value is subtracted from the all other median fluorescence values, including that of the RPU standard. The experimental sample value is then divided by the RPU standard value. For output promoter transfer functions, each response curve was fit to a Hill equation:

(6) $$y=y_{\text{min}}+\left(y_{\text{max}}‐y_{\text{min}}\right)\left(\frac{x^{n}}{K^{n}+x^{n}}\right)$$

where y is the promoter activity in RNAP/s, x is the concentration of the small molecule, y_min_ is the leakiness, K is the threshold (sensitivity), and *n* is the cooperativity. The data were fit using the solver function in Microsoft Excel, and the coefficients fit to Equation 1 are found in Table S1.

### Response function measurements

Glycerol stocks of strains containing the plasmids of interest were streaked on LB with 1.5% agar plates and grown overnight at 37°C. Single colonies were inoculated into 400 µl M9 media supplemented with glucose, casamino acids, and antibiotics in 2 ml 96‐deep‐well plates (USA Scientific, Orlando, FL, USA) sealed with an AeraSeal film (Excel Scientific, Victorville, CA, USA) and grown at 37°C at 900 r.p.m. overnight in a Multitron Pro shaker incubator (INFORS HT, Bottmingen, Switzerland). The overnight growths were diluted 1:200 into 1 ml LB with antibiotics in 96‐well V‐bottom plates (Nunc #249952) sealed with AeraSeal film and grown at 37°C, 1000 r.p.m. in a benchtop shaking incubator. After 2 h, the cultures were diluted 1:500 into M9 media supplemented with glucose, casamino acids, antibiotics, and inducer where necessary, in 96‐well V‐bottom plates sealed with AeraSeal film and grown at 37°C, 1000 r.p.m. for 5 h. After growth, 5 μl of culture sample was diluted into 195 μl PBS with 200 μg/ml kanamycin.

### Conversion of fluorescence arbitrary units to RNAP flux

The reference promoter BBa_J23101 has been defined as having an activity of 1 RPU (Canton *et al*, 2008). We can define the total promoter activity as the RNAP flux from all copies of a promoter in the cell. We have previously measured BBa_J23101 to produce a total promoter activity of 0.38 RNAP/s when carried on a p15a plasmid (with an average 13.2 copy number) and when transcriptionally fused to the BB0064 RBS, in *E. coli* MG1655 (Shao *et al*, 2021). In the current work, we use an identical p15A plasmid backbone and RBS fused to all output promoters tested, and we normalize the fluorescence output to an identical RPU standard plasmid (Appendix Fig S6)—therefore we assume a conversion factor of 1 RPU = 0.38 RNAP/s, and report our date using the RNAP/s units throughout the text.

### Inducer diffusion experiments

After incubation in methanol, microplate devices were added to glass jars containing 250 ml pre‐sterilized M9 media supplemented with glucose, casamino acids, and antibiotics, and incubated at room temperature for 48 h. The strains to be tested were cultured overnight from glycerol stocks in a Multitron Pro shaker as described in “response function measurements”. The overnight growths were diluted 1:200 into 1 ml LB with antibiotics in 96‐well V‐bottom plates sealed with AeraSeal film and grown at 37°C, 1000 r.p.m. in an ELMI Digital Thermos Microplates shaker incubator (Elmi Ltd, Riga, Latvia). After 2 h, the cultures were diluted 1:500 into M9 media supplemented with glucose, casamino acids, antibiotics, and inducer (when required) in 2 ml 96‐deep‐well plates. The wells of the hexagonal porous multi‐well culture plate were emptied of media by pipetting out using a 250 µl pipette. Immediately, 12 µl of the diluted cell cultures was added to all wells except for the center well. For pure chemical inducer diffusion: to the center well, 12 µl of an inducer cocktail diluted M9 media supplemented with glucose, casamino acids, and antibiotics was added (20.0 mM IPTG, 0.0004 mM aTc, 160.0 mM L‐arabinose, 0.1 mM OHC6‐HSL, 0.1 mM pC‐HSL, 0.4 mM OHC12‐HSL). For quorum communication experiments: 12 µl diluted sender cells and inducer were added to the center well (1.0 mM IPTG). The “incubation chamber” reservoir was filled with 20 ml MQ‐H_2_O, the porous multi‐well plate was added to the platform, and the “incubation chamber” was sealed with Parafilm. The “incubation chamber” was then incubated at 37°C, 500 r.p.m. in an incubating microplate shaker (VWR #97043‐606). After 5 h, 10 µl of the cultures was diluted into 190 µl PBS + 200 μg/ml kanamycin.

### Modeling and simulations

Numerical simulation was carried out with COMSOL 5.5 Multiphysics (COMSOL) using the “Effective Diffusivity in Porous Materials” module and a Freundlich adsorption factor. The microplate design was imported into COMSOL from the appropriate vector images (Appendix Fig S1). The mesh element size was calibrated for fluid dynamics with the predefined extremely fine settings. To provide a good transition to other regions and avoid numerical instabilities due to the drastic concentration difference at *t* = 0 between the central well and the hydrogel, each side of the hexagonal interface was refreshed with a distribution of 200 elements. Effective diffusivity (D_e_) was modeled within the HEMA‐EDMA walls according to Equation 1 where D_aq_ is the diffusion coefficient in water (m^2^ s^−1^), ε is the porosity, and τ is the tortuosity factor. We used published values for D_aq_ of glycine (1.1 × 10^−9^ m^2^ s^−1^) and D_e_ of glycine through HEMA‐EDMA (3.47 × 10^−10^ m^2^ s^−1^) (Stewart, 2003; Ge *et al*, 2016), and our experimentally determined porosity fraction ε = 0.6 to calculate the tortuosity τ = 1.9. Diffusion was modeled in the wells using for D_aq_ of glycine (1.1 × 10^−9^ m^2^ s^−1^). We simulated 5 h of transport for each inducer and converted the resulting concentration profile into projected RNAP/s values using Equation 6. Simulated promoter activity for each inducer was compared with the measured values at the distance that corresponds to the center of each measured well, and a correlation coefficient was determined in excel. The simulation that resulted in the highest correlation coefficient was selected as the best‐match. The best‐match adsorption coefficients and correlation coefficients are found in Table 2. ClogP values of each inducer were calculated using ChemDraw Professional (Perkin Elmer). The output of the simulation is available in the Appendix.

### Microcin producer strain construction and cluster expression conditions

Genes *mccA‐E* and *mccF* encoding microcin C7 biosynthesis were amplified from plasmid pp70 (Piskunova *et al*, 2017). Genes were cloned into plasmid pAMK.106, which contains a ColE1 origin of replication and resistance to tetracycline and chloramphenicol, to generate plasmid pAMK.164. The *mcc* genes were cloned downstream of a T7lacO promoter for IPTG‐inducible cluster expression and transformed into *E. coli* BL21(DE3) for expression. For assaying antimicrobial activity, plates of LB agar were streaked with an inoculum (1:100 dilution of OD_600_ = 0.1) of *E. coli* BL21(DE3) harboring pAMK.106. This serves as an indicator strain for assaying antimicrobial activity. The strain harboring pAMK.164 was grown in 10 ml of LB media in a TPP® TubeSpin bioreactor tube from Millipore‐Sigma (Z761028‐180EA) at 37°C, 250 r.p.m. in an Innova 44 Shaker (Eppendorf, USA) in LB (no antibiotic) until OD_600_ = 1. The culture was diluted 1:100 in 10 ml and grown in the same conditions for 3 h at 30°C, then cluster expression was induced with 1 mM IPTG. A 15 µl aliquot of induced culture was spotted on plates containing indicator strain, allowed to dry at room temperature, then incubated with inversion for 20 h at 30°C.

### Antibiotic diffusion experiments

Glycerol stocks of strains containing the plasmids of interest were streaked on LB + 1.5% agar plates and grown overnight at 37°C. Single colonies were inoculated into 400 µl LB with antibiotics (and for the microcin producer strain harboring pAMK.164, 1 mM IPTG) and cultured overnight in a Multitron Pro shaker. The overnight cultures were diluted the next day (for indicator strain *E. coli* K12, 1:500; for indicator strain *E. coli* BL21 (DE3) harboring pAMK.106, 1:500; for microcin producer strain *E. coli* BL21 (DE3) harboring pAMK.164, 1:50 into LB containing 1 mM IPTG, and added into the wells of the hexagonal porous multi‐well culture plate. For pure antibiotic solution experiments: To the center well, 10 µl of a single antibiotic solution was added (2.0 mg ml^−1^ carbenicillin, 0.5 mg ml^−1^ kanamycin, 0.25 mg ml^−1^ tetracycline, or a control solution of MQ‐H_2_O). To the remaining wells, 10 µl indicator strain *E. coli* K12 was added. For microcin producer experiments: To the center well, 10 µl of microcin producer strain was added, and to the remaining wells, 10 µl of indicator strain *E*. *coli* BL21 was added. The makeshift incubation chamber reservoir was filled with 20 ml MQ‐H_2_O, the porous multi‐well plate was added to the platform, and the “incubation chamber” was sealed with Parafilm. The “incubation chamber” was then incubated at 37°C, 500 r.p.m. in a benchtop shaker. After 16 h, 10 µl of the cultures was transferred to a polystyrene V‐bottom 96‐well plate for optical density analysis.

### Optical density analysis

The OD_600_ of the cultures was analyzed using a Shimadzu UV‐Vis spectrophotometer and a 5 µl rectangular UV quartz cuvette (Agilent #5063‐6564). LB media was used to blank the spectrophotometer, and cultures were analyzed undiluted and 1:10 diluted in LB media. OD_600_ value measurements of each well were normalized by dividing by the combined average OD_600_ value of all wells in the negative control. The normalized OD_600_ values of at least three wells of equal distance from the center of the porous multi‐well were averaged together.

## Author contributions

**Christopher A Vaiana:** Conceptualization (equal); Data curation (equal); Formal analysis (equal); Investigation (equal); Methodology (equal); Writing—original draft (equal); Writing—review & editing (equal). **Hyungseok Kim:** Data curation (equal); Investigation (equal). **Jonathan Cottet:** Data curation (equal); Software (equal); Formal analysis (equal); Writing—original draft (equal); Writing—review & editing (equal). **Keiko Oai:** Investigation (equal). **Zhifei Ge:** Methodology (equal). **Kameron Conforti:** Conceptualization (equal); Software (equal). **Andrew M King:** Investigation (equal). **Adam J Meyer:** Investigation (equal). **Haorong Chen:** Investigation (equal). **Christopher A Voigt:** Conceptualization (equal); Resources (equal); Supervision (equal); Funding acquisition (equal); Validation (equal); Writing—original draft (equal); Writing—review & editing (equal). **Cullen R Buie:** Conceptualization (equal); Resources (equal); Formal analysis (equal); Funding acquisition (equal); Validation (equal); Writing—original draft (equal); Writing—review & editing (equal).

## Supporting information



Appendix

Supplementary Dataset S1

## Data Availability

Modeling data outputs provided in the Appendix.
