# Supplementary material for: Characterizing chemical signaling between engineered “microbial sentinels” in porous microplates
Source: Mol Syst Biol. 2022 Mar 22;18(3):e10785. doi: 10.15252/msb.202110785 (PMC8938921; doi:10.15252/msb.202110785)
Supplement: Supplementary file 1 — Appendix [file MSB-18-e10785-s002.pdf]

Appendix for:

Characterizing chemical signaling between engineered "microbial sentinels" in porous microplates

Christopher A. Vaiana[1,2], Hyungseok Kim[1], Jonathan Cottet[1], Keiko Oai[1], Zhifei Ge[1], Kameron Conforti[1], Andrew King[2,] Adam Meyer[2], Haorong Chen[2], Christopher A. Voigt[2*], Cullen R. Buie[1*]

[1] Department of Mechanical Engineering, Massachusetts Institute of Technology, Cambridge, MA, USA
[2] Synthetic Biology Center, Department of Biological Engineering, Massachusetts Institute of Technology, Cambridge, MA, USA

Table of contents:

Appendix Figure S1: Hexagonal microplate fabrication…… 02
Appendix Figure S2: Alternate microplate designs…… 03
Appendix Figure S3: Scanning electron micrographs…… 04
Appendix Figure S4: Quorum sensor directed evolution…… 05
Appendix Figure S5: Sensor array construction and plasmid map…… 07
Appendix Figure S6: Output plasmid and RPU standard plasmid maps ……08
Appendix Figure S7: Sensor orthogonality measurements……09
Appendix Figure S8: Representative inducer diffusion cytometry measurements……11
Appendix Figure S9: Inducer molecule ClogP analysis……13
Appendix Figure S10: Quorum sender and receiver plasmid maps…… 14
Appendix Figure S11: Antibiotic diffusion data…… 15
Appendix Figure S12: Microcin producer plasmid map ……17
Appendix Figure S13: Microcin producer diffusion data……18
Appendix Table S1: Sequence of genetic parts used in this study……19
Appendix Table S2: Quorum sensor activator proteins ……22
Appendix Table S3: Sequence of plasmids used in this study …… 23
Appendix References……32

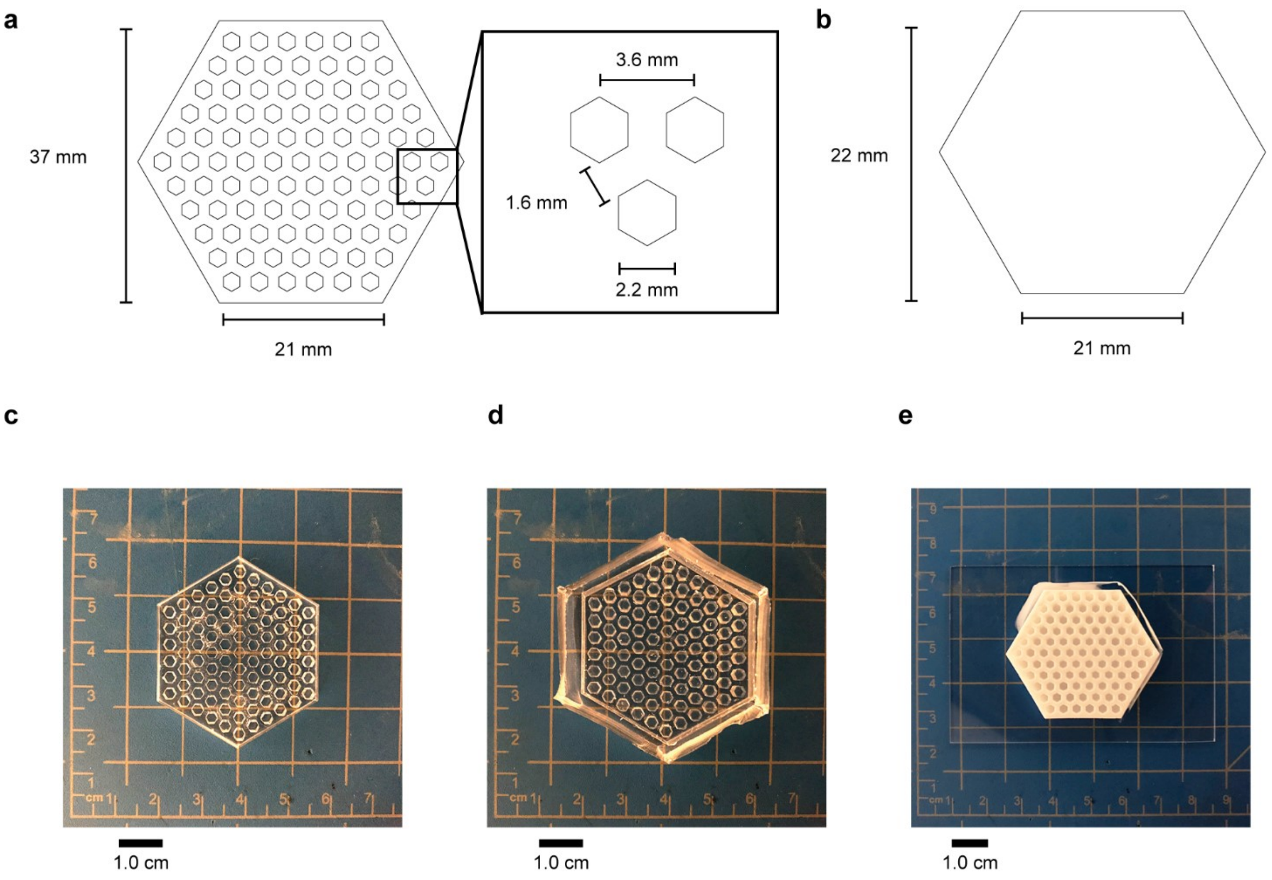

Appendix Figure S1: Hexagonal microplate fabrication. (a) The dimensions of the hexagonal plate wells are shown overlaid on a 1x scaled vector image template used for laser cutting the acrylic mold. Inset show the well center-to-center distance, well diameter, and the wall thickness between wells. **(b)** A vector image template of the hexagonal plate bottom used for lased cutting an acrylic mold**. (c)** Photograph of the final acrylic mold. **(d)** Photograph of the PDMS master. **(d)** The final cross-linked microplate affixed to an activated 2” x 3” glass slide. Scale bar dimensions are noted in the figure.

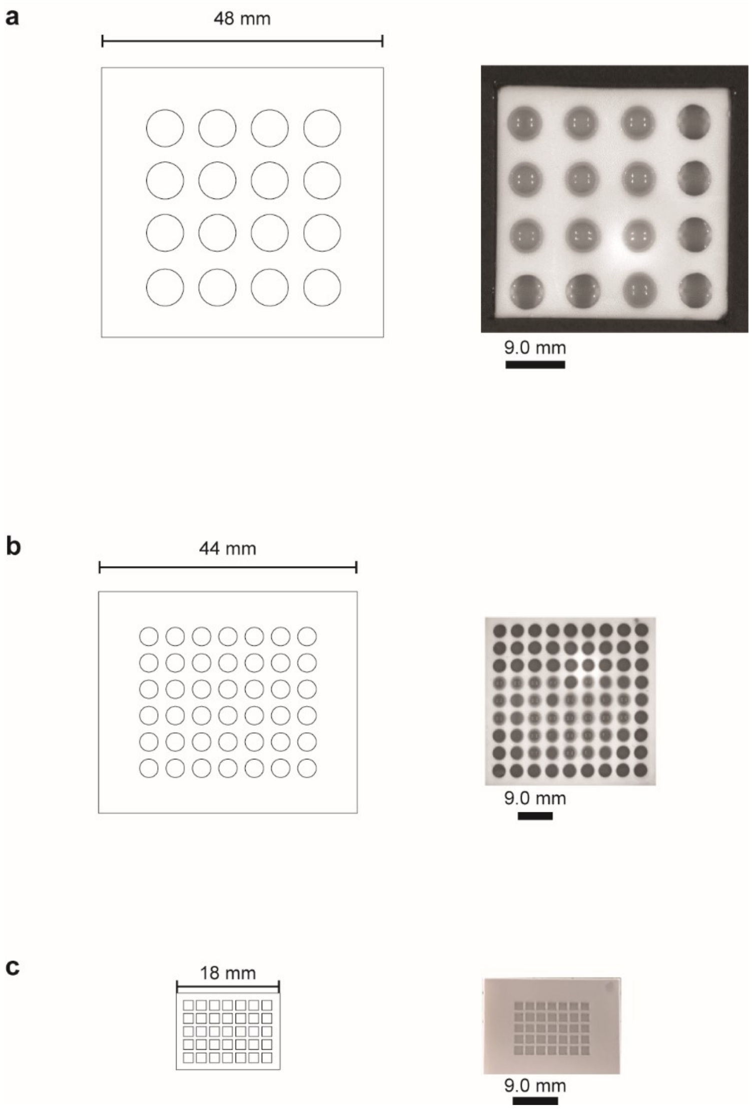

Appendix Figure S2: Alternate microplate designs. Vector image templates and photographs of corresponding microplates with the dimensions of a 96-well **(a)**, 384-well **(b)**, and 1536-well standard plate **(c)**. Scale bar dimensions are noted in the figure.

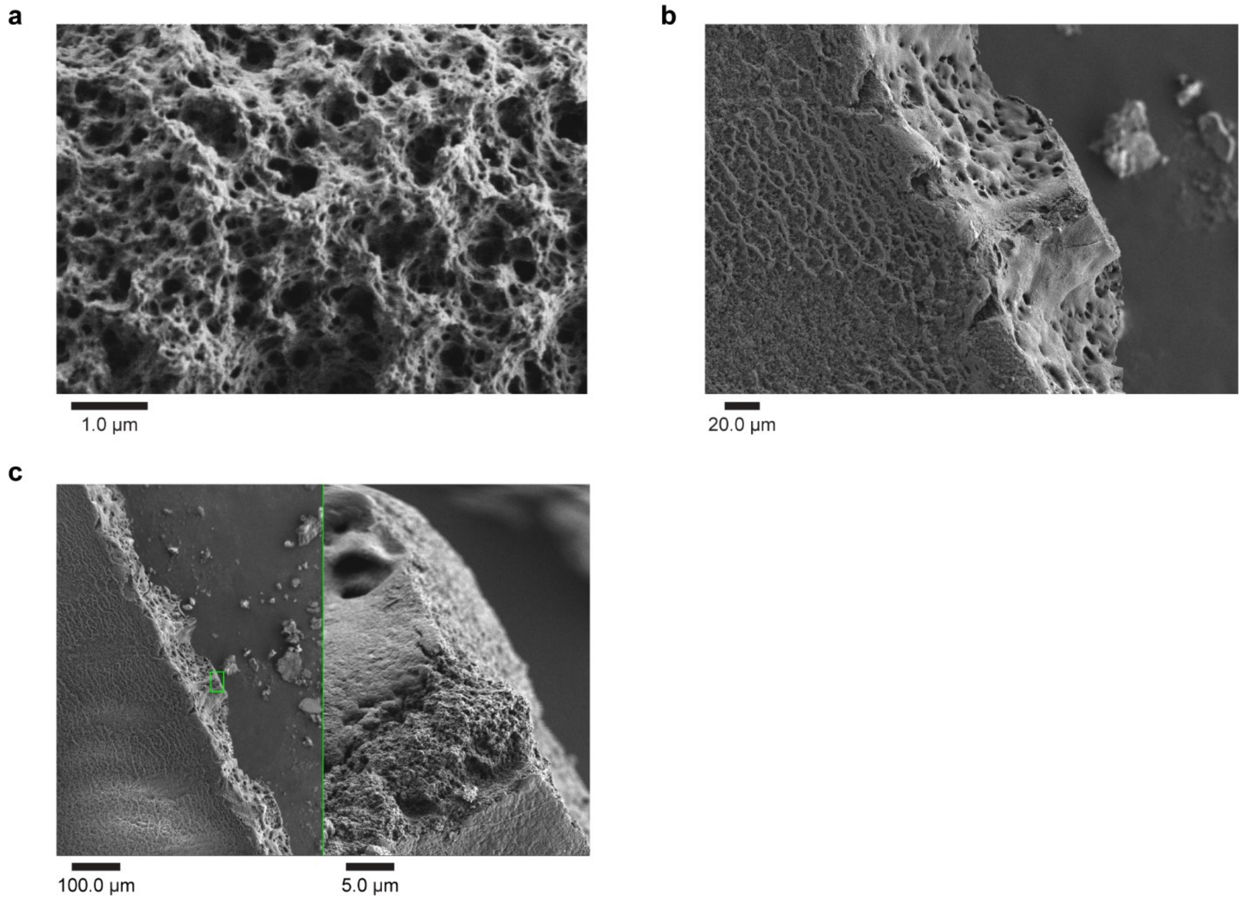

Appendix Figure S3: Scanning electron micrographs. Magnification: 116 270 x magnification **(a)**; 372 x magnification **(b)**; 100 x magnification (left), 2 000x (right) **(c)**.

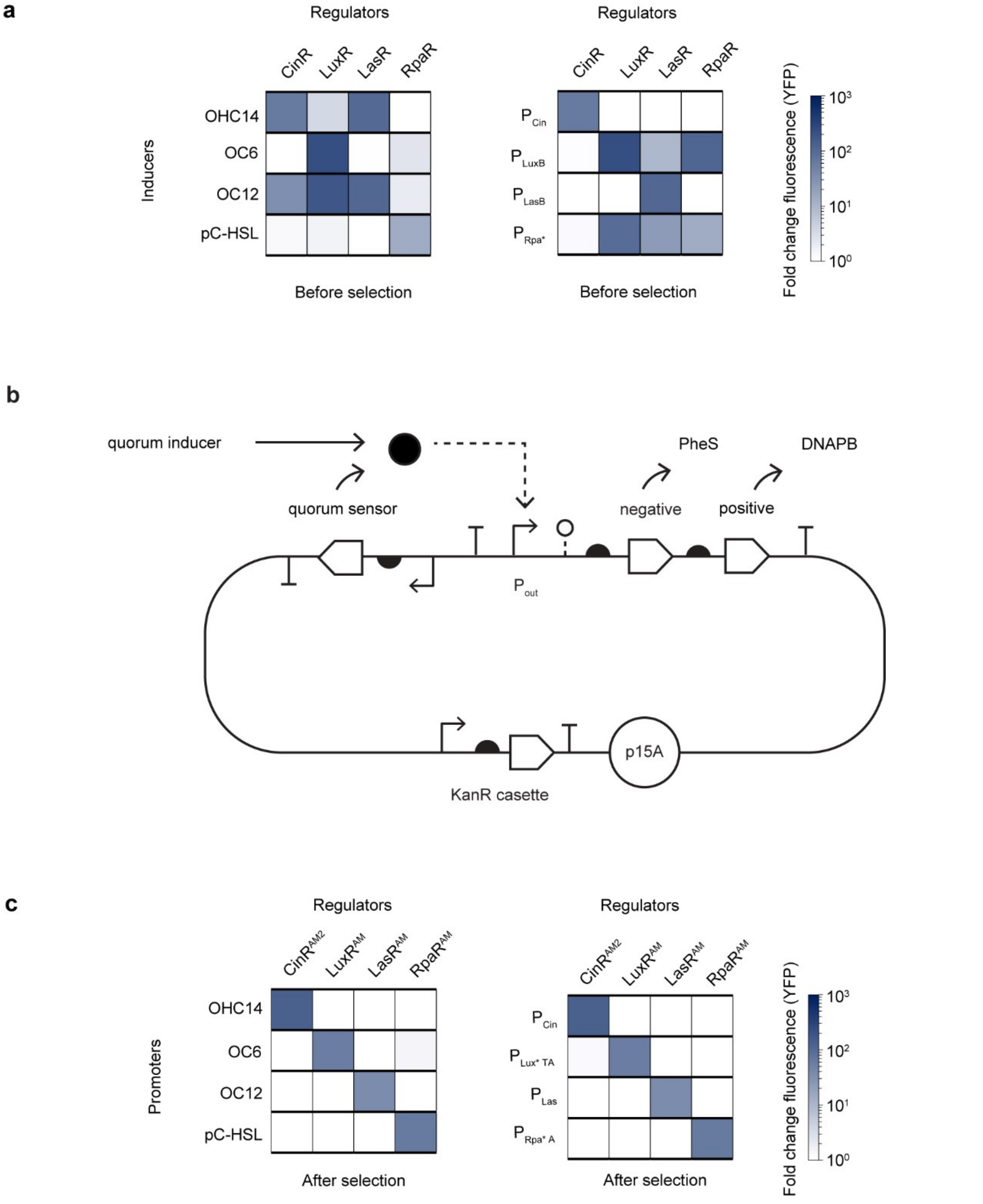

Appendix Figure S4: Quorum sensor evolution. **(a)** Chemical (left) and promoter (right) cross reactivity of *cinR, luxR, lasR*, and *rpaR* before evolution. **(b)** Selection plasmid map. **(c)** Chemical (left) and promoter (right) cross reactivity of the final sensors after evolution, denoted as $cinR^{AM}$, $luxR^{AM}$, $lasR^{AM}$, and $rpaR^{AM}$. Fold change equals the fluorescence signal of a strain bearing an output plasmid (Appendix

Figure 6) with YFP under the control of P_{out} cultured with its cognate inducer divided by the fluorescence signal of that strain with no inducer present.

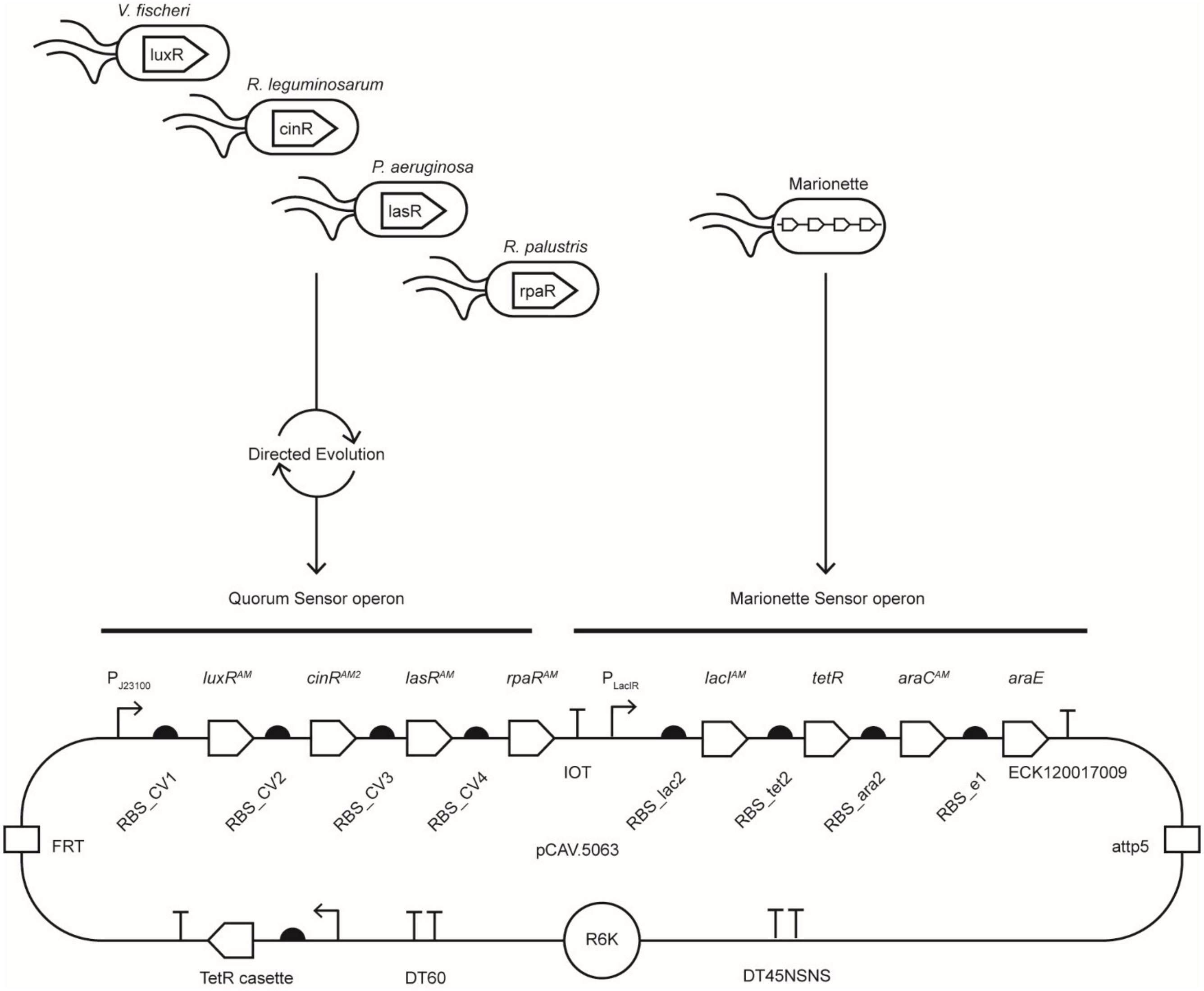

Appendix Figure S5: Sensor array construction and plasmid map. The evolved quorum sensors *LuxR*^{AM}, *CinR*^{AM2}, *LasR*^{AM}, and *RpaR*^{AM} were cloned from their respective plasmids into a single operon controlled by the constitutive promoter J23100 and rationally designed synthetic RBS sequences. The sensor block *LacI*^{AM}, *TetR*^{AM}, and *AraCE*^{AM} were cloned directly from the Marionette-wild strain and remained under control of the constitutive promoter LacI and rationally designed synthetic RBS sequences. The entire sensor block was cloned onto an R6K genomic integration plasmid with a tetracycline resistance cassette.

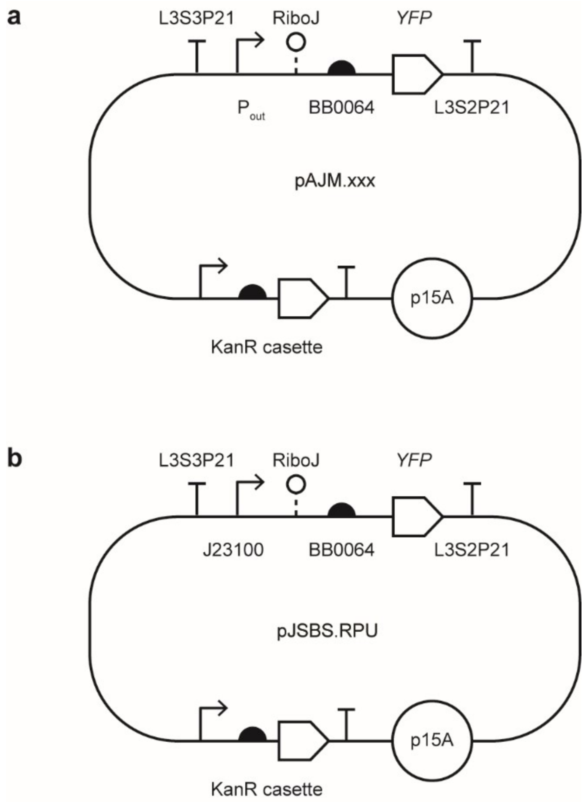

Appendix Figure S6: Output and RPU standard plasmid maps. (a) Output plasmids were used for dose-response characterization and for inducer gradient detection within the porous microplate. All outputs are assembled onto the same architecture and the promoter P_{out} represents a single output promoter that corresponds with each of the sensors in the Sensor strain. Sequences of each P_{out} can be found in Supplementary Table 1. The complete sequence of the following plasmids have been published prior: pAJM.715 (P_{Tac}), pAJM.716 (P_{Bad}), pAJM.717 (P_{Tet}) (Meyer *et al*, 2019). The complete sequences of the following plasmids can be found in Supplementary Table 2: pAJM.944 (P_{Cin}); pAJM.947 (P_{Rpa}); pAJM.1246 (p_{Lux}); pAJM.1349 (P_{Las}); pAJM.947 (P_{Rpa}). **(b)** The RPU plasmid was used for normalizing the YFP fluorescent outputs into standardized units, and the full sequences has been previously published (Meyer *et al.*, 2019).

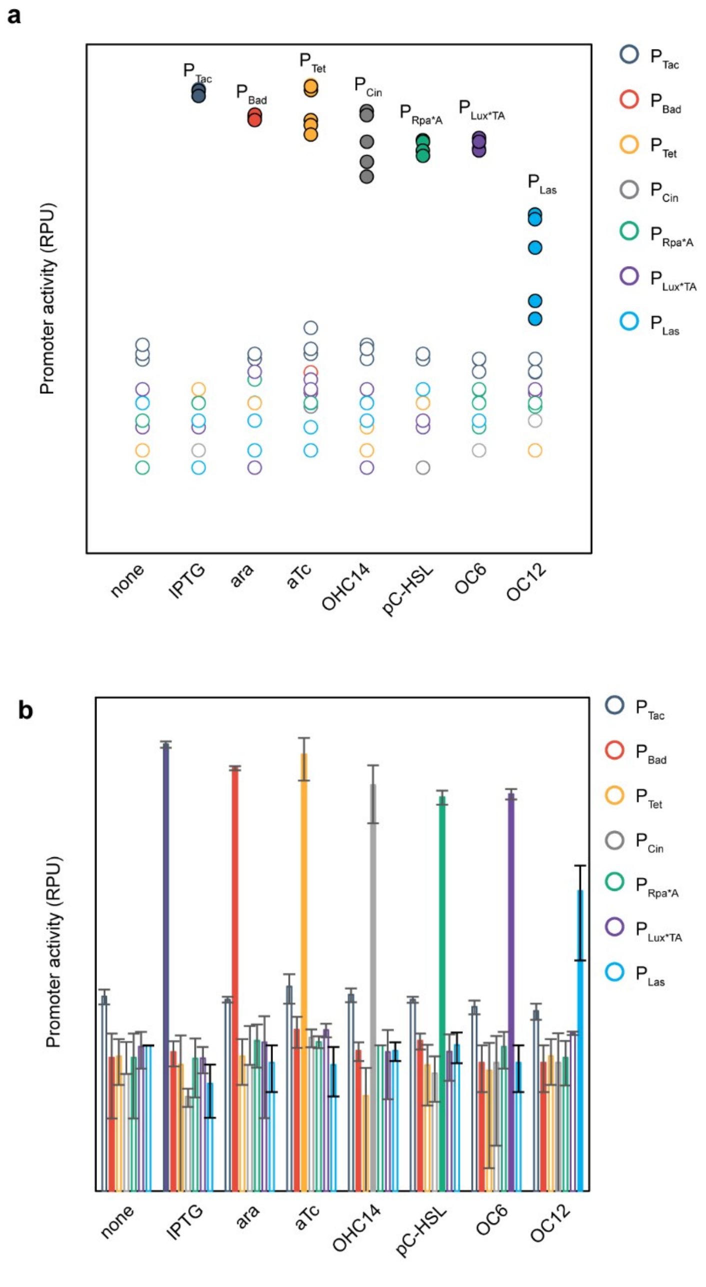

Appendix Figure S7: Sensor orthogonality screen. Each output strain was cultured with each of the seven small molecule inducers. **(a)** All data points are plotted with inducer on the X axis and promoter activity (RPU) on the Y axis for each combination of output strain and inducer. The open circles indicate data for mis-matched output strain – inducer pairs. The labeled closed circles indicate data for the proper output strain – inducer pair. At least three data points were taken for each condition. Data that were negative (and suggests no signal was present) after normalization into RPU units were not included. **(b)** The compiled data from part **(a)** as the average signal for each output strain – inducer pair.

Data bars are the average of at least three independent culture wells of each output-inducer pair tested on different days. The error bars represent the standard deviation. Data that were negative (and suggests no signal was present) after normalization into RPU units were not included.

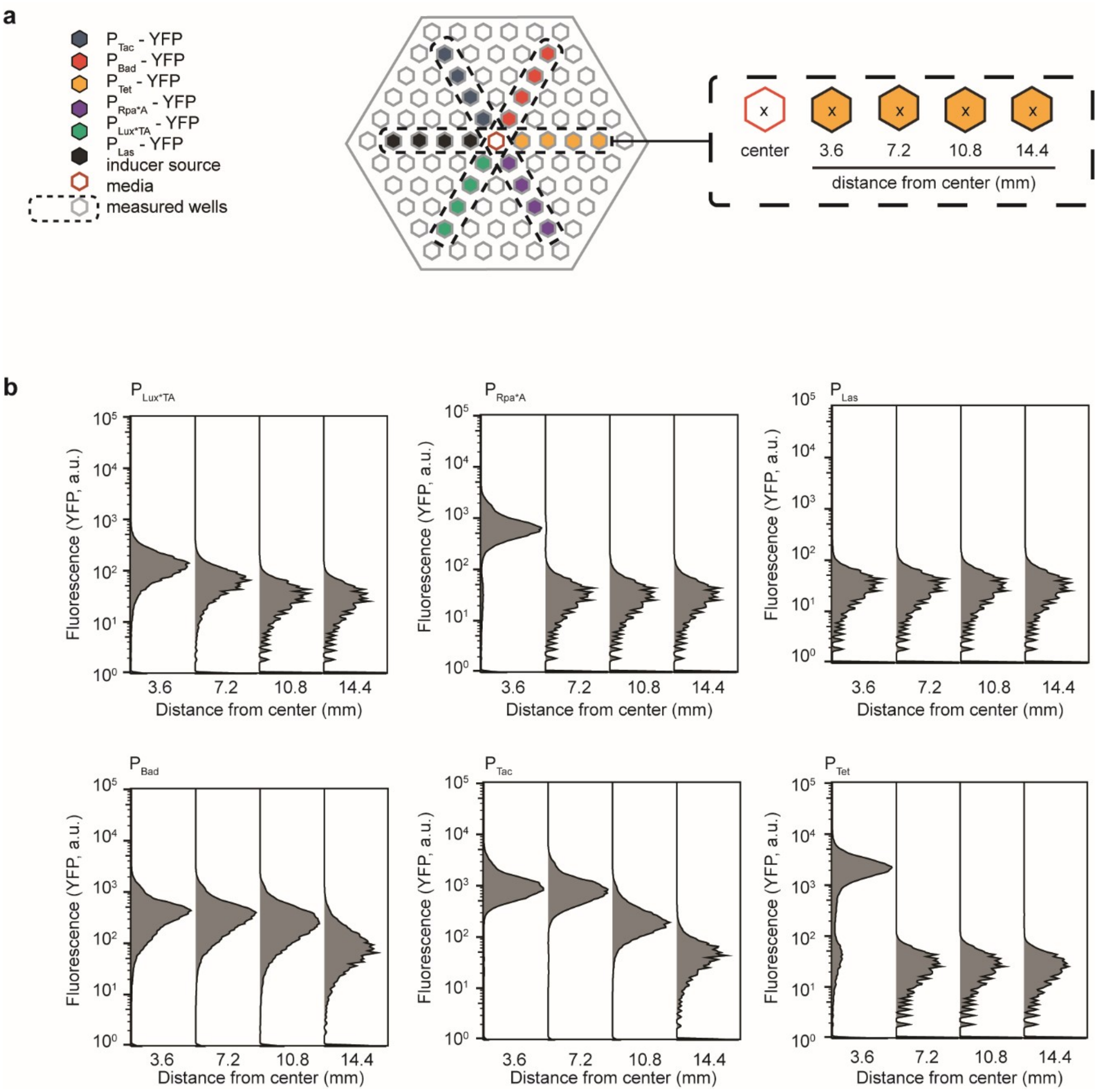

Appendix Figure S8: Representative inducer diffusion cytometry data. (a) Schematic depiction of the porous microplate setup for inducer diffusion experiments. The center well was loaded with an inducer cocktail (red open hexagon; (20.0 mM IPTG, 0.0004 mM aTc, 160.0 mM L-arabinose, 0.1 mM OC6, 0.1 mM pC-HSL, 0.4 mM OC12). The neighboring wells were loaded with output strains as pictured (filled colored hexagons, clockwise from the top left: pAJM.715 (P_{Tac}); pAJM.716 (P_{Bad}); pAJM.717 (P_{Tet}); pAJM.947 (P_{Rpa*A}); pAJM.1246 (P_{Lux*TA}); pAJM.1349 (P_{Las}). The output strain pAJM.944 (P_{Cin}) was tested separately and yielded no signal (data not shown). The remaining wells contained growth medium (open black hexagons). The inset illustrates the sampled wells and their respective distance from the center source well. **(b)** Single representative cytometry data set after a five hour incubation time. The median

fluorescence signal was recorded and normalized for data analysis, and the experiment was repeated three times on different days.

a

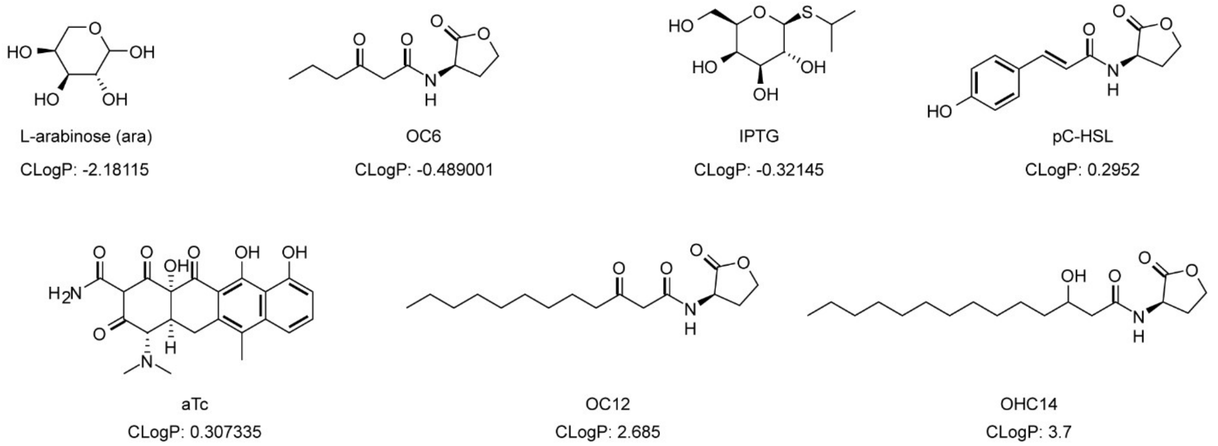

b

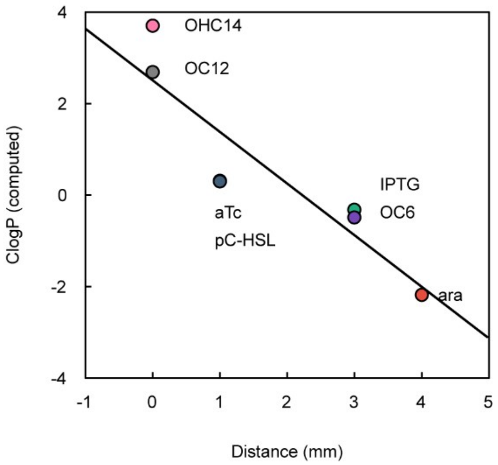

Appendix Figure S9: Inducer molecule ClogP analysis. (a) Chemical structures of the inducers tested. Annotated ClogP values were calculated using ChemDraw Professional (Perkin Elmer). **(b)** A plot of calculated ClogP as a function of distance traveled through the microplate for each inducer. The data were fit to a linear equation using Microsoft Excel; y = -1.1 x + 2.5; R^2 = 0.82.

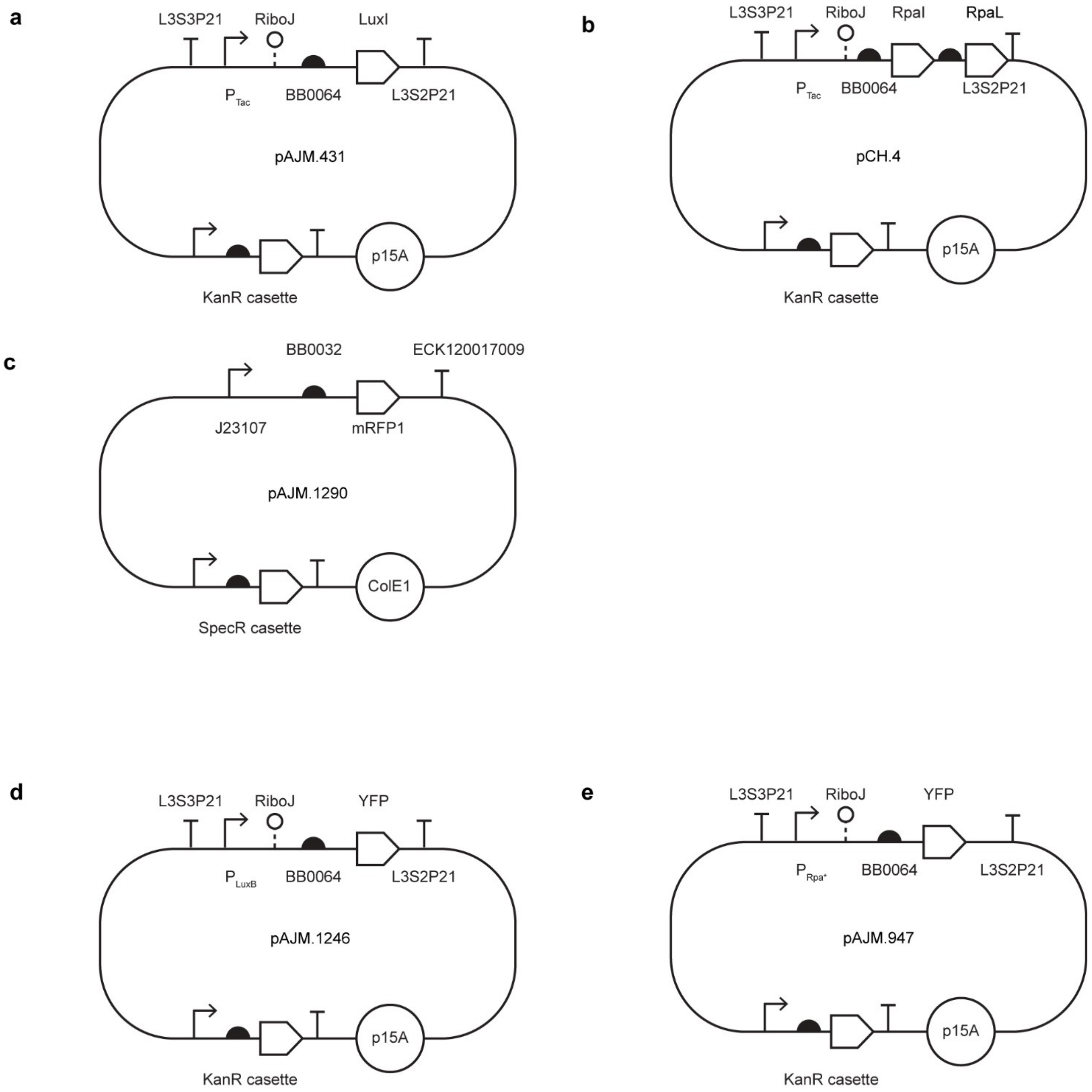

Appendix Figure S10: Quorum sender and receiver plasmid maps. (a) The OC6 quorum sender expresses the synthase *luxI* under control of the inducible promoter P_{Tac}. **(b)** The pC-HSL quorum sender expresses the synthase rpaI and the transporter rpaL under control of the inducible promoter P_{Tac}. **(c)** Sender strains were also transformed with a plasmid expressing red fluorescent protein (mRFP1) under control of the constitutive promoter J23107. **(d)** P_{LuxB} output strain plasmid. **(e)** P_{Rpa*} output plasmid. Sequences of individual parts can be found in Supplementary Table 1.

a

Carbenicillin

Distance	Rep 1	Rep 2	Rep 3	Rep 4	Rep 5	Rep 6	Rep 7	Rep 8	Rep 9	Student's T-Test
3.6	0.02	0.04	0.03	0.17	0.10	0.19	0.01	0.07	0.03	1.52E-06
7.2	0.00	0.01	0.00	0.08	0.09	0.17	0.04	0.04	0.06	2.43E-08
10.8	0.06	0.02	0.01	0.10	0.10	0.18	0.04	0.06	0.12	1.05E-06
14.4	0.41	0.89	0.69	0.97	1.03	0.79	0.63	0.67	0.33	1.35E-01

Kanamycin

Distance	Rep 1	Rep 2	Rep 3	Rep 4	Rep 5	Rep 6	Rep 7	Rep 8	Rep 9	
3.6	0.06	0.16	0.23	0.09	0.08	0.11	0.04	0.00	0.03	1.81E-06
7.2	0.04	0.05	0.04	0.16	0.31	0.27	0.01	0.09	0.04	8.62E-08
10.8	0.86	0.49	0.61	0.59	0.81	0.74	0.38	0.40	0.23	3.66E-03
14.4	1.46	0.98	1.00	0.71	1.04	0.95	0.75	0.49	0.50	8.33E-01

Tetracycline

Distance	Rep 1	Rep 2	Rep 3	Rep 4	Rep 5	Rep 6	Rep 7	Rep 8	Rep 9	
3.6	0.11	0.26	0.14	0.06	0.08	0.06	0.07	0.06	0.08	2.07E-06
7.2	0.66	0.74	0.77	0.70	0.51	0.42	0.32	0.68	0.68	3.24E-04
10.8	1.17	1.35	1.27	1.30	1.61	0.77	0.41	0.52	0.93	8.78E-01
14.4	1.30	1.18	1.26	1.25	1.13	0.84	0.58	1.08	0.90	2.48E-01

Control

Distance	Rep 1	Rep 2	Rep 3	Rep 4	Rep 5	Rep 6	Rep 7	Rep 8	Rep 9
3.6	1.38	1.71	2.45	1.10	1.13	0.93	0.84	1.09	1.15
7.2	1.05	1.90	1.22	0.95	0.95	0.84	0.95	1.32	1.18
10.8	0.94	1.68	1.34	0.49	0.99	0.50	1.27	1.17	1.22
14.4	0.44	1.17	1.24	0.70	1.12	0.59	0.87	1.20	0.83

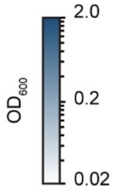

b

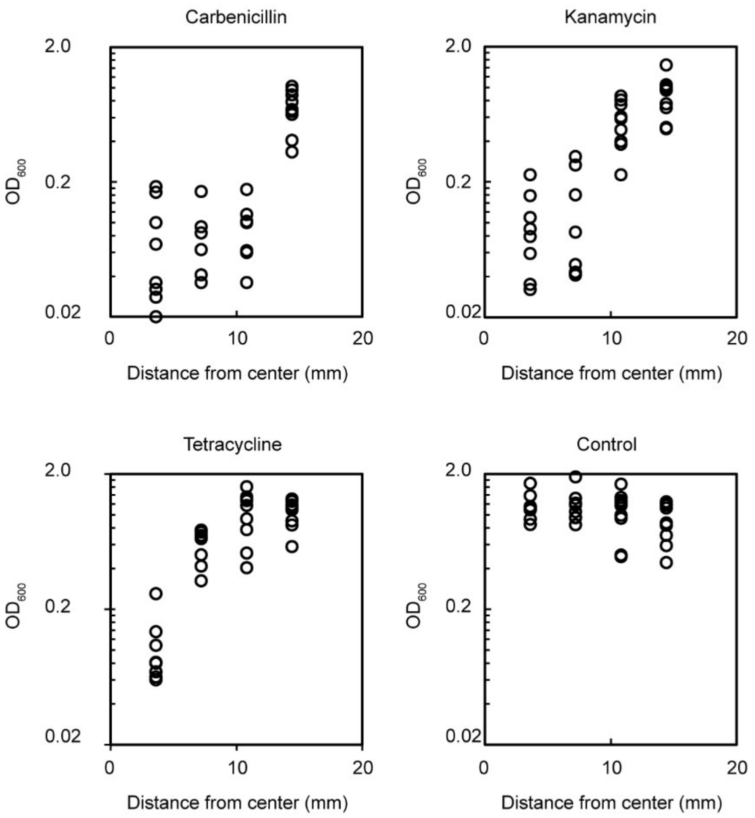

Appendix Figure S11: Antibiotic diffusion data. (a) Optical density reads from the antibiotic diffusion assay. Samples were collected from four distance from the center well. Three samples were collected per device, and three devices were run on different days, resulting in nine combined samples per condition. The control well contained no antibiotics in the center well. Data arrays from each tested microplate were compared to the data array from the control plate using an unpaired two-tailed Student's T-Test, and the values are noted in the figure. **(b)** A plot of optical density versus distance from the center of the center well for each condition.

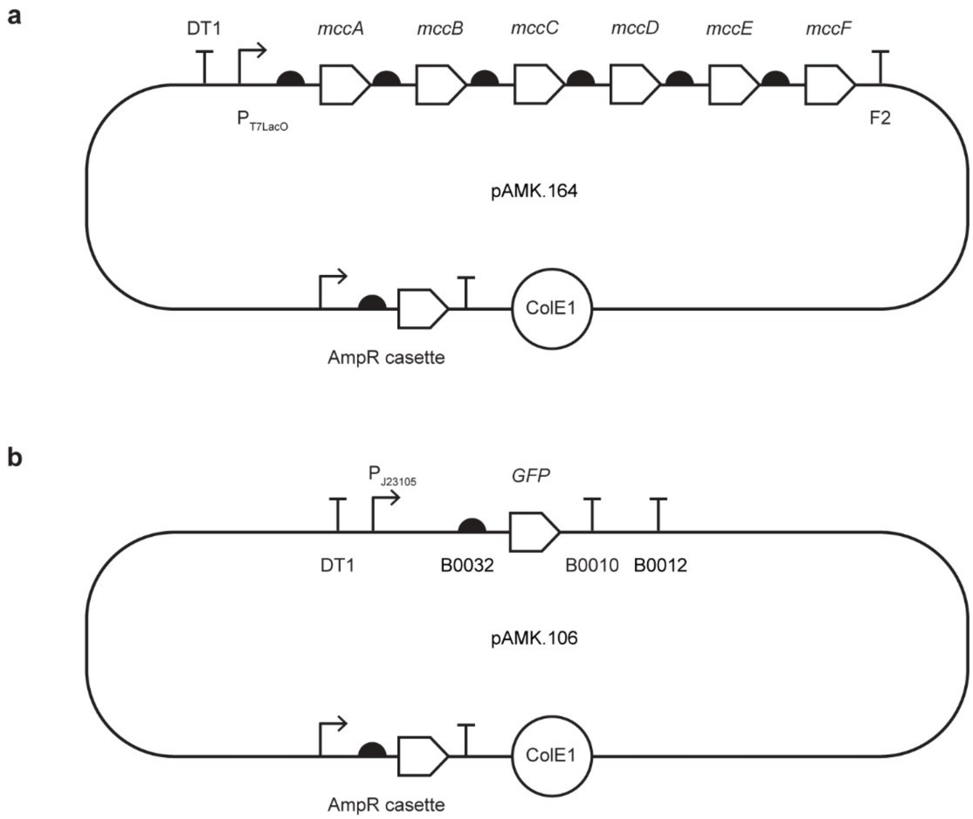

Appendix Figure S12: Microcin producer plasmid map. The *mcc7* cluster was cloned into a p15A plasmid under the control of the inducible promoter P_{T7LacO}. Sequences of individual parts can be found in Supplementary Table 1.

a

distance	Rep1	Rep2	Rep3	Rep4	Student's T-Test
3.6	0.05	0.06	0.04	0.06	0.02
7.2	0.14	0.16	0.13	0.12	0.75
10.8	0.15	0.12	0.12	0.09	0.97
14.1	0.17	0.17	0.10	0.09	0.69

OD_{600}: 0.02 – 0.2

Control

distance	Rep 1	Rep 2	Rep 3	Rep 4
3.6	0.13	0.20	0.10	0.09
7.2	0.16	0.16	0.10	0.09
10.8	0.13	0.16	0.12	0.07
14.1	0.18	0.21	0.11	0.08

b

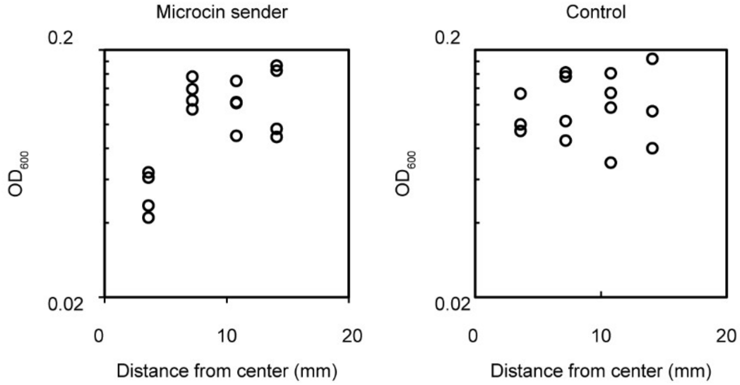

Appendix Figure S13: Microcin producer diffusion data. (a) Optical density reads from the microcin sender diffusion assay. Samples were collected from four distance from the center well. Four samples were collected per device, resulting in four total samples per condition. The control well contained no microcin sender in the center well. Data arrays from each tested microplate were compared to the data array from the control plate using an unpaired two-tailed Student's T-Test, and the values are noted in the figure. **(b)** A plot of optical density versus distance from the center of the center well for each condition.

**Appendix Table S1: Genetic Parts List**	
**Name**	**Sequence**
**Promoters**	
P_{Tac}	TGTTGACAATTAATCATCGGCTCGTATAATGTGTGGAATTGTGAGCGCTCACAATT
P_{Bad}	AGAAACCAATTGTCCATATTGCATCAGACATTGCCGTCACTGCGTCTTTTACTGGCTCTTCTCGCTAACCAAACCGGTAACCCCGCTTATTAAAAGCATTC<br>TGTAACAAAGCGGGACCAAAGCCATGACAAAAACGCGTAACAAAAGTGTCTATAATCACGGCAGAAAAGTCCACATTGATTATTTGCACGGCGTCACACTT<br>TGCTATGCCATAGCATTTTTATCCATAAGATTAGCGGATCCTACCTGACGCTTTTTATCGCAACTCTCTACTGTTTCTCCATACCCG
P_{Tet}	TCCCTATCAGTGATAGAGATTGACATCCCTATCAGTGATAGAGATACTGAGCAC
P_{Cin}	CCCTTTGTGCGTCCAAACGGACGCACGGCGCTCTAAAGCGGGTCGCGATCTTTCAGATTCGCTCCTCGCGCTTTCAGTCTTTGTTTTGGCGCATGTCGTTA<br>TCGCAAAACCGCTGCACACTTTTGCGCGACATGCTCTGATCCCCCTCATCTGGGGGGGCCTATCTGAGGGAATTTCCGATCCGGCTCGCCTGAACCATTCT<br>GCTTTCCACGAACTTGAAAACGCT
P_{rpa*}	ACCTGTCCGATCGGACAGTATTACGCAAGAAAATGGTTTGTTACTTTCGAATAAA
P_{Rpa*A}	ACCTGTCCGATCGGACAATATTACGCAAGAAAATGGTTTGTTACTTTCGAATAAA
P_{LuxB}	ACCTGTAGGATCGTACAGGTTTACGCAAGAAAATGGTTTGTTACAGTCGAATAAA
P_{LuxTA}	ACCTGTAGGATCTTACAAGTTTACGCAAGAAAATGGTTTGTTACTTTCGAATAAA
P_{LasB}	AACTAGCAAATGAGATAGATTTCGGTGAACCCGGACCCTTGCTAGGCTCGAAGAA
P_{J23100}	TTGACGGCTAGCTCAGTCCTAGGTACAGTGCTAGC
P_{LacIR}	GCGGCGCGCCATCGAATGGTGAAAAACCTTTCGCGGTATGGCATGATAGCGCCC
P_{J23107}	TTTACGGCTAGCTCAGCCCTAGGTATTATGCTAGC
P_{T7LacO}	TAATACGACTCACTATAGGGGAATTGTGAGCGGATAACAATT
**Ribosome Binding Sites**	
RBS_luxr1	GCGAAAGCAACTAAGGAGAATCTAT
RBS_cinr1	CCGCAGCACCTAAGGAGGCTAATTC
RBS_lasr1	TAAGGGCAGGTAAGGAGGTATTTTT
RBS_rpar1	TGGCAACAATAAGGAGGTAGTTTCT
RBS_lac1	GGAAGAGAGTCAATTCATGGAGGTGAAT
RBS_Tet2	TAATCAAGGTGGCAAAAA
RBS_ara2	TTCAAGGAGGTAAAT
RBS_e1	ATTAAAGAGGAGCGATTAAGC
RBS_rpai	TGGTTCCAAAGCCAGATACTAAGGAGGTCCCT
RBS_rpal	AAGGAGCGCTCTCCAGCCC
RBS_luxi	ATAAGAGCAAGATAATAAGGAACACTTCTA
RBS_mccA	ATTCCACTTGTGAATTAATCACAGTAATAGGAGGGTCGAC
RBS_mccB	CAAATGCATTCTGCGAGGTTTATTT
BB0064	AAAGAGGGGAAA
BB0032	TCACACAGGAAAG
**Ribozymes**	
RiboJ	AGCTGTCACCGGATGTGCTTTCCGGTCTGATGAGTCCGTGAGGACGAAACAGCCTCTACAAATAATTTTGTTTAA
**Genes**	
$luxR^{AM}$	ATGAAAAACATAAATGCCGACGACACATACAGAATAATTAATAAAATTAAAGCTTTTAGAAGCAATAATGATATTAATCAATGCTTATCTGATATGACTAA<br>AATGGTACATTGTGAATATTATTTACTCGCGATCATTTATCCTCATTCTATGGTTAAATCTGATATTTCAATCCTAGATAATTACCCTAAAAAATGGAGGC<br>AATATTATGATGACGCTAATTTAATAAAATATGATCCTATAGTAGATTATTCTAACTCCAATCATTCACCAATTAATTGGAATATATTTGAAAACAATGCT<br>GTAAATAAAAAATCTCCAAATGTAATTAAAGAAGCGAAAACATCAGGTCTTATCACTGGGTTTAGTTTCCCTATTCATATGGCTAACAATGGCTTCGGAAT<br>GCTTAGTTTTGCATATTCAGAAAAAGACAACTATATAGATAGTTTATTTTTACATGCGTGTATGAACATACCATTAATTGTTCCTTCTCTAGTTGATAATT<br>ATCGAAAAATAAATATAGCAAATAATAAATCAAACAACGATTTAACCAAAAGAGAAAAAGAATGTTTAGCGTGGGCATGCGAAGGAAAAAGCTCTTGGGAT<br>ATTTCAAAAATATTAGGATGCAGTGAGCGTACTGTCACTTTCCATTTAACCAATGCGCAAATGAAACTCAATACAACAAACCGCTGCCAAAGTATTTCTAA<br>AGCAATTTTAACAGGAGCAATTGATTGCCCATACTTTAAAAATTGATAA
$cinR^{AM2}$	ATGATTGAGAATACCTATAGCGAAAAGTTCGAGTCCGCGTTCGAACAGATCAAAGCGGCGGCCAACGTGGATGCCGCCATCCGTATTCTCCAGGCGGAATA<br>TAACCTCGATTTCGTCACCTACCATCTCGCCCAGACAATCGCGAGCAAGATCGATTCGCCCTTCGTGCGCACCACCTATCCGGATGCCTGGGTTTCCCGTT<br>ACCTCCTCAACTGCTATGTGAAGGTCGATCCGATCATCAAGCAGGGCTTCGAACGCCAGCTGCCCTTCGACTGGAGCGAGGTCGAACCGACGCCGGAGGCC<br>TATGCCATGCTGGTCGACGCCCAGAAACACGGCATCGATGACAATGGCTACTCCATCCCCGTCGCCGACAAGGCGCAGCGCCGCGCCCTGCTGTCGCTGAA<br>TGCCCATATACCGGCCGACGAATGGACCGAGCTCGTGCGCCGCTACCGCAACGAGTGGATCGAGATCGCCCATCTGATCCACCGCAAGGCCGTATATGAGC<br>TGCATGGCGAAAACGATCCGGTGCCGGCATTGTCGCCGCGCGAGATCGAGTGTCTGCACTGGACCGCCCTCGGCAAGGATTACAAGGATATTTCGGTCATC<br>CTGGGCATATCAGAGCATACCACACGCGATTACCTGAAAACCGCCCGCTTCAGGCTCGGCTGCACCACGATCTCGGCCGCCGCGTCGCGGGCTGTTCAATT<br>GTGCATCATCAATCCCTATAGGATCCGCATGACGCGACGTAATTGGTAATAG

lasR^{AM}	ATGGCCTTGGTTGACGGTTTTCTTGAGCTGGAACGCTCAAGTGGAAAATTGGAGTTGAGCGCCATCCTGCAGAAGATGGCGAGCGACCTCGGATTCTCGAA<br>GATCCTGTTCGGCCTGTTGCCTAAGGACAGCCAGGACTACGAGAACGCCTTCATCGTCAGCAACTACCCGGCCGCCTGGCGCGAGCATTACGACCGAACTG<br>GCTACGCGCGGGTCGACCCGACGGTCAGTCACTGTACCCAGAGCGTACTGCCGATTTTCTGGGAACCGCCCATCTACCAGACGCGAAAGCAGCACGAGTTC<br>TTCGAGGAAGCCTTGGCCGCTGGCCTGGTGTATGGACTGACCATGCCGCTGCATGGTGCTCGCGGCGAACTCGGCGCGCTGAGCCTCAGCGTGGAAGCGGA<br>AAACCGGGCCGAGGCCAACCGTTTCATGGAGTCGGTCCTGCCGACCCTGTGGATGCTCAAGGACTACGCACTGCAGAGCGGTGTCGGAATGGCCTTCGAAC<br>ATCCGATCAGCAAACCGGTGGTTCTGACCAGCCGAGAGAAGGAAGTGTTACAGTGGTGCGCCATCGGCAAGACCAGCTGGGAGATATCGGTTATCTGCAAC<br>TGCTCGGAAGCCAATGTGAACTTCCATATGGGAAATATTCGGCGGAAGTTCGGTGTGACCTCCCGCCGCGTAGCGGCCATTATGGCCGTTAATTTGGGTCT<br>TATTACTCTCTGATAA
rpaR^{AM}	ATGATTGTGGGTGAAGATCAGCTGTGGGGTCGTCGTACACTGGAATTTGTTGATAGCGTTGAACGTCTGGAAGCACCGGCACTGATTAGCCGTTTTGAAAG<br>CCTGATTGCAAGCTGTGGTTTTACCGCCTATATCATGGCAGGTCTGCCGAGCCGTAATGCCGGTCTGCCGGAACTGACCCTGGCAAATGGTTGGCCTCGTG<br>ATTGGTTTGATCTGTATGTTAGCGAAAACTTTAGCGCAGTTGATCCGGTTCCGCGTTATGGTGCAACCACTGTTCATCCGTTTGTTTGGAGTGATGCACCG<br>TATGATCGTGACCGTGATCAGGCAGCACATCGTGTTATGACCCGTGCAGCAGAATTTGGTCTGGTTGAAGGTTATTGTATTCCGCTGCATTACGATGATGG<br>TAGCGCAGCAATTAGTATGGCAGGTGAAGATCCTGATCTGAGTCCGGCAGCCCGTGGTGTAATGCAGCTGGTTAGCATTTATGCACATAGCCGTCTGCGTG<br>TACTGAGCCGTCCGAAACCGATTCGTCGTAATCGTCTGACACCGCGTGAATGTGAAATTCTGCAGTGGGCAGCACAGGGTAAAACCGCATGGGAAATTAGC<br>GTTATTCTGTGTATTACCGAACGCACCGTTAAATTTCATCTGATTGAAGCAGCACGTAAACTGGATGCAGCAAATCGTACCGCAGCAGTTGCAAAAGCACT<br>GACACTGGGTCTGATTCGTCTGTGATAA
lacI^{AM}	ATGAAACCAGTAACGTTATACGATGTCGCAGAGTATGCCGGTGTCTCTTATATGACCGTTTCCCGCGTGGTGAACCAGGCCAGCCACGTTTCTGCGAAAAC<br>GCGGGAAAAAGTGGAAGCGGCGATGGTGGAGCTGAATTACATTCCCAACCGCGTGGCACAACAACTGGCGGGCAAACAGTCGTTGCTGATTGGCGTTGCCA<br>CCTCCAGTCTGGCCCTGCACGCGCCGTCGCAAATTGTCGCGGCGATTAAATCTCGCGCCGATCAACTGGGTGCCAGCGTGGTGGTGTCGATGGTAGAACGA<br>AGCGGCGTCGAAGCCTGTAAAGCGGCGGTGCACAATCTTCTCGCGCAACGCGTCAGTGGGCTGATCATTAACTATCCGCTGGATGACCAGGATGCCATTGC<br>TGTGGAAGCTGCCTGCACTAATGTTCCGGCGTTATTTCTTGATGTCTCTGACCAGACACCCATCAACAGTATTATTTACTCCCATGAGGACGGTACGCGAC<br>TGGGCGTGGAGCATCTGGTCGCATTGGGTCACCAGCAAATCGCGCTGTTAGCGGGCCCATTAAGTTCTGTCTCGGCGCGTCTGCGTCTGGCTGGCTGGCAT<br>AAATATCTCACTCGCAATCAAATTCAGCCGATAGCGGAACGGGAAGGCGACTGGAGTGCCATGTCCGGTTTTCAACAAACCATGCAAATGCTGAATGAGGG<br>CATCGTTCCCACTGCGATGCTGGTTGCCAACGATCAGATGGCGCTGGGCGCAATGCGCGCCATTACCGAGTCCGGGCTGCGCGTTGGTGCGGATATCTCGG<br>TAGTGGGATACGACGATACCGAAGATAGCTCATGTTATATCCCGCCGTTAACCACCATCAAACAGGATTTTCGCCTGCTGGGGCAAACCAGCGTGGACCGC<br>TTGCTGCAACTCTCTCAGGGCCAGGCGGTGAAGGGCAATCAGCTGTTGCCAGTCTCACTGGTGAAAAGAAAAACCACCCTGGCGCCCAATACGCAAACCGC<br>CTCTCCCCGCGCGTTGGCCGATTCATTAATGCAGCTGGCACGACAGGTTTCCCGACTGGAAAGCGGGCAGTGA
tetR	ATGTCCAGATTAGATAAAAGTAAAGTGATTAACAGCGCATTAGAGCTGCTTAATGAGGTCGGAATCGAAGGTTTAACAACCCGTAAACTCGCCCAGAAGCT<br>AGGTGTAGAGCAGCCTACATTGTATTGGCATGTAAAAAATAAGCGGGCTTTGCTCGACGCCTTAGCCATTGAGATGTTAGATAGGCACCATACTCACTTTT<br>GCCCTTTAGAAGGGGAAAGCTGGCAAGATTTTTTACGTAATAACGCTAAAAGTTTTAGATGTGCTTTACTAAGTCATCGCGATGGAGCAAAAGTACATTTA<br>GGTACACGGCCTACAGAAAAACAGTATGAAACTCTCGAAAATCAATTAGCCTTTTTATGCCAACAAGGTTTTTCACTAGAGAATGCATTATATGCACTCAG<br>CGCTGTGGGGCATTTTACTTTAGGTTGCGTATTGGAAGATCAAGAGCATCAAGTCGCTAAAGAAGAAAGGGAAACACCTACTACTGATAGTATGCCGCCAT<br>TATTACGACAAGCTATCGAATTATTTGATCACCAAGGTGCAGAGCCAGCCTTCTTATTCGGCCTTGAATTGATCATATGCGGATTAGAAAAACAACTTAAA<br>TGTGAAAGTGGGTCCTAA
araC^{AM}	ATGGCTGAAGCGCAAAATGATCCCCTGCTGCCGGGATACTCGTTTAATGCCCATCTGGTGGCGGGTTTAACGCCGATTGAGGCCAACGGTTATCTCGATTT<br>TTTTATCGACCGACCGCTGGGAATGAAAGGTTATATTCTCAATCTCACCATTCGCGGTCAGGGGGTGGTGAAAAATCAGGGACGAGAATTTGTTTGCCGAC<br>CGGGTGATATTTTGCTGTTCCCGCCAGGAGAGATTCATCACTACGGTCGTCATCCGGAGGCTCGCGAATGGTATCACCAGTGGGTTTACTTTCGTCCGCGC<br>GCCTACTGGCATGAATGGCTTAACTGGCCGTCAATATTTGCCAATACGGGGTTCTTTCGCCCGGATGAAGCGCACCAGCCGCATTTCAGCGACTTTTTTGG<br>GCAAATCATTAACGCCGGGCAAGGGGAAGGGCGCTATTCGGAGCTGCTGGCGATAAATCTGCTTGAGCAATTGTTACTGCGGCGCATGCTAGCGATTAACG<br>GATCGCTCCATCCACCGATGGATAATCGGGTACGCGAGGCTTGTCAGTACATCAGCGATCACCTGGCAGACAGCAATTTTGATATCGCCAGCGTCGCACAG<br>CATGTTTGCTTGTCGCCGTCGCGTCTGTCACATCTTTTCCGCCAGCAGTTAGGGATTAGCGTCTTAAGCTGGCGCGAGGACCAACGTATCAGCCAGGCGAA<br>GCTGCTTTTGAGCACCACCCGGATGCCTATCGCCACCGTCGGTCGCAATGTTGGTTTTGACGATCAACTCTATTTCTCGCGGGTATTTAAAAAATGCACCG<br>GGGCCAGCCCGAGCGAGTTCCGTGCCGGTTTGGAAGAAAAAGTGAATGATGTAGCCGTCAAGTTGTCATGA
araE	ATGGTTACTATCAATACGGAATCTGCTTTAACGCCACGTTCTTTGCGGGATACGCGGCGTATGAATATGTTTGTTTCGGTAGCTGCTGCGGTCGCAGGATT<br>GTTATTTGGTCTTGATATCGGCGTAATCGCCGGAGCGTTGCCGTTCATTACCGATCACTTTGTGCTGACCAGTCGTTTGCAGGAATGGGTGGTTAGTAGCA<br>TGATGCTCGGTGCAGCAATTGGTGCGCTGTTTAATGGTTGGCTGTCGTTCCGCCTGGGGCGTAAATACAGCCTGATGGCGGGGGCCATCCTGTTTGTACTC<br>GGTTCTATAGGGTCCGCTTTTGCGACCAGCGTAGAGATGTTAATCGCCGCTCGTGTGGTGCTGGGCATTGCTGTCGGGATCGCGTCTTACACCGCTCCTCT<br>GTATCTTTCTGAAATGGCAAGTGAAAACGTTCGCGGTAAGATGATCAGTATGTACCAGTTGATGGTCACACTCGGCATCGTGCTGGCGTTTTTATCCGATA<br>CAGCGTTCAGTTATAGCGGTAACTGGCGCGCAATGTTGGGGGTTCTTGCTTTACCAGCAGTTCTGCTGATTATTCTGGTAGTATTTCTGCCAAATAGCCCG<br>CGCTGGCTGGCGGAAAAGGGGCGTCATATTGAGGCGGAAGAAGTATTGCGTATGCTGCGCGATACGTCGGAAAAAGCGCGAGAAGAACTCAACGAAATTCG<br>TGAAAGCCTGAAGTTAAAACAGGGCGGTTGGGCACTGTTTAAGATCAACCGTAACGTCCGTCGTGCTGTGTTTCTCGGTATGTTGTTGCAGGCGATGCAGC<br>AGTTTACCGGTATGAACATCATCATGTACTACGCGCCGCGTATCTTCAAAATGGCGGGCTTTACGACCACAGAACAACAGATGATTGCGACTCTGGTCGTA<br>GGGCTGACCTTTATGTTCGCCACCTTTATTGCGGTGTTTACGGTAGATAAAGCAGGGCGTAAACCGGCTCTGAAAATTGGTTTCAGCGTGATGGCGTTAGG<br>CACTCTGGTGCTGGGCTATTGCCTGATGCAGTTTGATAACGGTACGGCTTCCAGTGGCTTGTCCTGGCTCTCTGTTGGCATGACGATGATGTGTATTGCCG<br>GTTATGCGATGAGCGCCGCGCCAGTGGTGTGGATCCTGTGCTCTGAAATTCAGCCGCTGAAATGCCGCGATTTCGGTATTACCTGTTCGACCACCACGAAC<br>TGGGTGTCGAATATGATTATCGGCGCGACCTTCCTGACACTGCTTGATAGCATTGGCGCTGCCGGTACGTTCTGGCTCTACACTGCGCTGAACATTGCGTT<br>TGTGGGCATTACTTTCTGGCTCATTCCGGAAACCAAAAATGTCACGCTGGAACATATCGAACGCAAACTGATGGCAGGCGAGAAGTTGAGAAATATCGGCG<br>TCTGA
luxI	ATGACTATAATGATAAAAAAATCGGATTTTTTGGCAATTCCATCGGAGGAGTATAAAGGTATTCTAAGTCTTCGTTATCAAGTGTTTAAGCAAAGACTTGA<br>GTGGGACTTAGTTGTAGAAAATAACCTTGAATCAGATGAGTATGATAACTCAAATGCAGAATATATTTATGCTTGTGATGATACTGAAAATGTAAGTGGAT<br>GCTGGCGTTTATTACCTACAACAGGTGATTATATGCTGAAAAGTGTTTTTCCTGAATTGCTTGGTCAACAGAGTGCTCCCAAAGATCCTAATATAGTCGAA<br>TTAAGTCGTTTTGCTGTAGGTAAAAATAGCTCAAAGATAAATAACTCTGCTAGTGAAATTACAATGAAACTATTTGAAGCTATATATAAACACGCTGTTAG<br>TCAAGGTATTACAGAATATGTAACAGTAACATCAACAGCAATAGAGCGATTTTTAAAGCGTATTAAAGTTCCTTGTCATCGTATTGGAGACAAAGAAATTC<br>ATGTATTAGGTGATACTAAATCGGTTGTATTGTCTATGCCTATTAATGAACAGTTTAAAAAAGCAGTCTTAAATTAA
rpaI	ATGCAGGTTCATGTTATTCGTCGTGAAAATCGTGCACTGTATGCAGGTCTGCTGGAAAAATACTTTCGTATTCGCCATCAGATCTATGTTGTTGAACGTGG<br>TTGGAAAGAACTGGATCGTCCGGATGGTCGTGAAATTGATCAGTTTGATACCGAAGATGCAGTTTATCTGCTGGGTGTTGATAACGATGATATTGTTGCAG<br>GTATGCGTATGGTTCCGACCACCAGTCCGACCCTGCTGAGTGATGTTTTTCCGCAGCTGGCACTGGCAGGTCCGGTTCGTCGTCCTGATGCATATGAACTG<br>AGCCGTATTTTTGTTGTTCCGCGTAAACGTGGTGAACATGGTGGTCCGCGTGCAGAAGCAGTTATTCAGGCAGCAGCAATGGAATATGGTCTGAGCATTGG<br>TCTGAGTGCATTTACCATTGTTCTGGAAACCTGGTGGCTGCCTCGTCTGGTTGATCAGGGCTGGAAAGCAAAACCGCTGGGTCTGCCGCAGGATATTAATG<br>GTTTTAGCACCACCGCAGTTATTGTGGATGTTGATGATGATGCATGGGTGGGTATTTGTAATCGTCGTAGCGTTCCGGGTCCGACACTGGAATGGCGTGGT<br>CTGGAAGCAATTCGTCGTCATAGCCTGCCGGAATTTCAGGTTATTAGCTAA
rpaL	ATGGAGAAGGACACGAAACAAGTCGACATCATTTTTCGTTCGAAGCTCCCCGACATTTACATCCCCAACCATCTTCCGCTTCACTCGTACTGTTTTGAAAA<br>CATTTCCGAATTCAGCAGCCGGCCCTGTCTCATTAACGGCGCAAATAAGCAGATTTACACCTACGCGGATGTTGAACTTAATTCGCGCAAAGTCGCCGCCG<br>GCCTCCATAAGCAGGGTATCCAGCCCAAAGATACGATCATGATCCTGCTTCCCAACTCGCCCGAATTCGTTTTCGCGTTTATTGGCGCGTCGTACCTGGGC<br>GCAATCAGCACGATGGCCAACCCCCTTTTTACCCCCGCCGAGGTGGTTAAGCAAGCCAAGGCGAGCTCCGCCAAGATTATTGTCACGCAGGCATGCCACGT<br>CAATAAAGTTAAGGATTACGCATTTGAAAATGATGTGAAGATTATCTGCATCGACAGCGCCCCCGAGGGCTGCCTGCACTTCTCGGTCCTTACGCAGGCCA<br>ATGAGCACGACATTCCCGAGGTGGAGATTCAGCCCGATGACGTCGTCGCGCTCCCCTATAGCAGCGGCACCACGGGTCTGCCCAAAGGTGTCATGCTCACC<br>CACAAAGGCCTCGTGACCAGCGTGGCACAACAGGTGGACGGCGAAAACCCCAACCTTTACATCCATTCCGAGGACGTTATGCTGTGTGTGCTCCCCCTGTT<br>TCACATTTACTCCCTTAACTCGGTCCTTCTTTGCGGCCTCCGTGTTGGCGCAGCAATCCTTATTATGCAAAAGTTCGATATTGTCAGCTTCCTGGAGCTCA<br>TTCAACGCTACAAAGTCACGATTGGCCCCTTCGTCCCCCCGATTGTGCTCGCGATTGCGAAGTCCCCCATGGTGGATGACTACGATCTCAGCAGCGTCCGT<br>ACGGTCATGTCCGGCGCGGCACCGCTCGGTAAAGAACTGGAGGATACGGTCCGGGCCAAATTCCCGAATGCGAAGCTGGGTCAGGGTTACGGCATGACCGA<br>GGCAGGTCCCGTGCTCGCCATGTGCCTGGCATTCGCGAAGGAGCCCTTCGAGATCAAGAGCGGTGCGTGCGGCACGGTCGTTCGGAACGCGGAAATGAAAA<br>TCGTGGACCCCAAAACCGGCAATTCCCTTCCCCGCAACCAATCCGGCGAAATTTGCATCCGGGGTGACCAGATTATGAAGGGCTATCTTAATGACCCGGAA<br>GCCACGGCGCGGACGATTGACAAAGAAGGTTGGCTGTATACCGGTGACATTGGTTACATCGACGATGACGACGAGCTTTTTATTGTGGACCGGCTCAAAGA<br>ACTCATCAAATATAAAGGCTTTCAAGTTGCGCCCGCGGAGCTGGAGGCACTGCTTCTTAACCATCCCAACATCTCGGATGCCGCGGTCGTGCCGATGAAGG<br>ATGAACAAGCGGGTGAAGTGCCGGTCGCGTTTGTGGTTCGGAGCAACGGCTCCACGATTACGGAGGATGAGGTTAAGGATTTTATTTCCAAGCAGGTTATC<br>TTTTATAAACGGATTAAACGCGTTTTCTTCGTCGATGCCATCCCCAAATCCCCCAGCGGCAAGATCCTTCGGAAAGATCTTCGTGCGAAACTGGCAGCAGG<br>TCTTCCGAATTAG
mccA	ATGCGTACTGGTAATGCAAACTA
mccBCDEF	ATGGATTATATATTGGGTCGCTATGTCAAAATTGCTCGATACGGGAGCGGTGGATTAGTTGGTGGAGGCGGAAAGGAGCAATATGTTGAGGATTTAGCGCT<br>TTGGGAAAATATTATAAAAACCGCATATTGTTTCATAACCCCATCGAGTTATACTGCAGCGTTGGAAACTGTCAATATACCTGAAAAAGATTTCTCTAATT<br>GCTTTAGGTTCCTTAAGGAAAATTTCTTTATTATACCTAGTGAGTATAATAACTCTACTGAGAATAATCGTTACTCTCGTAACTTTTTACATTATCAAAGT<br>TATGGTGCTAACCCTGTTCTTGTGCAAGATAAACTAAAAGATGCTAAGGTAGTAATTCTTGGTTGTGGGGGTATAGGAAATCATGTATCTGTTATACTCGC

	AACGTCTGGTATTGGCGAAATTATACTAATTGATAATGATCAAATAGAAAATACAAATTTGACAAGGCAGGTTTTATTTTCAGAGAATGATGTCGGTAAAA<br>ATAAAACAGAAGTTATAAAACGCGAGTTATTAAAAAGAAACTCTGAAATTTCGGTGTCTGAAATCGCTTTGAATATAAACGATTACACTGATCTTCATAAG<br>GTACCAGAAGCTGATATATGGGTTGTTTCTGCAGATCATCCATTTAATCTGATCAACTGGGTTAATAAATATTGCGTTAGAGCAAATCAGCCCTATATAAA<br>TGCTGGGTATGTTAATGATATTGCAGTCTTTGGTCCATTATATGTTCCTGGTAAAACAGGATGTTATGAGTGCCAAAAAGTCGTGGCAGATCTATATGGTT<br>CAGAAAAGGAAAACATTGATCATAAGATTAAGCTAATCAATAGCAGATTTAAACCAGCAACATTTGCACCTGTGAATAATGTTGCCGCTGCATTATGTGCG<br>GCAGACGTAATTAAGTTCATTGGAAAATACTCCGAGCCATTATCACTTAATAAAAGAATAGGCATCTGGTCAGATGAAATAAAAATACATTCCCAAAATAT<br>GGGCCGTTCGCCTGTTTGCTCTGTGTGTGGAAATAGAATGTGAATACTGCAAATATTTTATCTGTGAACTATGGAGCTTATAATGCTATACGTATGTTAAT<br>TGGTGTCTACCATGCAGCATTTCTTATATCTACAGGTGTCACTATATCTCAACTTGCTGTTTTACAAATCGTTTTTTCTGTGACAGTGCTGATTCTTGATT<br>TTCCTTTATCTGTCATTGCAGACAGATTTTACAGGAAACAGGTTGTAGTTAGTGGCGTATTTTTTACTATTTTATTTTACCCTCTTTGTATTCAGTCTCCT<br>GATATGCTGGCATTGGTTTTTTCTGAAATATTTTATGCCATTGGTATTTGTTGTATATCTGGGGCTATAGAGGGATGGGTGTTAAGTGCTCTGGATGGTAG<br>AAATGAAAAATTTACCTTATGTGCTCATTTATGCGAAAGGGTATCTTCCTTTGGGAGTGTGATAACTGGTGCTGTTGGAATATTAGTTGTCTACTTCTATG<br>ATTCTTATAAATCTGGCTATATAGTATCGTTCTTCTTGATGTTTGTTGTCTTTATCGTTTTTTTATGTGTAAAAACTAACGGCAGTGGGGGTGAAAAAGAA<br>GCACGAGATAGTATTATTAAACAAGCAAAAGAGAGTATTGGATTGATAACAGGAAGTTATAATGGGACATATTTTTTATCTATAACATGTATGTTTACACT<br>TGGTATACAGGTAATTTATCATTTTTGGCAGCCAATTATGTTATCTGGGGAAAGTATTGGCTCATTATCAAAAAGAGAGCTTATAACTTTACTGATGTGTC<br>ATATTGGTGCATTTTCAATGCAATATATAGCAAACTATTTTATGCCAAAGATAGATGTAAGATGTAAACTTTATATACTTGTTTGTACATTATGTTCATTT<br>CTTAGTTCAATCGCCTGTATTTTACTATTTATGTTAATAACAGATAATAATGTTGTTCTCGGTGTAGCTATTTTCTCTATAATCCATGGGATGACTTCAAC<br>AATACCTGTTGGAGCACAGAATATATTCATAAGCAGACTTAATGGCAATTATAAGAATATTATTTCGGGAGCTCTTGGTCTTGTTTCTCTTTCATGTCGTG<br>TTGCTTCCATTTCTGTGTTGGGAGTTATATCTGTATTACCAGGTAATATGCCTGTCACCACTTATATATTTATCTCTGCAATTGCATGTGTACTGTGTGGT<br>TGTTTGTTCTTAATGTGGTCCTTTAAAAAGGAGATGGTATGACGATTAAGCATGCTGAAGAATTGACGGAAGAAGTCATTTTAAATATGAATTACCCTGAT<br>TTTGTTGCTCTGATGCAACAGGATAACACTCCTCCTGGAGCAGAGTACACCATAGACTACTGGATTAAGCATGGATGTATTAACAAAAAGTCTCACCTATT<br>AGATTTAGCATGTTCCACGGGTTTTAGTTCACGTGAATGCTTCAAAAAAGAAGGTGCATCTGCAGAGGGTATTGATATATCTGAATCAGCTGTAATGGTCG<br>CAAATGAAAAGGCAAAAAAGCTTAAGGCTAATAATTTATTGAAATATTATGTAGCTGATGCTTGTGATTTACCATTTGAAGATAACACTTTTACACATGTT<br>CTCGGTGGATGTAATTTTGCATTTATACAAAATCGGTTAATAGCCTTGAATGAAACTCATCGATGTTTAAACCACATGGAAGCATGTGCATTTCAAATTT<br>TTATTACAGGCGTAAAATATCAGATAAACTTATCAATGATGTATATAATGCAATAAATTTCAGACCCAATCCTTTGTGGACTTTGGAATACTGGCATCAGT<br>TTTTTTCTGAAAGATTTACTCTGGTTTCAGAAGAAAATCATGAGATGGAATCTCAAAGTGAAGATGAGTTAAAAGCCGATATTTATGATTACATTTTCAAT<br>AGAAATGAGTTTACAAAAGGTTTGAATGGTTCACTTCAGAATGTATTTTTTGAAAGATTTTTAAAAATACGCAGACCATTAAATATCCAAAGAGATTATCA<br>GGGAGTTACATTGCAAATATGGCGCAAAAAATAACACCATCTAAAATTGTTAAACATGCCCGGGAGTTAATTATTAAGGGGATTGAATCGGGCGATAACTC<br>ATTTGTCATTTTTGATGTAGATGGAGCTCTGGAGCGTCTGGAACATTACAGGTGTCAACTTAAGAGTTTTTTCCCCAACAGCAGTATCGCATATTCATATA<br>AGTCAAATAATCTGGCACAATGGTGCCAGATTATCTCAGGTAAAGGTTTGTATGCAGAGGTGTGCTCTGTTGATGAAATGAACCTCGCTAAAAGAGACGGT<br>TTTAACCGGATTGTTTTTGATGGCCCTTTAAAAAAAACGAGTGAACTATTAAAGGCAATAGAGATTGGGGCATTAATTGAAGTTGACAATATTGATGAATG<br>TAAAAGACTTAATGAACTATGTAAATTGCATAAGTTGACATGTAGAATTCATTTGAGATTATCGCATTACTATGATGATAATTTATCAAGATTTGGCTTAT<br>CTGAAAGTGAGGCTATTAATTTACTGGAGATGTTGATCAGTAAGTCAGAGTATCTCATTTTAGACGGGTTCCATTTACATGTTGGCTCTAATCTACCCAAT<br>GCGGAAAAAATATGTAAAGCTGTTATACAGTATCATGAGTTGATTCTTAGGTATATGCCAGATGATGGCACTCTTAATCTTGGTAGTGGGATACCTGCAGA<br>TTCTTTCTCAGCATCCAGTGATAACCCAACTCCATGTCCGGAGGTATTTTTCTCATCGATATATGATACAATAAAAAATTGCTTCGGTACTGTATGTGATA<br>AATGGAACTATATTTTTGAACCTGGGAGACATCTGGTCGAAGACTTTGGCTACTTTATAGGGAAAGTTATTAGTACTAAAAACAGGTATGGTGTTAAAGTT<br>GCTCAAACTAACATTGGTATAAACTGGATACCTTCGATCAGAAATTGGGACCATTCATTTACATTGTTTCATAATCATAACCATATTTCAGATGATAAATC<br>TGATGAGTATATTATTGCTGGATTCAATTGCTTTGAATGTGATTGCTTGTTTCCTTCAGTTATTCTTCCCAGTAACTTATCTGATTATTTATTTTCTGTTC<br>GAGGGTGTGGTGCTTATGATATGCAAACAGGAAACCAGTGGACGCGTAATCTTTATGCTGTATATACCATAACAAATGATGTTGTTAATATATCAAGGATT<br>CACAGAAGAGAACTTGATTTCCGGAAGTATGATGTATCTCTTACGCCATCAGGAATAAAAGTAAATGATGAGATTACATTATTGTATCCAGCGTTAAAATA<br>CGCTGAAGAGTTATATTTGCTCATTAATCAAAACAAAATAAATTTCATTAAAAGCATGGCATGGCCAGCGTTTGTTAATAACATTTCTGATTCTGTTTCTT<br>TCATTGAGCAATCAATGATTGATAACCAGAATGAAAAAGCATTAATCCTCTTTATTAAATACAAAACTAAGATAGCTGGCGTTGTATCTTTTAATATTATT<br>GACCATGCCAATAAAACAGCATATATTGGCTATTGGTTAGGCGCTAACTTTCAGGGAAAGGGGATTGTAACCAATGCTATAAATAAACTGATACAGGAGTA<br>TGGGGATTCGGGCGTTATAAAAAGATTTGTTATAAAATGTATTGTTGATAATAAAAAAAGTAATGCCACGGCATTGAGGTGTGGCTTCACCTTAGAGGGTG<br>TTCTGCAAAAAGCAGAAATACTCAACGGTGTATCATACGATCAAAATATTTATTCGAAAGTAATTGGTTAATGATGGTTCATTTTGACACTAAATGGAAAG<br>GAAAAATCCTCATGATGATACAATCTCATCCGCTACTGGCCGCTCCCCTGGCAGTAGGAGATACAATTGGTTTCTTTTCATCATCTGCTCCGGCAACAGTT<br>ACTGCAAAAAATCGTTTTTTTCGGGGAGTTGAGTTTCTTCAGAGAAAGGGATTTAAGCTGGTATCAGGGAAGCTTACCGGTAAAACAGATTTTTATCGTTC<br>AGGTACTATTAAAGAAAGAGCTCAAGAATTTAATGAGTTAGTCTACAATCCTGATATTACCTGTATAATGTCAACGATCGGTGGAGATAACAGTAATTCAC<br>TACTACCGTTTCTGGACTATGATGCTATCATTGCAAACCCCAAAATTATCATAGGTTACTCAGATACAACTGCTTTATTAGCAGGAATATATGCAAAAACA<br>GGGTTAATAACATTCTATGGGCCAGCTCTTATTCCTTCGTTTGGTGAGCATCCACCTCTTGTGGATATAACATATGAATCATTTATTAAAATACTAACAAG<br>AAAACAATCAGGAATATATACCTACACATTACCTGAAAAGTGGAGTGATGAGAGCATAAACTGGAATGAAAACAAGATATTAAGGCCTAAGAAACTATATA<br>AAAACAACTGTGCCTTTTATGGTTCCGGAAAAGTTGAGGGGCGTGTAATTGGAGGAAATCTAAATACTTTGACAGGTATATGGGGGAGTGAATGGATGCCT<br>GAAATTCGGAATGGAGATATATTGTTTATTGAGGACAGTCGGAAAAGCATTGCAACAGTTGAACGATTATTCTCTATGCTAAAGCTTAATCGCGTGTTTGA<br>TAAAGTTAGTGCAATAATACTCGGGAAACATGAGCTTTTTGATTGTGCAGGAAGTAAACGCAGACCATATGAAGTATTAACAGAGGTATTAGATGGGAAAC<br>AAATTCCTGTACTGGATGGATTTGATTGTTCACATACACATCCAATGCTAACTCTTCCACTTGGTGTAAAATTAGCTATTGACTTTGACAACAAAAATATA<br>TCTATAACAGAACAATATCTATCTACCGAGAAATAA
mRFP1	ATGGCTTCCTCCGAAGACGTTATCAAAGAGTTCATGCGTTTCAAAGTTCGTATGGAAGGTTCCGTTAACGGTCACGAGTTCGAAATCGAAGGTGAAGGTGA<br>AGGTCGTCCGTACGAAGGTACCCAGACCGCTAAACTGAAAGTTACCAAAGGTGGTCCGCTGCCGTTCGCTTGGGACATCCTGTCCCCGCAGTTCCAGTACG<br>GTTCCAAAGCTTACGTTAAACACCCGGCTGACATCCCGGACTACCTGAAACTGTCCTTCCCGGAAGGTTTCAAATGGGAACGTGTTATGAACTTCGAAGAC<br>GGTGGTGTTGTTACCGTTACCCAGGACTCCTCCCTGCAAGACGGTGAGTTCATCTACAAAGTTAAACTGCGTGGTACCAACTTCCCGTCCGACGGTCCGGT<br>TATGCAGAAAAAAACCATGGGTTGGGAAGCTTCCACCGAACGTATGTACCCGGAAGACGGTGCTCTGAAAGGTGAAATCAAAATGCGTCTGAAACTGAAAG<br>ACGGTGGTCACTACGACGCTGAAGTTAAAACCACCTACATGGCTAAAAAACCGGTTCAGCTGCCGGGTGCTTACAAAACCGACATCAAACTGGACATCACC<br>TCCCACAACGAAGACTACACCATCGTTGAACAGTACGAACGTGCTGAAGGTCGTCACTCCACCGGTGCTTAA
YFP	ATGGTGAGCAAGGGCGAGGAGCTGTTCACCGGGGTGGTGCCCATCCTGGTCGAGCTGGACGGCGACGTAAACGGCCACAAGTTCAGCGTGTCCGGCGAGGG<br>CGAGGGCGATGCCACCTACGGCAAGCTGACCCTGAAGTTCATCTGCACCACAGGCAAGCTGCCCGTGCCCTGGCCCACCCTCGTGACCACCTTCGGCTACG<br>GCCTGCAATGCTTCGCCCGCTACCCCGACCACATGAAGCTGCACGACTTCTTCAAGTCCGCCATGCCCGAAGGCTACGTCCAGGAGCGCACCATCTTCTTC<br>AAGGACGACGGCAACTACAAGACCCGCGCCGAGGTGAAGTTCGAGGGCGACACCCTGGTGAACCGCATCGAGCTGAAGGGCATCGACTTCAAGGAGGACGG<br>CAACATCCTGGGGCACAAGCTGGAGTACAACTACAACAGCCACAACGTCTATATCATGGCCGACAAGCAGAAGAACGGCATCAAGGTGAACTTCAAGATCC<br>GCCACAACATCGAGGACGGCAGCGTGCAGCTCGCCGACCACTACCAGCAGAACACCCCAATCGGCGACGGCCCCGTGCTGCTGCCCGACAACCACTACCTT<br>AGCTACCAGTCCGCCCTGAGCAAAGACCCCAACGAGAAGCGCGATCACATGGTCCTGCTGGAGTTCGTGACCGCCGCCGGGATCACTCTCGGCATGGACGA<br>GCTGTACAAGTAA
**Terminators**	
DT60	ACATTTAATAAAAAAAGGGCGGTCGCAAGATCGCCCTTTTTTACGTATGACACCTCGTCTCAACCAAAGCAATCAACCAGTGAAAAATGGCGCCCATCGGC<br>GCCATTTTTTTATGGTTCCTTATCATCTGGCGAATCGGA
DT11	AACGCATGAGAAAGCCCCCGGAAGATCACCTTCCGGGGGCTTTTTTATTGCGCTCCTTGGCCCTCCATCCTTAGATAGTTCAGCCAAAAACTTAAGACCG<br>CCGGTCTTGTCCACTACCTTGCAGTAATGCGGTGGACAGGATCGGCGGTTTTCTTTTCTCTTCTCAATCATAGGCAATACGATCGCATGTCC
F2	AAAAAAAAACCCCGCCCCTGACAGGGCGGGGTTTTTTTTT
IOT	TAATTGGTAACGAATCAGACAATTGACGGCTCGAGGGAGTAGCATAGGGTTTGCAGAATCCCTGCTTCGTCCATTTGACAGGCACATTATGCATCGATGAT<br>AAGCTGTCAAACATGAGCAGATCCTCTACGCCGGACGCATCGTGGCCGGCATCACCGGCGCCACAGGTGCGGTTGCTGGCGCCTATATCGCCGACATCACC<br>GATGGGGAAGATCGGGCTCGCCACTTCGGGCTCATGAGCAAATATTTTATCTG
ECK120017009	GATCTAACTAAAAAGGCCGCTCTGCGGCCTTTTTTCTTTTCACT
DT54NSNS	GGAAACACAGAAAAAAGCCCGCACCTGACAGTGCGGGCTTTTTTTTTCGACCAAAGGCTCGGTACCAAATTCCAGAAAAGACACCCGAAAGGGTGTTTTTT<br>CGTTTTGGTCC
L3S3P21	CCAATTATTGAAGGCCTCCCTAACGGGGGGCCTTTTTTTGTTTCTGGTCTCCC
L3S2P21	CTCGGTACCAAATTCCAGAAAAGAGGCCTCCCGAAAGGGGGGCCTTTTTTCGTTTTGGTCC

Appendix Table S2: Quorum sensor activator proteins		
**Protein**	**Amino acid sequence**	**Source**
CinR	MIENTYSEKFESAFEQIKAAANVDAAIRILQAEYNLDFVTYHLAQTIASKIDSPFVRTTYPDAWVSRYLLNSYVKVDPIV<br>KQGFERQLPFDWSEVEPTPEAYAMLVDAQKHGIGGNGYSIPVADKAQRRALLSLNARIPADEWTELVRRCRNEWIEIAHL<br>IHRKAVYELHGENDPVPALSPREIECLHWTALGKDYKDISVILGISEHTTRDYLKTARFKLGCATISAAASRAVQLRIIN<br>P	iGEM C0077 (Chen *et al*, 2015; Lithgow *et al*, 2000)
CinR^{AM2}	MIENTYSEKFESAFEQIKAAANVDAAIRILQAEYNLDFVTYHLAQTIASKIDSPFVRTTYPDAWVSRYLLNCYVKVDPII<br>KQGFERQLPFDWSEVEPTPEAYAMLVDAQKHGIDDNGYSIPVADKAQRRALLSLNAHIPADEWTELVRRYRNEWIEIAHL<br>IHRKAVYELHGENDPVPALSPREIECLHWTALGKDYKDISVILGISEHTTRDYLKTARFRLGCTTISAAASRAVQLCIIN<br>PYRIRMTRRNW	Evolved (This work)
LasR	MALVDGFLELERSSGKLEWSAILQKMASDLGFSKILFGLLPKDSQDYENAFIVGNYPAAWREHYDRAGYARVDPTVSHCT<br>QSVLPIFWEPSIYQTRKQHEFFEEASAAGLVYGLTMPLHGARGELGALSLSVEAENRAEANRFMESVLPTLWMLKDYALQ<br>SGAGLAFEHPVSKPVVLTSREKEVLQWCAIGKTSWEISVICNCSEANVNFHMGNIRRKFGVTSRRVAAIMAVNLGLITL	(Tamsir *et al*, 2011)
LasR^{AM}	MKNINADDTYRIINKIKAFRSNNDINQCLSDMTKMVHCEYYLLAIIYPHSMVKSDISILDNYPKKWRQYYDDANLIKYDP<br>IVDYSNSNHSPINWNIFENNAVNKKSPNVIKEAKTSGLITGFSFPIHMANNGFGMLSFAYSEKDNYIDSLFLHACMNIPL<br>IVPSLVDNYRKINIANNKSNNDLTKREKECLAWACEGKSSWDISKILGCSERTVTFHLTNAQMKLNTTNRCQSISKAILT<br>GAIDCPYFKN	Evolved (This work)
LuxR	MKNINADDTYRIINKIKACRSNNDINQCLSDMTKMVHCEYYLLAIIYPHSMVKSDISILDNYPKKWRQYYDDANLIKYDP<br>IVDYSNSNHSPINWNIFENNAVNKKSPNVIKEAKTSGLITGFSFPIHTANNGFGMLSFAHSEKDNYIDSLFLHACMNIPL<br>IVPSLVDNYRKINIANNKSNNDLTKREKECLAWACEGKSSWDISKILGCSERTVTFHLTNAQMKLNTTNRCQSISKAILT<br>GAIDCPYFKN	(Moon *et al*, 2012)
LuxR^{AM}	MKNINADDTYRIINKIKAFRSNNDINQCLSDMTKMVHCEYYLLAIIYPHSMVKSDISILDNYPKKWRQYYDDANLIKYDP<br>IVDYSNSNHSPINWNIFENNAVNKKSPNVIKEAKTSGLITGFSFPIHMANNGFGMLSFAYSEKDNYIDSLFLHACMNIPL<br>IVPSLVDNYRKINIANNKSNNDLTKREKECLAWACEGKSSWDISKILGCSERTVTFHLTNAQMKLNTTNRCQSISKAILT<br>GAIDCPYFKN	Evolved (This work)
RpaR	MIVGEDQLWGRRALEFVDSVERLEAPALISRFESLIASCGFTAYIMAGLPSRNAGLPELTLANGWPRDWFDLYVSENFSA<br>VDPVPRHGATTVHPFVWSDAPYDRDRDPAAHRVMTRAAEFGLVEGYCIPLHYDDGSAAISMAGKDPDLSPAARGAMQLVS<br>IYAHSRLRALSRPKPIRRNRLTPRECEILQWAAQGKTAWEISVILCITERTVKFHLIEAARKLDAANRTAAVAKALTLGL<br>IRL	(Schaefer *et al*, 2008)
RpaR^{AM2}	MIVGEDQLWGRRTLEFVDSVERLEAPALISRFESLIASCGFTAYIMAGLPSRNAGLPELTLANGWPRDWFDLYVSENFSA<br>VDPVPRYGATTVHPFVWSDAPYDRDRDQAAHRVMTRAAEFGLVEGYCIPLHYDDGSAAISMAGEDPDLSPAARGVMQLVS<br>IYAHSRLRVLSRPKPIRRNRLTPRECEILQWAAQGKTAWEISVILCITERTVKFHLIEAARKLDAANRTAAVAKALTLGL<br>IRL	Evolved (This work)

Appendix Table S3: Sequence of plasmids used in this study

plasmid	DNA sequence [A]
pAJM.431	GACCTCAGCGCTAGCGGAGTGTATACTGGCTTACTATGTTGGCACTGATGAGGGTGTCAGTGAAGTGCTTCATGTGGCAGGAGAAAAAAGGCTGCACCGGTGCG<br>TCAGCAGAATATGTGATACAGGATATATTCCGCTTCCTCGCTCACTGACTCGCTACGCTCGGTCGTTCGACTGCGGCGAGCGGAAATGGCTTACGAACGGGGCG<br>GAGATTTCCTGGAAGATGCCAGGAAGATACTTAACAGGGAAGTGAGAGGGCCGCGGCAAAGCCGTTTTTCCATAGGCTCCGCCCCCCTGACAAGCATCACGAAA<br>TCTGACGCTCAAATCAGTGGTGGCGAAACCCGACAGGACTATAAAGATACCAGGCGTTTCCCCCTGGCGGCTCCCTCGTGCGCTCTCCTGTTCCTGCCTTTCGGT<br>TTACCGGTGTCATTCCGCTGTTATGGCCGCGTTTGTCTCATTCCACGCCTGACACTCAGTTCCGGGTAGGCAGTTCGCTCCAAGCTGGACTGTATGCACGAACC<br>CCCCGTTCAGTCCGACCGCTGCGCCTTATCCGGTAACTATCGTCTTGAGTCCAACCCGGAAAGACATGCAAAAGCACCACTGGCAGCAGCCACTGGTAATTGAT<br>TTAGAGGAGTTAGTCTTGAAGTCATGCGCCGGTTAAGGCTAAACTGAAAGGACAAGTTTTGGTGACTGCGCTCCTCCAAGCCAGTTACCTCGGTTCAAAGAGTT<br>GGTAGCTCAGAGAACCTTCGAAAAACCGCCCTGCAAGGCGGTTTTTTCGTTTTCAGAGCAAGAGATTACGCGCAGACCAAAACGATCTCAAGAAGATCATCTTA<br>TTAAGGGGTCTGACGCTCAGTGGAACGAAAAATCAATCTAAAGTATATATGAGTAAACTTGGTCTGACAGTTACCTTAGAAAAACTCATCGAGCATCAAATGAA<br>ACTGCAATTTATTCATATCAGGATTATCAATACCATATTTTTGAAAAAGCCGTTTCTGTAATGAAGGAGAAAACTCACCGAGGCAGTTCCATAGGATGGCAAGA<br>TCCTGGTATCGGTCTGCGATTCCGACTCGTCCAACATCAATACAACCTATTAATTTCCCCTCGTCAAAAATAAGGTTATCAAGTGAGAAATCACCATGAGTGAC<br>GACTGAATCCGGTGAGAATGGCAAAAGCTTATGCATTTCTTTCCAGACTTGTTCAACAGGCCAGCCATTACGCTCGTCATCAAAATCACTCGCATCAACCAAAC<br>CGTTATTCATTCGTGATTGCGCCTGAGCGAGACGAAATACGCGATCGCTGTTAAAAGGACAATTACAAACAGGAATCGAATGCAACCGGCGCAGGAACACTGCC<br>AGCGCATCAACAATATTTTCACCTGAATCAGGATATTCTTCTAATACCTGGAATGCTGTTTTCCCGGGGATCGCAGTGGTGAGTAACCATGCATCATCAGGAGT<br>ACGGATAAAATGCTTGATGGTCGGAAGAGGCATAAATTCCGTCAGCCAGTTTAGTCTGACCATCTCATCTGTAACATCATTGGCAACGCTACCTTTGCCATGTT<br>TCAGAAACAACTCTGGCGCATCGGGCTTCCCATACAATCGATAGATTGTCGCACCTGATTGCCCGACATTATCGCGAGCCCATTTATACCCATATAAATCAGCA<br>TCCATGTTGGAATTTAATCGCGGCCTCGAGCAAGACGTTTCCCGTTGAATATGGCTCATAACACCCCTTGTATTACTGTTTATGTAAGCAGACAGTTTTATTGT<br>TCATGATGATATATTTTTATCTTGTGCAATGTACATCAGAGATTTTGAGACACAACCAATTATTGAAGGCCTCCCTAACGGGGGGCCTTTTTTTGTTTCTGGTC<br>TCCCGCTTAACGATCGTTGGCTG**TGTTGACAATTAATCATCGGCTCGTATAATGTGTGGAATTGTGAGCGCTCACAATT**AGCTGTCACCGGATGTGCTTTCCGG<br>TCTGATGAGTCCGTGAGGACGAAACAGCCTCTACAAATAATTTTGTTTAAATAAGAGCAAGATAATAAGGAACACTTCTAATGACTATAATGATAAAAAAATCG<br>GATTTTTTGGCAATTCCATCGGAGGAGTATAAAGGTATTCTAAGTCTTCGTTATCAAGTGTTTAAGCAAAGACTTGAGTGGGACTTAGTTGTAGAAAATAACCT<br>TGAATCAGATGAGTATGATAACTCAAATGCAGAATATATTTATGCTTGTGATGATACTGAAAATGTAAGTGGATGCTGGCGTTTATTACCTACAACAGGTGATT<br>ATATGCTGAAAAGTGTTTTTCCTGAATTGCTTGGTCAACAGAGTGCTCCCAAAGATCCTAATATAGTCGAATTAAGTCGTTTTGCTGTAGGTAAAAATAGCTCA<br>AAGATAAATAACTCTGCTAGTGAAATTACAATGAAACTATTTGAAGCTATATATAAACACGCTGTTAGTCAAGGTATTACAGAATATGTAACAGTAACATCAAC<br>AGCAATAGAGCGATTTTTAAAGCGTATTAAAGTTCCTTGTCATCGTATTGGAGACAAAGAAATTCATGTATTAGGTGATACTAAATCGGTTGTATTGTCTATGC<br>CTATTAATGAACAGTTTAAAAAAGCAGTCTTAAATTAACTCGGTACCAAATTCCAGAAAAGAGGCCTCCCGAAAGGGGGGCCTTTTTTCGTTTTGGTCCAATG**G**<br>**CGGCGCGCCATCGAATGGCGCAAAACCTTTCGCGGTATGGCATGATAGCGCCCGGAAGAGAGTCAATTCAGGGTGGTGAAT**ATGAAACCAGTAACGTTATACGA<br>TGTCGCAGAGTATGCCGGTGTCTCTTATATGACCGTTTCCCGCGTGGTGAACCAGGCCAGCCACGTTTCTGCGAAAACGCGGGAAAAAGTGGAAGCGGCGATGG<br>TGGAGCTGAATTACATTCCCAACCGCGTGGCACAACAACTGGCGGGCAAACAGTCGTTGCTGATTGGCGTTGCCACCTCCAGTCTGGCCCTGCACGCGCCGTCG<br>CAAATTGTCGCGGCGATTAAATCTCGCGCCGATCAACTGGGTGCCAGCGTGGTGGTGTCGATGGTAGAACGAAGCGGCGTCGAAGCCTGTAAAGCGGCGGTGCA<br>CAATCTTCTCGCGCAACGCGTCAGTGGGCTGATCATTAACTATCCGCTGGATGACCAGGATGCCATTGCTGTGGAAGCTGCCTGCACTAATGTTCCGGCGTTAT<br>TTCTTGATGTCTCTGACCAGACACCCATCAACAGTATTATTTACTCCCATGAGGACGGTACGCGACTGGGCGTGGAGCATCTGGTCGCATTGGGTCACCAGCAA<br>ATCGCGCTGTTAGCGGGCCCATTAAGTTCTGTCTCGGCGCGTCTGCGTCTGGCTGGCTGGCATAAATATCTCACTCGCAATCAAATTCAGCCGATAGCGGAACG<br>GGAAGGCGACTGGAGTGCCATGTCCGGTTTTCAACAAACCATGCAAATGCTGAATGAGGGCATCGTTCCCACTGCGATGCTGGTTGCCAACGATCAGATGGCGC<br>TGGGCGCAATGCGCGCCATTACCGAGTCCGGGCTGCGCGTTGGTGCGGATATCTCGGTAGTGGGATACGACGATACCGAAGATAGCTCATGTTATATCCCGCCG<br>TTAACCACCATCAAACAGGATTTTCGCCTGCTGGGGCAAACCAGCGTGGACCGCTTGCTGCAACTCTCTCAGGGCCAGGCGGTGAAGGGCAATCAGCTGTTGCC<br>AGTCTCACTGGTGAAAAGAAAAACCACCCTGGCGCCCAATACGCAAACCGCCTCTCCCCGCGCGTTGGCCGATTCATTAATGCAGCTGGCACGACAGGTTTCCC<br>GACTGGAAAGCGGGCAGTGATAAGGATCCTAATTGGTAACGAATCAGACAATTGACGGCTCGAGGGAGTAGCATAGGGTTTGCAGAATCCCTGCTTCGTCCATT<br>TGACAGGCACATTATGCATCGATGATAAGCTGTCAAACATGAGCAGATCCTCTACGCCGGACGCATCGTGGCCGGCATCACCGGCGCCACAGGTGCGGTTGCTG<br>GCGCCTATATCGCCGACATCACCGATGGGGAAGATCGGGCTCGCCACTTCGGGCTCATGAGCAAATATTTTATCTGAGGTGCTTCCTCGCTCACTGACTCGCTG<br>CACGAGGCA
pAJM.715 [B]	GACCTCAGCGCTAGCGGAGTGTATACTGGCTTACTATGTTGGCACTGATGAGGGTGTCAGTGAAGTGCTTCATGTGGCAGGAGAAAAAAGGCTGCACCGGTGCG<br>TCAGCAGAATATGTGATACAGGATATATTCCGCTTCCTCGCTCACTGACTCGCTACGCTCGGTCGTTCGACTGCGGCGAGCGGAAATGGCTTACGAACGGGGCG<br>GAGATTTCCTGGAAGATGCCAGGAAGATACTTAACAGGGAAGTGAGAGGGCCGCGGCAAAGCCGTTTTTCCATAGGCTCCGCCCCCCTGACAAGCATCACGAAA<br>TCTGACGCTCAAATCAGTGGTGGCGAAACCCGACAGGACTATAAAGATACCAGGCGTTTCCCCCTGGCGGCTCCCTCGTGCGCTCTCCTGTTCCTGCCTTTCGG<br>TTTACCGGTGTCATTCCGCTGTTATGGCCGCGTTTGTCTCATTCCACGCCTGACACTCAGTTCCGGGTAGGCAGTTCGCTCCAAGCTGGACTGTATGCACGAAC<br>CCCCCGTTCAGTCCGACCGCTGCGCCTTATCCGGTAACTATCGTCTTGAGTCCAACCCGGAAAGACATGCAAAAGCACCACTGGCAGCAGCCACTGGTAATTGA<br>TTTAGAGGAGTTAGTCTTGAAGTCATGCGCCGGTTAAGGCTAAACTGAAAGGACAAGTTTTGGTGACTGCGCTCCTCCAAGCCAGTTACCTCGGTTCAAAGAGT<br>TGGTAGCTCAGAGAACCTTCGAAAAACCGCCCTGCAAGGCGGTTTTTTCGTTTTCAGAGCAAGAGATTACGCGCAGACCAAAACGATCTCAAGAAGATCATCTT<br>ATTAAGGGGTCTGACGCTCAGTGGAACGAAAAATCAATCTAAAGTATATATGAGTAAACTTGGTCTGACAGTTACCTTAGAAAAACTCATCGAGCATCAAATGA<br>AACTGCAATTTATTCATATCAGGATTATCAATACCATATTTTTGAAAAAGCCGTTTCTGTAATGAAGGAGAAAACTCACCGAGGCAGTTCCATAGGATGGCAAG<br>ATCCTGGTATCGGTCTGCGATTCCGACTCGTCCAACATCAATACAACCTATTAATTTCCCCTCGTCAAAAATAAGGTTATCAAGTGAGAAATCACCATGAGTGA<br>CGACTGAATCCGGTGAGAATGGCAAAAGCTTATGCATTTCTTTCCAGACTTGTTCAACAGGCCAGCCATTACGCTCGTCATCAAAATCACTCGCATCAACCAAA<br>CCGTTATTCATTCGTGATTGCGCCTGAGCGAGACGAAATACGCGATCGCTGTTAAAAGGACAATTACAAACAGGAATCGAATGCAACCGGCGCAGGAACACTGC<br>CAGCGCATCAACAATATTTTCACCTGAATCAGGATATTCTTCTAATACCTGGAATGCTGTTTTCCCGGGGATCGCAGTGGTGAGTAACCATGCATCATCAGGAG<br>TACGGATAAAATGCTTGATGGTCGGAAGAGGCATAAATTCCGTCAGCCAGTTTAGTCTGACCATCTCATCTGTAACATCATTGGCAACGCTACCTTTGCCATGT<br>TTCAGAAACAACTCTGGCGCATCGGGCTTCCCATACAATCGATAGATTGTCGCACCTGATTGCCCGACATTATCGCGAGCCCATTTATACCCATATAAATCAGC<br>ATCCATGTTGGAATTTAATCGCGGCCTCGAGCAAGACGTTTCCCGTTGAATATGGCTCATAACACCCCTTGTATTACTGTTTATGTAAGCAGACAGTTTTATTG<br>TTCATGATGATATATTTTTATCTTGTGCAATGTACATCAGAGATTTTGAGACACAACCAATTATTGAAGGCCTCCCTAACGGGGGGCCTTTTTTTGTTTCTGGT<br>CTCCCGCTTAACGATCGTTGGCTG**TGTTGACAATTAATCATCGGCTCGTATAATGTGTGGAATTGTGAGCGCTCACAATT**AGCTGTCACCGGATGTGCTTTCCG<br>GTCTGATGAGTCCGTGAGGACGAAACAGCCTCTACAAATAATTTTGTTTAATACTAGAGAAAGAGGGGAAATACTAGATGGTGAGCAAGGGCGAGGAGCTGTTC<br>ACCGGGGTGGTGCCCATCCTGGTCGAGCTGGACGGCGACGTAAACGGCCACAAGTTCAGCGTGTCCGGCGAGGGCGAGGGCGATGCCACCTACGGCAAGCTGAC<br>CCTGAAGTTCATCTGCACCACAGGCAAGCTGCCCGTGCCCTGGCCCACCCTCGTGACCACCTTCGGCTACGGCCTGCAATGCTTCGCCCGCTACCCCGACCACA<br>TGAAGCTGCACGACTTCTTCAAGTCCGCCATGCCCGAAGGCTACGTCCAGGAGCGCACCATCTTCTTCAAGGACGACGGCAACTACAAGACCCGCGCCGAGGTG<br>AAGTTCGAGGGCGACACCCTGGTGAACCGCATCGAGCTGAAGGGCATCGACTTCAAGGAGGACGGCAACATCCTGGGGCACAAGCTGGAGTACAACTACAACAG<br>CCACAACGTCTATATCATGGCCGACAAGCAGAAGAACGGCATCAAGGTGAACTTCAAGATCCGCCACAACATCGAGGACGGCAGCGTGCAGCTCGCCGACCACT<br>ACCAGCAGAACACCCCAATCGGCGACGGCCCCGTGCTGCTGCCCGACAACCACTACCTTAGCTACCAGTCCGCCCTGAGCAAAGACCCCAACGAGAAGCGCGAT<br>CACATGGTCCTGCTGGAGTTCGTGACCGCCGCCGGGATCACTCTCGGCATGGACGAGCTGTACAAGTAACTCGGTACCAAATTCCAGAAAAGAGGCCTCCCGAA<br>AGGGGGGCCTTTTTTCGTTTTGGTCCAATGGCGGCGCGCCATCGAATGAGGTGCTTCCTCGCTCACTGACTCGCTGCACGAGGCA
pAJM.716 [B]	GACCTCAGCGCTAGCGGAGTGTATACTGGCTTACTATGTTGGCACTGATGAGGGTGTCAGTGAAGTGCTTCATGTGGCAGGAGAAAAAAGGCTGCACCGGTGCG<br>TCAGCAGAATATGTGATACAGGATATATTCCGCTTCCTCGCTCACTGACTCGCTACGCTCGGTCGTTCGACTGCGGCGAGCGGAAATGGCTTACGAACGGGGCG<br>GAGATTTCCTGGAAGATGCCAGGAAGATACTTAACAGGGAAGTGAGAGGGCCGCGGCAAAGCCGTTTTTCCATAGGCTCCGCCCCCCTGACAAGCATCACGAAA<br>TCTGACGCTCAAATCAGTGGTGGCGAAACCCGACAGGACTATAAAGATACCAGGCGTTTCCCCCTGGCGGCTCCCTCGTGCGCTCTCCTGTTCCTGCCTTTCGG<br>TTTACCGGTGTCATTCCGCTGTTATGGCCGCGTTTGTCTCATTCCACGCCTGACACTCAGTTCCGGGTAGGCAGTTCGCTCCAAGCTGGACTGTATGCACGAAC<br>CCCCCGTTCAGTCCGACCGCTGCGCCTTATCCGGTAACTATCGTCTTGAGTCCAACCCGGAAAGACATGCAAAAGCACCACTGGCAGCAGCCACTGGTAATTGA<br>TTTAGAGGAGTTAGTCTTGAAGTCATGCGCCGGTTAAGGCTAAACTGAAAGGACAAGTTTTGGTGACTGCGCTCCTCCAAGCCAGTTACCTCGGTTCAAAGAGT<br>TGGTAGCTCAGAGAACCTTCGAAAAACCGCCCTGCAAGGCGGTTTTTTCGTTTTCAGAGCAAGAGATTACGCGCAGACCAAAACGATCTCAAGAAGATCATCTT<br>ATTAAGGGGTCTGACGCTCAGTGGAACGAAAAATCAATCTAAAGTATATATGAGTAAACTTGGTCTGACAGTTACCTTAGAAAAACTCATCGAGCATCAAATGA<br>AACTGCAATTTATTCATATCAGGATTATCAATACCATATTTTTGAAAAAGCCGTTTCTGTAATGAAGGAGAAAACTCACCGAGGCAGTTCCATAGGATGGCAAG<br>ATCCTGGTATCGGTCTGCGATTCCGACTCGTCCAACATCAATACAACCTATTAATTTCCCCTCGTCAAAAATAAGGTTATCAAGTGAGAAATCACCATGAGTGA<br>CGACTGAATCCGGTGAGAATGGCAAAAGCTTATGCATTTCTTTCCAGACTTGTTCAACAGGCCAGCCATTACGCTCGTCATCAAAATCACTCGCATCAACCAAA<br>CCGTTATTCATTCGTGATTGCGCCTGAGCGAGACGAAATACGCGATCGCTGTTAAAAGGACAATTACAAACAGGAATCGAATGCAACCGGCGCAGGAACACTGC<br>CAGCGCATCAACAATATTTTCACCTGAATCAGGATATTCTTCTAATACCTGGAATGCTGTTTTCCCGGGGATCGCAGTGGTGAGTAACCATGCATCATCAGGAG

	TACGGATAAAATGCTTGATGGTCGGAAGAGGCATAAATTCCGTCAGCCAGTTTAGTCTGACCATCTCATCTGTAACATCATTGGCAACGCTACCTTTGCCATGT<br>TTCAGAAACAACTCTGGCGCATCGGGCTTCCCATACAATCGATAGATTGTCGCACCTGATTGCCCGACATTATCGCGAGCCCATTTATACCCATATAAATCAGC<br>ATCCATGTTGGAATTTAATCGCGGCCTCGAGCAAGACGTTTCCCGTTGAATATGGCTCATAACACCCCTTGTATTACTGTTTATGTAAGCAGACAGTTTTATTG<br>TTCATGATGATATATTTTTATCTTGTGCAATGTACATCAGAGATTTTGAGACACAACCAATTATTGAAGGCCTCCCTAACGGGGGGCCTTTTTTTGTTTCTGGT<br>CTCCCGCTTAACGATCGTTGGCTG**AGAAACCAATTGTCCATATTGCATCAGACATTGCCGTCACTGCGTCTTTTACTGGCTCTTCTCGCTAACCAAACCGGTAA**<br>**CCCCGCTTATTAAAAGCATTCTGTAACAAAGCGGGACCAAAGCCATGACAAAAACGCGTAACAAAAGTGTCTATAATCACGGCAGAAAAGTCCACATTGATTAT**<br>**TTGCACGGCGTCACACTTTGCTATGCCATAGCATTTTTATCCATAAGATTAGCGGATCCTACCTGACGCTTTTTATCGCAACTCTCTACTGTTTCTCCATACCC**<br>**G**AGCTGTCACCGGATGTGCTTTCCGGTCTGATGAGTCCGTGAGGACGAAACAGCCTCTACAAATAATTTTGTTTAATACTAGAGAAAGAGGGGAAATACTAGAT<br>GGTGAGCAAGGGCGAGGAGCTGTTCACCGGGGTGGTGCCCATCCTGGTCGAGCTGGACGGCGACGTAAACGGCCACAAGTTCAGCGTGTCCGGCGAGGGCGAGG<br>GCGATGCCACCTACGGCAAGCTGACCCTGAAGTTCATCTGCACCACAGGCAAGCTGCCCGTGCCCTGGCCCACCCTCGTGACCACCTTCGGCTACGGCCTGCAA<br>TGCTTCGCCCGCTACCCCGACCACATGAAGCTGCACGACTTCTTCAAGTCCGCCATGCCCGAAGGCTACGTCCAGGAGCGCACCATCTTCTTCAAGGACGACGG<br>CAACTACAAGACCCGCGCCGAGGTGAAGTTCGAGGGCGACACCCTGGTGAACCGCATCGAGCTGAAGGGCATCGACTTCAAGGAGGACGGCAACATCCTGGGGC<br>ACAAGCTGGAGTACAACTACAACAGCCACAACGTCTATATCATGGCCGACAAGCAGAAGAACGGCATCAAGGTGAACTTCAAGATCCGCCACAACATCGAGGAC<br>GGCAGCGTGCAGCTCGCCGACCACTACCAGCAGAACACCCCAATCGGCGACGGCCCCGTGCTGCTGCCCGACAACCACTACCTTAGCTACCAGTCCGCCCTGAG<br>CAAAGACCCCAACGAGAAGCGCGATCACATGGTCCTGCTGGAGTTCGTGACCGCCGCCGGGATCACTCTCGGCATGGACGAGCTGTACAAGTAACTCGGTACCA<br>AATTCCAGAAAAGAGGCCTCCCGAAAGGGGGGCCTTTTTTCGTTTTGGTCCAATGGCGGCGCGCCATCGAATGAGGTGCTTCCTCGCTCACTGACTCGCTGCAC<br>GAGGCA
pAJM.717 [B]	GACCTCAGCGCTAGCGGAGTGTATACTGGCTTACTATGTTGGCACTGATGAGGGTGTCAGTGAAGTGCTTCATGTGGCAGGAGAAAAAAGGCTGCACCGGTGCG<br>TCAGCAGAATATGTGATACAGGATATATTCCGCTTCCTCGCTCACTGACTCGCTACGCTCGGTCGTTCGACTGCGGCGAGCGGAAATGGCTTACGAACGGGGCG<br>GAGATTTCCTGGAAGATGCCAGGAAGATACTTAACAGGGAAGTGAGAGGGCCGCGGCAAAGCCGTTTTTCCATAGGCTCCGCCCCCCTGACAAGCATCACGAAA<br>TCTGACGCTCAAATCAGTGGTGGCGAAACCCGACAGGACTATAAAGATACCAGGCGTTTCCCCCTGGCGGCTCCCTCGTGCGCTCTCCTGTTCCTGCCTTTCGG<br>TTTACCGGTGTCATTCCGCTGTTATGGCCGCGTTTGTCTCATTCCACGCCTGACACTCAGTTCCGGGTAGGCAGTTCGCTCCAAGCTGGACTGTATGCACGAAC<br>CCCCCGTTCAGTCCGACCGCTGCGCCTTATCCGGTAACTATCGTCTTGAGTCCAACCCGGAAAGACATGCAAAAGCACCACTGGCAGCAGCCACTGGTAATTGA<br>TTTAGAGGAGTTAGTCTTGAAGTCATGCGCCGGTTAAGGCTAAACTGAAAGGACAAGTTTTGGTGACTGCGCTCCTCCAAGCCAGTTACCTCGGTTCAAAGAGT<br>TGGTAGCTCAGAGAACCTTCGAAAAACCGCCCTGCAAGGCGGTTTTTTCGTTTTCAGAGCAAGAGATTACGCGCAGACCAAAACGATCTCAAGAAGATCATCTT<br>ATTAAGGGGTCTGACGCTCAGTGGAACGAAAAATCAATCTAAAGTATATATGAGTAAACTTGGTCTGACAGTTACCTTAGAAAAACTCATCGAGCATCAAATGA<br>AACTGCAATTTATTCATATCAGGATTATCAATACCATATTTTTGAAAAAGCCGTTTCTGTAATGAAGGAGAAAACTCACCGAGGCAGTTCCATAGGATGGCAAG<br>ATCCTGGTATCGGTCTGCGATTCCGACTCGTCCAACATCAATACAACCTATTAATTTCCCCTCGTCAAAAATAAGGTTATCAAGTGAGAAATCACCATGAGTGA<br>CGACTGAATCCGGTGAGAATGGCAAAAGCTTATGCATTTCTTTCCAGACTTGTTCAACAGGCCAGCCATTACGCTCGTCATCAAAATCACTCGCATCAACCAAA<br>CCGTTATTCATTCGTGATTGCGCCTGAGCGAGACGAAATACGCGATCGCTGTTAAAAGGACAATTACAAACAGGAATCGAATGCAACCGGCGCAGGAACACTGC<br>CAGCGCATCAACAATATTTTCACCTGAATCAGGATATTCTTCTAATACCTGGAATGCTGTTTTCCCGGGGATCGCAGTGGTGAGTAACCATGCATCATCAGGAG<br>TACGGATAAAATGCTTGATGGTCGGAAGAGGCATAAATTCCGTCAGCCAGTTTAGTCTGACCATCTCATCTGTAACATCATTGGCAACGCTACCTTTGCCATGT<br>TTCAGAAACAACTCTGGCGCATCGGGCTTCCCATACAATCGATAGATTGTCGCACCTGATTGCCCGACATTATCGCGAGCCCATTTATACCCATATAAATCAGC<br>ATCCATGTTGGAATTTAATCGCGGCCTCGAGCAAGACGTTTCCCGTTGAATATGGCTCATAACACCCCTTGTATTACTGTTTATGTAAGCAGACAGTTTTATTG<br>TTCATGATGATATATTTTTATCTTGTGCAATGTACATCAGAGATTTTGAGACACAACCAATTATTGAAGGCCTCCCTAACGGGGGGCCTTTTTTTGTTTCTGGT<br>CTCCCGCTTAACGATCGTTGGCTG**TTTTCAGCAGGACGCACTGACCTCCCTATCAGTGATAGAGATTGACATCCCTATCAGTGATAGAGATACTGAGCAC**AGCT<br>GTCACCGGATGTGCTTTCCGGTCTGATGAGTCCGTGAGGACGAAACAGCCTCTACAAATAATTTTGTTTAATACTAGAGAAAGAGGGGAAATACTAGATGGTGA<br>GCAAGGGCGAGGAGCTGTTCACCGGGGTGGTGCCCATCCTGGTCGAGCTGGACGGCGACGTAAACGGCCACAAGTTCAGCGTGTCCGGCGAGGGCGAGGGCGAT<br>GCCACCTACGGCAAGCTGACCCTGAAGTTCATCTGCACCACAGGCAAGCTGCCCGTGCCCTGGCCCACCCTCGTGACCACCTTCGGCTACGGCCTGCAATGCTT<br>CGCCCGCTACCCCGACCACATGAAGCTGCACGACTTCTTCAAGTCCGCCATGCCCGAAGGCTACGTCCAGGAGCGCACCATCTTCTTCAAGGACGACGGCAACT<br>ACAAGACCCGCGCCGAGGTGAAGTTCGAGGGCGACACCCTGGTGAACCGCATCGAGCTGAAGGGCATCGACTTCAAGGAGGACGGCAACATCCTGGGGCACAAG<br>CTGGAGTACAACTACAACAGCCACAACGTCTATATCATGGCCGACAAGCAGAAGAACGGCATCAAGGTGAACTTCAAGATCCGCCACAACATCGAGGACGGCAG<br>CGTGCAGCTCGCCGACCACTACCAGCAGAACACCCCAATCGGCGACGGCCCCGTGCTGCTGCCCGACAACCACTACCTTAGCTACCAGTCCGCCCTGAGCAAAG<br>ACCCCAACGAGAAGCGCGATCACATGGTCCTGCTGGAGTTCGTGACCGCCGCCGGGATCACTCTCGGCATGGACGAGCTGTACAAGTAACTCGGTACCAAATTC<br>CAGAAAAGAGGCCTCCCGAAAGGGGGGCCTTTTTTCGTTTTGGTCCAATGGCGGCGCGCCATCGAATGAGGTGCTTCCTCGCTCACTGACTCGCTGCACGAGGC<br>A
pAJM.944	GACCTCAGCGCTAGCGGAGTGTATACTGGCTTACTATGTTGGCACTGATGAGGGTGTCAGTGAAGTGCTTCATGTGGCAGGAGAAAAAAGGCTGCACCGGTGCG<br>TCAGCAGAATATGTGATACAGGATATATTCCGCTTCCTCGCTCACTGACTCGCTACGCTCGGTCGTTCGACTGCGGCGAGCGGAAATGGCTTACGAACGGGGCG<br>GAGATTTCCTGGAAGATGCCAGGAAGATACTTAACAGGGAAGTGAGAGGGCCGCGGCAAAGCCGTTTTTCCATAGGCTCCGCCCCCCTGACAAGCATCACGAAA<br>TCTGACGCTCAAATCAGTGGTGGCGAAACCCGACAGGACTATAAAGATACCAGGCGTTTCCCCCTGGCGGCTCCCTCGTGCGCTCTCCTGTTCCTGCCTTTCGGT<br>TTACCGGTGTCATTCCGCTGTTATGGCCGCGTTTGTCTCATTCCACGCCTGACACTCAGTTCCGGGTAGGCAGTTCGCTCCAAGCTGGACTGTATGCACGAACC<br>CCCCGTTCAGTCCGACCGCTGCGCCTTATCCGGTAACTATCGTCTTGAGTCCAACCCGGAAAGACATGCAAAAGCACCACTGGCAGCAGCCACTGGTAATTGAT<br>TTAGAGGAGTTAGTCTTGAAGTCATGCGCCGGTTAAGGCTAAACTGAAAGGACAAGTTTTGGTGACTGCGCTCCTCCAAGCCAGTTACCTCGGTTCAAAGAGTT<br>GGTAGCTCAGAGAACCTTCGAAAAACCGCCCTGCAAGGCGGTTTTTTCGTTTTCAGAGCAAGAGATTACGCGCAGACCAAAACGATCTCAAGAAGATCATCTTA<br>TTAAGGGGTCTGACGCTCAGTGGAACGAAAAATCAATCTAAAGTATATATGAGTAAACTTGGTCTGACAGTTACCTTAGAAAAACTCATCGAGCATCAAATGAA<br>ACTGCAATTTATTCATATCAGGATTATCAATACCATATTTTTGAAAAAGCCGTTTCTGTAATGAAGGAGAAAACTCACCGAGGCAGTTCCATAGGATGGCAAGA<br>TCCTGGTATCGGTCTGCGATTCCGACTCGTCCAACATCAATACAACCTATTAATTTCCCCTCGTCAAAAATAAGGTTATCAAGTGAGAAATCACCATGAGTGAC<br>GACTGAATCCGGTGAGAATGGCAAAAGCTTATGCATTTCTTTCCAGACTTGTTCAACAGGCCAGCCATTACGCTCGTCATCAAAATCACTCGCATCAACCAAAC<br>CGTTATTCATTCGTGATTGCGCCTGAGCGAGACGAAATACGCGATCGCTGTTAAAAGGACAATTACAAACAGGAATCGAATGCAACCGGCGCAGGAACACTGCC<br>AGCGCATCAACAATATTTTCACCTGAATCAGGATATTCTTCTAATACCTGGAATGCTGTTTTCCCGGGGATCGCAGTGGTGAGTAACCATGCATCATCAGGAGT<br>ACGGATAAAATGCTTGATGGTCGGAAGAGGCATAAATTCCGTCAGCCAGTTTAGTCTGACCATCTCATCTGTAACATCATTGGCAACGCTACCTTTGCCATGTT<br>TCAGAAACAACTCTGGCGCATCGGGCTTCCCATACAATCGATAGATTGTCGCACCTGATTGCCCGACATTATCGCGAGCCCATTTATACCCATATAAATCAGCA<br>TCCATGTTGGAATTTAATCGCGGCCTCGAGCAAGACGTTTCCCGTTGAATATGGCTCATAACACCCCTTGTATTACTGTTTATGTAAGCAGACAGTTTTATTGT<br>TCATGATGATATATTTTTATCTTGTGCAATGTACATCAGAGATTTTGAGACACAACCAATTATTGAAGGCCTCCCTAACGGGGGGCCTTTTTTTGTTTCTGGTC<br>TCCCGCTTAACGATCGTTGGCTG**CCCTTTGTGCGTCCAAACGGACGCACGGCGCTCTAAAGCGGGTCGCGATCTTTCAGATTCGCTCCTCGCGCTTTCAGTCTT**<br>**TGTTTTGGCGCATGTCGTTATCGCAAAACCGCTGCACACTTTTGCGCGACATGCTCTGATCCCCCTCATCTGGGGGGGCCTATCTGAGGGAATTTCCGATCCGG**<br>**CTCGCCTGAACCATTCTGCTTTCCACGAACTTGAAAACGCT**AGCTGTCACCGGATGTGCTTTCCGGTCTGATGAGTCCGTGAGGACGAAACAGCCTCTACAAAT<br>AATTTTGTTTAATACTAGAGAAAGAGGGGAAATACTAGATGGTGAGCAAGGGCGAGGAGCTGTTCACCGGGGTGGTGCCCATCCTGGTCGAGCTGGACGGCGAC<br>GTAAACGGCCACAAGTTCAGCGTGTCCGGCGAGGGCGAGGGCGATGCCACCTACGGCAAGCTGACCCTGAAGTTCATCTGCACCACAGGCAAGCTGCCCGTGCC<br>CTGGCCCACCCTCGTGACCACCTTCGGCTACGGCCTGCAATGCTTCGCCCGCTACCCCGACCACATGAAGCTGCACGACTTCTTCAAGTCCGCCATGCCCGAAG<br>GCTACGTCCAGGAGCGCACCATCTTCTTCAAGGACGACGGCAACTACAAGACCCGCGCCGAGGTGAAGTTCGAGGGCGACACCCTGGTGAACCGCATCGAGCTG<br>AAGGGCATCGACTTCAAGGAGGACGGCAACATCCTGGGGCACAAGCTGGAGTACAACTACAACAGCCACAACGTCTATATCATGGCCGACAAGCAGAAGAACGG<br>CATCAAGGTGAACTTCAAGATCCGCCACAACATCGAGGACGGCAGCGTGCAGCTCGCCGACCACTACCAGCAGAACACCCCAATCGGCGACGGCCCCGTGCTGC<br>TGCCCGACAACCACTACCTTAGCTACCAGTCCGCCCTGAGCAAAGACCCCAACGAGAAGCGCGATCACATGGTCCTGCTGGAGTTCGTGACCGCCGCCGGGATC<br>ACTCTCGGCATGGACGAGCTGTACAAGTAACTCGGTACCAAATTCCAGAAAAGAGGCCTCCCGAAAGGGGGGCCTTTTTTCGTTTTGGTCCAATGGCGGCGCGC<br>CATCGAATGAGGTGCTTCCTCGCTCACTGACTCGCTGCACGAGGCA
pAJM.947	GACCTCAGCGCTAGCGGAGTGTATACTGGCTTACTATGTTGGCACTGATGAGGGTGTCAGTGAAGTGCTTCATGTGGCAGGAGAAAAAAGGCTGCACCGGTGCG<br>TCAGCAGAATATGTGATACAGGATATATTCCGCTTCCTCGCTCACTGACTCGCTACGCTCGGTCGTTCGACTGCGGCGAGCGGAAATGGCTTACGAACGGGGCG<br>GAGATTTCCTGGAAGATGCCAGGAAGATACTTAACAGGGAAGTGAGAGGGCCGCGGCAAAGCCGTTTTTCCATAGGCTCCGCCCCCCTGACAAGCATCACGAAA<br>TCTGACGCTCAAATCAGTGGTGGCGAAACCCGACAGGACTATAAAGATACCAGGCGTTTCCCCCTGGCGGCTCCCTCGTGCGCTCTCCTGTTCCTGCCTTTCGGT<br>TTACCGGTGTCATTCCGCTGTTATGGCCGCGTTTGTCTCATTCCACGCCTGACACTCAGTTCCGGGTAGGCAGTTCGCTCCAAGCTGGACTGTATGCACGAACC<br>CCCCGTTCAGTCCGACCGCTGCGCCTTATCCGGTAACTATCGTCTTGAGTCCAACCCGGAAAGACATGCAAAAGCACCACTGGCAGCAGCCACTGGTAATTGAT<br>TTAGAGGAGTTAGTCTTGAAGTCATGCGCCGGTTAAGGCTAAACTGAAAGGACAAGTTTTGGTGACTGCGCTCCTCCAAGCCAGTTACCTCGGTTCAAAGAGTT<br>GGTAGCTCAGAGAACCTTCGAAAAACCGCCCTGCAAGGCGGTTTTTTCGTTTTCAGAGCAAGAGATTACGCGCAGACCAAAACGATCTCAAGAAGATCATCTTA<br>TTAAGGGGTCTGACGCTCAGTGGAACGAAAAATCAATCTAAAGTATATATGAGTAAACTTGGTCTGACAGTTACCTTAGAAAAACTCATCGAGCATCAAATGAA<br>ACTGCAATTTATTCATATCAGGATTATCAATACCATATTTTTGAAAAAGCCGTTTCTGTAATGAAGGAGAAAACTCACCGAGGCAGTTCCATAGGATGGCAAGA<br>TCCTGGTATCGGTCTGCGATTCCGACTCGTCCAACATCAATACAACCTATTAATTTCCCCTCGTCAAAAATAAGGTTATCAAGTGAGAAATCACCATGAGTGAC<br>GACTGAATCCGGTGAGAATGGCAAAAGCTTATGCATTTCTTTCCAGACTTGTTCAACAGGCCAGCCATTACGCTCGTCATCAAAATCACTCGCATCAACCAAAC<br>CGTTATTCATTCGTGATTGCGCCTGAGCGAGACGAAATACGCGATCGCTGTTAAAAGGACAATTACAAACAGGAATCGAATGCAACCGGCGCAGGAACACTGCC<br>AGCGCATCAACAATATTTTCACCTGAATCAGGATATTCTTCTAATACCTGGAATGCTGTTTTCCCGGGGATCGCAGTGGTGAGTAACCATGCATCATCAGGAGT<br>ACGGATAAAATGCTTGATGGTCGGAAGAGGCATAAATTCCGTCAGCCAGTTTAGTCTGACCATCTCATCTGTAACATCATTGGCAACGCTACCTTTGCCATGTT<br>TCAGAAACAACTCTGGCGCATCGGGCTTCCCATACAATCGATAGATTGTCGCACCTGATTGCCCGACATTATCGCGAGCCCATTTATACCCATATAAATCAGCA<br>TCCATGTTGGAATTTAATCGCGGCCTCGAGCAAGACGTTTCCCGTTGAATATGGCTCATAACACCCCTTGTATTACTGTTTATGTAAGCAGACAGTTTTATTGT<br>TCATGATGATATATTTTTATCTTGTGCAATGTACATCAGAGATTTTGAGACACAACCAATTATTGAAGGCCTCCCTAACGGGGGGCCTTTTTTTGTTTCTGGTC

	TCCCGCTTAACGATCGTTGGCTG**ACCTGTCCGATCGGACAATATTACGCAAGAAAATGGTTTGTTACTTTCGAATAAA**AGCTGTCACCGGATGTGCTTTCCGGT<br>CTGATGAGTCCGTGAGGACGAAACAGCCTCTACAAATAATTTTGTTTAATACTAGAGAAAGAGGGGAAATACTAGATGGTGAGCAAGGGCGAGGAGCTGTTCAC<br>CGGGGTGGTGCCCATCCTGGTCGAGCTGGACGGCGACGTAAACGGCCACAAGTTCAGCGTGTCCGGCGAGGGCGAGGGCGATGCCACCTACGGCAAGCTGACCC<br>TGAAGTTCATCTGCACCACAGGCAAGCTGCCCGTGCCCTGGCCCACCCTCGTGACCACCTTCGGCTACGGCCTGCAATGCTTCGCCCGCTACCCCGACCACATG<br>AAGCTGCACGACTTCTTCAAGTCCGCCATGCCCGAAGGCTACGTCCAGGAGCGCACCATCTTCTTCAAGGACGACGGCAACTACAAGACCCGCGCCGAGGTGAA<br>GTTCGAGGGCGACACCCTGGTGAACCGCATCGAGCTGAAGGGCATCGACTTCAAGGAGGACGGCAACATCCTGGGGCACAAGCTGGAGTACAACTACAACAGCC<br>ACAACGTCTATATCATGGCCGACAAGCAGAAGAACGGCATCAAGGTGAACTTCAAGATCCGCCACAACATCGAGGACGGCAGCGTGCAGCTCGCCGACCACTAC<br>CAGCAGAACACCCCCAATCGGCGACGGCCCCGTGCTGCTGCCCGACAACCACTACCTTAGCTACCAGTCCGCCCTGAGCAAAGACCCCAACGAGAAGCGCGATCA<br>CATGGTCCTGCTGGAGTTCGTGACCGCCGCCGGGATCACTCTCGGCATGGACGAGCTGTACAAGTAACTCGGTACCAAATTCCAGAAAAGAGGCCTCCCGAAAG<br>GGGGGCCTTTTTTCGTTTTGGTCCAATGGCGGCGCGCCATCGAATGAGGTGCTTCCTCGCTCACTGACTCGCTGCACGAGGCA
pAJM.1246	GACCTCAGCGCTAGCGGAGTGTATACTGGCTTACTATGTTGGCACTGATGAGGGTGTCAGTGAAGTGCTTCATGTGGCAGGAGAAAAAAGGCTGCACCGGTGCG<br>TCAGCAGAATATGTGATACAGGATATATTCCGCTTCCTCGCTCACTGACTCGCTACGCTCGGTCGTTCGACTGCGGCGAGCGGAAATGGCTTACGAACGGGGCG<br>GAGATTTCCTGGAAGATGCCAGGAAGATACTTAACAGGGAAGTGAGAGGGCCGCGGCAAAGCCGTTTTTCCATAGGCTCCGCCCCCCTGACAAGCATCACGAAA<br>TCTGACGCTCAAATCAGTGGTGGCGAAACCCGACAGGACTATAAAGATACCAGGCGTTTCCCCTGGCGGCTCCCTCGTGCGCTCTCCTGTTCCTGCCTTTCGGT<br>TTACCGGTGTCATTCCGCTGTTATGGCCGCGTTTGTCTCATTCCACGCCTGACACTCAGTTCCGGGTAGGCAGTTCGCTCCAAGCTGGACTGTATGCACGAACC<br>CCCCGTTCAGTCCGACCGCTGCGCCTTATCCGGTAACTATCGTCTTGAGTCCAACCCGGAAAGACATGCAAAAGCACCACTGGCAGCAGCCACTGGTAATTGAT<br>TTAGAGGAGTTAGTCTTGAAGTCATGCGCCGGTTAAGGCTAAACTGAAAGGACAAGTTTTGGTGACTGCGCTCCTCCAAGCCAGTTACCTCGGTTCAAAGAGTT<br>GGTAGCTCAGAGAACCTTCGAAAAACCGCCCTGCAAGGCGGTTTTTTCGTTTTCAGAGCAAGAGATTACGCGCAGACCAAAACGATCTCAAGAAGATCATCTTA<br>TTAAGGGGTCTGACGCTCAGTGGAACGAAAAATCAATCTAAAGTATATATGAGTAAACTTGGTCTGACAGTTACCTTAGAAAAACTCATCGAGCATCAAATGAA<br>ACTGCAATTTATTCATATCAGGATTATCAATACCATATTTTTGAAAAAGCCGTTTCTGTAATGAAGGAGAAAACTCACCGAGGCAGTTCCATAGGATGGCAAGA<br>TCCTGGTATCGGTCTGCGATTCCGACTCGTCCAACATCAATACAACCTATTAATTTCCCCTCGTCAAAAATAAGGTTATCAAGTGAGAAATCACCATGAGTGAC<br>GACTGAATCCGGTGAGAATGGCAAAAGCTTATGCATTTCTTTCCAGACTTGTTCAACAGGCCAGCCATTACGCTCGTCATCAAAATCACTCGCATCAACCAAAC<br>CGTTATTCATTCGTGATTGCGCCTGAGCGAGACGAAATACGCGATCGCTGTTAAAAGGACAATTACAAACAGGAATCGAATGCAACCGGCGCAGGAACACTGCC<br>AGCGCATCAACAATATTTTCACCTGAATCAGGATATTCTTCTAATACCTGGAATGCTGTTTTCCCGGGGATCGCAGTGGTGAGTAACCATGCATCATCAGGAGT<br>ACGGATAAAATGCTTGATGGTCGGAAGAGGCATAAATTCCGTCAGCCAGTTTAGTCTGACCATCTCATCTGTAACATCATTGGCAACGCTACCTTTGCCATGTT<br>TCAGAAACAACTCTGGCGCATCGGGCTTCCCATACAATCGATAGATTGTCGCACCTGATTGCCCGACATTATCGCGAGCCCATTTATACCCATATAAATCAGCA<br>TCCATGTTGGAATTTAATCGCGGCCTCGAGCAAGACGTTTCCCGTTGAATATGGCTCATAACACCCCTTGTATTACTGTTTATGTAAGCAGACAGTTTTATTGT<br>TCATGATGATATATTTTTATCTTGTGCAATGTACATCAGAGATTTTGAGACACAACCAATTATTGAAGGCCTCCCTAACGGGGGGCCTTTTTTTGTTTCTGGTC<br>TCCCGCTTAACGATCGTTGGCTG**ACCTGTAGGATCTTACAAGTTTACGCAAGAAAATGGTTTGTTACTTTCGAATAAA**AGCTGTCACCGGATGTGCTTTCCGGT<br>CTGATGAGTCCGTGAGGACGAAACAGCCTCTACAAATAATTTTGTTTAATACTAGAGAAAGAGGGGAAATACTAGATGGTGAGCAAGGGCGAGGAGCTGTTCAC<br>CGGGGTGGTGCCCATCCTGGTCGAGCTGGACGGCGACGTAAACGGCCACAAGTTCAGCGTGTCCGGCGAGGGCGAGGGCGATGCCACCTACGGCAAGCTGACCC<br>TGAAGTTCATCTGCACCACAGGCAAGCTGCCCGTGCCCTGGCCCACCCTCGTGACCACCTTCGGCTACGGCCTGCAATGCTTCGCCCGCTACCCCGACCACATG<br>AAGCTGCACGACTTCTTCAAGTCCGCCATGCCCGAAGGCTACGTCCAGGAGCGCACCATCTTCTTCAAGGACGACGGCAACTACAAGACCCGCGCCGAGGTGAA<br>GTTCGAGGGCGACACCCTGGTGAACCGCATCGAGCTGAAGGGCATCGACTTCAAGGAGGACGGCAACATCCTGGGGCACAAGCTGGAGTACAACTACAACAGCC<br>ACAACGTCTATATCATGGCCGACAAGCAGAAGAACGGCATCAAGGTGAACTTCAAGATCCGCCACAACATCGAGGACGGCAGCGTGCAGCTCGCCGACCACTAC<br>CAGCAGAACACCCCCAATCGGCGACGGCCCCGTGCTGCTGCCCGACAACCACTACCTTAGCTACCAGTCCGCCCTGAGCAAAGACCCCAACGAGAAGCGCGATCA<br>CATGGTCCTGCTGGAGTTCGTGACCGCCGCCGGGATCACTCTCGGCATGGACGAGCTGTACAAGTAACTCGGTACCAAATTCCAGAAAAGAGGCCTCCCGAAAG<br>GGGGGCCTTTTTTCGTTTTGGTCCAATGGCGGCGCGCCATCGAATGAGGTGCTTCCTCGCTCACTGACTCGCTGCACGAGGCA
pAJM.1290	GGCCGCGTTGCTGGCGTTTTTCCATAGGCTCCGCCCCCCTGACGAGCATCACAAAAATCGACGCTCAAGTCAGAGGTGGCGAAACCCGACAGGACTATAAAGAT<br>ACCAGGCGTTTCCCCCTGGAAGCTCCCTCGTGCGCTCTCCTGTTCCGACCCTGCCGCTTACCGGATACCTGTCCGCCTTTCTCCCTTCGGGAAGCGTGGCGCTT<br>TCTCATAGCTCACGCTGTAGGTATCTCAGTTCGGTGTAGGTCGTTCGCTCCAAGCTGGGCTGTGTGCACGAACCCCCCGTTCAGCCCGACCGCTGCGCCTTATC<br>CGGTAACTATCGTCTTGAGTCCAACCCGGTAAGACACGACTTATCGCCACTGGCAGCAGCCACTGGTAACAGGATTAGCAGAGCGAGGTATGTAGGCGGTGCTA<br>CAGAGTTCTTGAAGTGGTGGCCTAACTACGGCTACACTAGAAGGACAGTATTTGGTATCTGCGCTCTGCTGAAGCCAGTTACCTTCGGAAAAAGAGTTGGTAGC<br>TCTTGATCCGGCAAACAAACCACCGCTGGTAGCGGTGGTTTTTTTGTTTGCAAGCAGCAGATTACGCGCAGAAAAAAAGGATCTCAAGAAGATCCTTTGATCTT<br>TTCTACGGGGTCTGACGCTCAGTGGAACGAAAACTCACGTTAAGGGATTTTGGTCATGAGATTATCAAAAAGGATCTTCACCTAGATCCTTTTAAATTAAAAAT<br>GAAGTTTCGGGCACGTAAGAGGTTCCAACTTTCACCATAATGAAATAAGATCACTACCGGGCGTATTTTTTGAGTTATCGAGATTTTCAGGAGCTAAGGAAGCT<br>AAAATGAGGGAAGCGGTGATCGCCGAAGTATCGACTCAACTATCAGAGGTAGTTGGCGTCATCGAGCGCCATCTCGAACCGACGTTGCTGGCCGTACATTTGTA<br>CGGCTCCGCAGTGGATGGCGGCCTGAAGCCACACAGTGATATTGATTTGCTGGTTACGGTGACCGTAAGGCTTGATGAAACAACGCGGCGAGCTTTGATCAACG<br>ACCTTTTGGAAACTTCGGCTTCCCCTGGAGAGAGCGAGATTCTCCGCGCTGTAGAAGTCACCATTGTTGTGCACGACGACATCATTCCGTGGCGTTATCCAGCT<br>AAGCGCGAACTGCAATTTGGAGAATGGCAGCGCAATGACATTCTTGCAGGTATCTTCGAGCCAGCCACGATCGACATTGATCTGGCTATCTTGCTGACAAAAGC<br>AAGAGAACATAGCGTTGCCTTGGTAGGTCCAGCGGCGGAGGAACTCTTTGATCCGGTTCCTGAACAGGATCTATTTGAGGCGCTAAATGAAACCTTAACGCTAT<br>GGAACTCGCCGCCCGACTGGGCTGGCGATGAGCGAAATGTAGTGCTTACGTTGTCCCGCATTTGGTACAGCGCAGTAACCGGCAAAATCGCGCCGAAGGATGTC<br>GCTGCCGACTGGGCAATGGAGCGCCTGCCGGCCCAGTATCAGCCCGTCATACTTGAAGCTAGACAGGCTTATCTTGGACAAGAAGAAGATCGCTTGGCCTCGCG<br>CACAGATCAGTTGGAAGAATTTGTCCACTACGTGAAAGGCGAGATCACCAAGGTAGTCGGCAAATAATACTAGCTCCGGCAAAAAAACGGGCAAGGTGTCACCA<br>CCCTGCCCTTTTTCTTTAAAACCGAAAAGATTACTTCGCGTTGAGATTTTGAGACACAAGGTCTCGCGCTGTACATCCTGCCCGCGTTTCTCTAGAATCGCACG<br>AAGACAGGTTTGTC**TTTACGGCTAGCTCAGCCCTAGGTATTATGCTAGC**TACTAGAGTCACACAGGAAAGTACTAGATGGCTTCCTCCGAAGACGTTATCAAAG<br>AGTTCATGCGTTTCAAAGTTCGTATGGAAGGTTCCGTTAACGGTCACGAGTTCGAAATCGAAGGTGAAGGTGAAGGTCGTCCGTACGAAGGTACCCAGACCGCT<br>AAACTGAAAGTTACCAAAGGTGGTCCGCTGCCGTTCGCTTGGGACATCCTGTCCCCGCAGTTCCAGTACGGTTCCAAAGCTTACGTTAAACACCCGGCTGACAT<br>CCCGGACTACCTGAAACTGTCCTTCCCGGAAGGTTTCAAATGGGAACGTGTTATGAACTTCGAAGACGGTGGTGTTGTTACCGTTACCCAGGACTCCTCCCTGC<br>AAGACGGTGAGTTCATCTACAAAGTTAAACTGCGTGGTACCAACTTCCCGTCCGACGGTCCGGTTATGCAGAAAAAAACCATGGGTTGGGAAGCTTCCACCGAA<br>CGTATGTACCCGGAAGACGGTGCTCTGAAAGGTGAAATCAAAATGCGTCTGAAACTGAAAGACGGTGGTCACTACGACGCTGAAGTTAAAACCACCTACATGGC<br>TAAAAAACCGGTTCAGCTGCCGGGTGCTTACAAAACCGACATCAAACTGGACATCACCTCCCACAACGAAGACTACACCATCGTTGAACAGTACGAACGTGCTG<br>AAGGTCGTCACTCCACCGGTGCTTAATAACCTAATTGGTAACGAATCAGACAATTGACGGATCCCAATGATGTGGGGTCCGATCTAACTAAAAAGGCCGCTCTG<br>CGGCCTTTTTTCTTTTCACTGGACAGTATTGAATGGACTCTGATCTGCCCGAGACCGGTAACTGTCAGACCAAGGAGCACATGGAACGGGTTGGCATGGATTGT<br>AGGCGCCGCCCTATACCTTGTCTGCCTCCCCGCGTTGCGTCGCGGTGCATGGAGCCGGGCCACCTCGACCTGAATGGAAGCCGGCGGCACCTCGCTAACGGATT<br>CACCACTCCAAGAATTGGAGCCAATCAATTCTTGCGGAGAACTGTGAATGCGCAAACCAACCCTTGGCAGAACATATCCATCGCGTCCGCCATCTCCAGCAGCC<br>GCACGCGGCGCATCTCGGGCAGCGTTGGGTCCTGGCCACGGGTGCGCATGATCGTGCTCCTGTCGTTGAGGACCCGGCTAGGCTGGCGGGGTTGCCTTACTGGT<br>TAGCAGAATGAATCACCGATACGCGAGCGAACGTGAAGCGACTGCTGCTGCAAAACGTCTGCGACCTGAGCAACAACATGAATGGTCTTCGGTTTCCGTGTTTC<br>GTAAAGTCTGGAAACGCGGAAGTCAGCGCCCTGCACCATTATGTTCCGGATCTGCATCGCAGGATGCTGCTGGCTACCCTGTGGAACACCTACATCTGTATTAA<br>CGAAGCGCTGGCATTGACCCTGAGTGATTTTTCTCTGGTCCCGCCGCATCCATACCGCCAGTTGTTTACCCTCACAACGTTCCAGTAACCGGGCATGTTCATCA<br>TCAGTAACCCGTATCGTGAGCATCCTCTCTCGTTTCATCGGTATCATTACCCCCATGAACAGAAATCCCCCTTACACGGAGGCATCAGTGACCAAACAGGAAAA<br>AACCGCCCTTAACATGGCCCGCTTTATCAGAAGCCAGACATTAACGCTTCTGGAGAAACTCAACGAGCTGGACGCGGATGAACAGGCAGACATCTGTGAATCGC<br>TTCACGACCACGCTGATGAGCTTTACCGCAGCTGCCTCGCGCGTTTCGGTGATGACGGTGAAAACCTCTGACACATGCAGCTCCCGGAGACGGTCACAGCTTGT<br>CTGTAAGCGGATGCCGGGAGCAGACAAGCCCGTCAGGGCGCGTCAGCGGGTGTTGGCGGGTGTCGGGGCGCAGCCATGACCCAGTCACGTAGCGATAGCGGAGT<br>GTATACTGGCTTAACTATGCGGCATCAGAGCAGATTGTACTGAGAGTGCACCATATGCGGTGTGAAATACCGCACAGATGCGTAAGGAGAAAATACCGCATCAG<br>GCGCTCTTCCGCTTCCTCGCTCACTGACTCGCTGCGCTCGGTCGTTCGGCTGCGGCGAGCGGTATCAGCTCACTCAAAGGCGGTAATACGGTTATCCACAGAAT<br>CAGGGGATAACGCAGGAAAGAACATGTGAGCAAAAGGCCAGCAAAAGGCCAGGAACCGTAAAAA
pAJM.1349	GACCTCAGCGCTAGCGGAGTGTATACTGGCTTACTATGTTGGCACTGATGAGGGTGTCAGTGAAGTGCTTCATGTGGCAGGAGAAAAAAGGCTGCACCGGTGCG<br>TCAGCAGAATATGTGATACAGGATATATTCCGCTTCCTCGCTCACTGACTCGCTACGCTCGGTCGTTCGACTGCGGCGAGCGGAAATGGCTTACGAACGGGGCG<br>GAGATTTCCTGGAAGATGCCAGGAAGATACTTAACAGGGAAGTGAGAGGGCCGCGGCAAAGCCGTTTTTCCATAGGCTCCGCCCCCCTGACAAGCATCACGAAA<br>TCTGACGCTCAAATCAGTGGTGGCGAAACCCGACAGGACTATAAAGATACCAGGCGTTTCCCCCTGGCGGCTCCCTCGTGCGCTCTCCTGTTCCTGCCTTTCGG<br>TTTACCGGTGTCATTCCGCTGTTATGGCCGCGTTTGTCTCATTCCACGCCTGACACTCAGTTCCGGGTAGGCAGTTCGCTCCAAGCTGGACTGTATGCACGAAC<br>CCCCCGTTCAGTCCGACCGCTGCGCCTTATCCGGTAACTATCGTCTTGAGTCCAACCCGGAAAGACATGCAAAAGCACCACTGGCAGCAGCCACTGGTAATTGA<br>TTTAGAGGAGTTAGTCTTGAAGTCATGCGCCGGTTAAGGCTAAACTGAAAGGACAAGTTTTGGTGACTGCGCTCCTCCAAGCCAGTTACCTCGGTTCAAAGAGT<br>TGGTAGCTCAGAGAACCTTCGAAAAACCGCCCTGCAAGGCGGTTTTTTCGTTTTCAGAGCAAGAGATTACGCGCAGACCAAAACGATCTCAAGAAGATCATCTT<br>ATTAAGGGGTCTGACGCTCAGTGGAACGAAAAATCAATCTAAAGTATATATGAGTAAACTTGGTCTGACAGTTACCTTAGAAAAACTCATCGAGCATCAAATGA<br>AACTGCAATTTATTCATATCAGGATTATCAATACCATATTTTTGAAAAAGCCGTTTCTGTAATGAAGGAGAAAACTCACCGAGGCAGTTCCATAGGATGGCAAG<br>ATCCTGGTATCGGTCTGCGATTCCGACTCGTCCAACATCAATACAACCTATTAATTTCCCCTCGTCAAAAATAAGGTTATCAAGTGAGAAATCACCATGAGTGA<br>CGACTGAATCCGGTGAGAATGGCAAAAGCTTATGCATTTCTTTCCAGACTTGTTCAACAGGCCAGCCATTACGCTCGTCATCAAAATCACTCGCATCAACCAAA<br>CCGTTATTCATTCGTGATTGCGCCTGAGCGAGACGAAATACGCGATCGCTGTTAAAAGGACAATTACAAACAGGAATCGAATGCAACCGGCGCAGGAACACTGC<br>CAGCGCATCAACAATATTTTCACCTGAATCAGGATATTCTTCTAATACCTGGAATGCTGTTTTCCCGGGGATCGCAGTGGTGAGTAACCATGCATCATCAGGAG<br>TACGGATAAAATGCTTGATGGTCGGAAGAGGCATAAATTCCGTCAGCCAGTTTAGTCTGACCATCTCATCTGTAACATCATTGGCAACGCTACCTTTGCCATGT<br>TTCAGAAACAACTCTGGCGCATCGGGCTTCCCATACAATCGATAGATTGTCGCACCTGATTGCCCGACATTATCGCGAGCCCATTTATACCCATATAAATCAGC<br>ATCCATGTTGGAATTTAATCGCGGCCTCGAGCAAGACGTTTCCCGTTGAATATGGCTCATAACACCCCTTGTATTACTGTTTATGTAAGCAGACAGTTTTATTG

	TTCATGATGATATATTTTTATCTTGTGCAATGTACATCAGAGATTTTGAGACACAACCAATTATTGAAGGCCTCCCTAACGGGGGGCCTTTTTTTGTTTCTGGT<br>CTCCCGCTTAACGATCGTTGGCTGAACTAGCAAATGAGATAGATTTCGGTGAACCCGGACCCTTGCTAGGCTCGAAGAACGCCAGCTGTCACCGGATGTGCTTT<br>CCGGTCTGATGAGTCCGTGAGGACGAAACAGCCTCTACAAATAATTTTGTTTAATACTAGAGAAAGAGGGGAAATACTAGATGGTGAGCAAGGGCGAGGAGCTG<br>TTCACCGGGGTGGTGCCCATCCTGGTCGAGCTGGACGGCGACGTAAACGGCCACAAGTTCAGCGTGTCCGGCGAGGGCGAGGGCGATGCCACCTACGGCAAGCT<br>GACCCTGAAGTTCATCTGCACCACAGGCAAGCTGCCCGTGCCCTGGCCCACCCTCGTGACCACCTTCGGCTACGGCCTGCAATGCTTCGCCCGCTACCCCGACC<br>ACATGAAGCTGCACGACTTCTTCAAGTCCGCCATGCCCGAAGGCTACGTCCAGGAGCGCACCATCTTCTTCAAGGACGACGGCAACTACAAGACCCGCGCCGAG<br>GTGAAGTTCGAGGGCGACACCCTGGTGAACCGCATCGAGCTGAAGGGCATCGACTTCAAGGAGGACGGCAACATCCTGGGGCACAAGCTGGAGTACAACTACAA<br>CAGCCACAACGTCTATATCATGGCCGACAAGCAGAAGAACGGCATCAAGGTGAACTTCAAGATCCGCCACAACATCGAGGACGGCAGCGTGCAGCTCGCCGACC<br>ACTACCAGCAGAACACCCCAATCGGCGACGGCCCCGTGCTGCTGCCCGACAACCACTACCTTAGCTACCAGTCCGCCCTGAGCAAAGACCCCAACGAGAAGCGC<br>GATCACATGGTCCTGCTGGAGTTCGTGACCGCCGCCGGGATCACTCTCGGCATGGACGAGCTGTACAAGTAACTCGGTACCAAATTCCAGAAAAGAGGCCTCCC<br>GAAAGGGGGGGCCTTTTTTCGTTTTGGTCCAATGGCGGCGCGCCATCGAATGAGGTGCTTCCTCGCTCACTGACTCGCTGCACGAGGCA
pAMK.106	TTTCCATAGGCTCCGCCCCCCTGACGAGCATCACAAAAATCGACGCTCAAGTCAGAGGTGGCGAAACCCGACAGGACTATAAAGATACCAGGCGTTTCCCCCTG<br>GAAGCTCCCTCGTGCGCTCTCCTGTTCCGACCCTGCCGCTTACCGGATACCTGTCCGCCTTTCTCCCTTCGGGAAGCGTGGCGCTTTCTCATAGCTCACGCTGT<br>AGGTATCTCAGTTCGGTGTAGGTCGTTCGCTCCAAGCTGGGCTGTGTGCACGAACCCCCCGTTCAGCCCGACCGCTGCGCCTTATCCGGTAACTATCGTCTTGA<br>GTCCAACCCGGTAAGACACGACTTATCGCCACTGGCAGCAGCCACTGGTAACAGGATTAGCAGAGCGAGGTATGTAGGCGGTGCTACAGAGTTCTTGAAGTGGT<br>GGCCTAACTACGGCTACACTAGAAGGACAGTATTTGGTATCTGCGCTCTGCTGAAGCCAGTTACCTTCGGAAAAAGAGTTGATAGCTCTTGATCCGGCAAACAA<br>ACCACCGCTGGTAGCGGTGGTTTTTTTGTTTGCAAGCAGCAGATTACGCGCAGAAAAAAAGGATCTCAAGAAGATCCTTTGATCTTTTCTACGGGGTCTGACGC<br>TCAGTGGAACGAAAACTCACGTTAAGGGATTTTGGTCATGAGATTATCAAAAAGGATCTTCACCTAGATCCTTTTAAATTAAAAATGAAGTTTTAAATCAATCT<br>AAAGTATATATGAGTAAACTTGGTCTGACAGTTACCAATGCTTAATCAGTGAGGCACCTATCTCAGCGATCTGTCTATTTCGTTCATCCATAGTTGCCTGACTC<br>CCCGTCGTGTAGATAACTACGATACGGGAGGGCTTACCATCTGGCCCCAGTGCTGCAATGATACCGCGGACCCACGCTCACCGGCTCCAGATTTATCAGCAAT<br>AAACCAGCCAGCCGGAAGGGCCGAGCGCAGAAGTGGTCCTGCAACTTTATCCGCCTCCATCCAGTCTATTAATTGTTGCCGGGAAGCTAGAGTAAGTAGTTCGC<br>CAGTTAATAGTTTGCGCAACGTTGTTGCCATTGCTGCAGGCATCGTGGTGTCACGCTCGTCGTTTGGTATGGCTTCATTCAGCTCCGGTTCCCAACGATCAAGG<br>CGAGTTACATGATCCCCCATGTTGTGCAAAAAAGCGGTTAGCTCCTTCGGTCCTCCGATCGTTGTCAGAAGTAAGTTGGCCGCAGTGTTATCACTCATGGTTAT<br>GGCAGCACTGCATAATTCTCTTACTGTCATGCCATCCGTAAGATGCTTTTCTGTGACTGGTGAGTACTCAACCAAGTCATTCTGAGAATAGTGTATGCGGCGAC<br>CGAGTTGCTCTTGCCCGGCGTCAACACGGGATAATACCGCGCCACATAGCAGAACTTTAAAAGTGCTCATCATTGGAAAACGTTCTTCGGGGCGAAAACTCTCA<br>AGGATCTTACCGCTGTTGAGATCCAGTTCGATGTAACCCACTCGTGCACCCAACTGATCTTCAGCATCTTTTACTTTCACCAGCGTTTCTGGGTGAGCAAAAAC<br>AGGAAGGCAAAATGCCGCAAAAAAGGGAATAAGGGCGACACGGAAATGTTGAATACTCATACTCTTCCTTTTTCAATATTATTGAAGCATTTATCAGGGTTATT<br>GTCTCATGAGCGGATACATATTTGAATGTATTTAGAAAAATAAACAAATAGGGGTTCCGCGCACATTTCCCCGAAAAGTGCCACCTTGCGCCGGATGACAACAG<br>AGGTGTTTTGATAGAAATGTCCGGTCAAGGTTGCCGTCAATTTGAATCCTTCTTGGAATGCCGGAAGAAAACATGGTACGACTTCTTTCAAGATTGCATGCAAC<br>AAGGCGGTTCGTTCACTCGCTTTGATTTAGCTATTGATGATAAAAAGACATACTTTTCTATACCAGAACTGTTAAAGAAAGCCCAAAAGGGCGAATGTATTTCA<br>CGATTTAGAAAATCTGATTTCAATGGCTCCTTTGATCTATCTGATGGCATTACAGGCGGGACGACAATATATTTCGGTTCTAAAAAGTCAGAAGCGTACCTTTG<br>CTTTTATGAGAAAAACTATGAACAAGCAGAAAAGTATAACATTCCGTTAGAAGAACTAGGCGATTGGAATCGTTATGAATTGCGGCTTAAAAATGAACGTGCAC<br>AAGTGGCGATAGATGCTTTATTGAAAACAAAGGATTTAACCTTAATCGCTATGCAGATCATTAATAACTATGTAAGGTTCGTTGATGCTGATGAAAACATCACT<br>AGAGAGCATTGGAAAACGAGCCTTTTTTGGTCTGATTTTATAGGCGATGTTGGGAGACTTCCTTTGTACGTCAAACCACAAAAAGACTTCTATCAGAAATCAAG<br>AAACTGGCTTCGTAACTCTTGTGCTCCCACTATGAAAATGGTGCTTGAAGCTGATGAACATTTAGGGAAAACCGATCTGTCAGATATGATTGCAGAAGCTGAAC<br>TGGCTGACAAACATAAAAAAATGTTAGATGTGTATATGGCTGATGTTGCTGACATGGTCGTTTAAAACCAAGCTGCTAAACCTTAATAAACACAGGTGATGCGA<br>TTAAACGTATGTGTCAACGGTACCGGAACGAAGTGAGGATAAGGAGTTGACATATTCAGCGCTACCCAAACCCTCTTGGCATTGAACAAACAACCGTTTGTTCC<br>TGCCTACCGGCGAGGGAGTCCCCCGATGATTCGGGGGAATAGGGGGATAGATTAACAGCAATCCAACGAGCTACCGTTGGTGGGTTTGGGTGAAACCCAAGAAC<br>TAACTTGGAGGGATAAGACTAGTGCCTCCTCATCCTCTTCATCCTCTTCGTCTTGGTAGCTTTTTAAATATGGCGCTTCATAGAGTAATTCTGTAAAGGTCCAA<br>TTCTCGTTTTCATACCTCGGTATAATCTTACCTATCACCTCAAATGGTTCTCTAGAGTCGACGAATTCAACAGCTAGAAACGCATGAGAAAGCCCCCGGAAGAT<br>CACCTTCCGGGGGCTTTTTTATTGCGCTCCTTGGCCCTCCATCCTTAGATAGTTCAGCCAAAAAACTTAAGACCGCCGGTCTTGTCCACTACCTTGCAGTAATG<br>CGGTGGACAGGATCGGCGGTTTTCTTTTCTCTTCTCAATCATAGGCAATACGATCGCATGTCCTAATACGACTCACTATAGGGGAATTGTGAGCGGATAACAAT<br>TATTCTGAGACCGGCGCGCCTTTACGGCTAGCTCAGTCCTAGGTACTATGCTAGCAAGGTAGCTGTCACCGGATGTGCTTTCCGGTCTGATGAGTCCGTGAGGA<br>CGAAACAGCCTCTACAAATAATTTTGTTTAATCACACAGGAAAGTACTAGATGAGCAAAGGAGAAGAACTTTTCACTGGAGTTGTCCCAATTCTTGTTGAATTA<br>GATGGTGATGTTAATGGGCACAAATTTTCTGTCCGTGGAGAGGGTGAAGGTGATGCTACAAACGGAAAACTCACCCTTAAATTTATTTGCACTACTGGAAAACT<br>ACCTGTTCCGTGGCCAACACTTGTCACTACTCTGACCTATGGTGTTCAATGCTTTTCCCGTTATCCGGATCACATGAAACGGCATGACTTTTTCAAGAGTGCCA<br>TGCCCGAAGGTTATGTACAGGAACGCACTATATCTTTCAAAGATGACGGGACCTACAAGACGCGTGCTGAAGTCAAGTTTGAAGGTGATACCCTTGTTAATCGT<br>ATCGAGTTAAAGGGTATTGATTTTAAAGAAGATGGAAACATTCTTGGACACAAACTCGAGTACAACTTTAACTCACACAATGTATACATCACGGCAGACAAACA<br>AAAGAATGGAATCAAAGCTAACTTCAAAATTCGCCACAACGTTGAAGATGGTTCCGTTCAACTAGCAGACCATTATCAACAAAATACTCCAATTGGCGATGGCC<br>CTGTCCTTTTACCAGACAACCATTACCTGTCGACACAATCTGTCCTTTCGAAAGATCCCAACGAAAAGCGTGACCACATGGTCCTTCTTGAGTTTGTAACTGCT<br>GCTGGGATTACACATGGCATGGATGAGCTCTACAAATAATACTAGAGCCAGGCATCAAATAAAACGAAAGGCTCAGTCGAAAGACTGGGCCTTTCGTTTTATCT<br>GTTGTTTGTCGGTGAACGCTCTCTACTAGAGTCACACTGGCTCACCTTCGGGTGGGCCTTTCTGCGTTTATAGGTCTCACTCGAAAAAAAAACCCCGCCCCTGA<br>CAGGGCGGGGTTTTTTTTTGGATTTCCTCCGACCTAATTTAATCCATTGTTTGTAAACATTTCATATCTAATTATATAACATAGACTCCTATTCTAACTACTT<br>ATTTTAAAGCAGTCTGGATTGTTTGGGTAATTCATAAAAAAATAAAAGAAAGAAGGAGGAATAGCAAATATTGGTACATTATTACAGCTATTTTGTAATCACGT<br>ACTCTCTTTGATAAAAAATTGGAGATTCCTTTACAAATATGCTCTTACGTGCTATTATTTAAGTATCTATTTAAAAGGAGTTAATAAATATGCGGCAAGGTATT<br>ATTAAATAAACTGTCAATTTGATAGCGGGAACAAATAATTGGATGTCCTTTTTTAGGAGGGCTTAGTTTTTTGTACCCAGTTTAAGAATACCTTTATCATGTGA<br>TTCTAAAGTATCCGGAGAATATCTGTATGCTTTGTATGCCTATGGTTATGCATAAAAATCCCAGTGATAAGAGTATTTATCACTGGGATTTTTATGCCCTTTTG<br>GGCTTTTGAATGGAGGAAAATCACATGAAAATTATTAATATTGGAGTTTTAGCTCATGTTGATGCAGGAAAAACTACCTTAACAGAAAGCTTATTATATAACAG<br>TGGAGCGATTACAGAATTAGGAAGCGTGGACAAAGGTACAACGAGGACGGATAATACGCTTTTAGAACGTCAGAGAGGAATTACAATTCAGACAGGAATAACCT<br>CTTTTCAGTGGGAAAATACGAAGGTGAACATCATAGACACGCCAGGACATATGGATTTCTTAGCAGAAGTATATCGTTCATTATCAGTTTTAGATGGGGCAATT<br>CTACTGATTTCTGCAAAAGATGGCGTACAAGCACAAACTCGTATATTATTTCATGCACTTAGGAAAATGGGGATTCCCACAATCTTTTTTATCAATAAGATTGA<br>CCAAAATGGAATTGATTTATCAACGGTTTATCAGGATATTAAAGAGAAACTTTCTGCCGAAATTGTAATCAAACAGAAGGTAGAACTGTATCCTAATGTGTGTG<br>TGACGAACTTTACCGAATCTGAACAATGGGATACGGTAATAGAGGGAAACGATGACCTTTTAGAGAAATATATGTCCGGTAAATCATTAGAAGCATTGGAACTC<br>GAACAAGAGGAAAGCATAAGATTTCAGAATTGTTCTCTGTTCCCTCTTTATCATGGAAGTGCAAAAAGTAATATAGGGATTGATAACCTTATAGAAGTTATTAC<br>TAATAAATTTTATTCATCAACACATCGAGGTCCGTCTGAACTTTGCGGAAATGTTTTCAAAATTGAATATACAAAAAAAAGACAACGTCTTGCATATATACGCC<br>TTTATAGTGGAGTACTACATTTACGAGATTCGGTTAGAGTATCAGAAAAAGAAAAAAATAAAAGTTACAGAAATGTATACTTCAATAAATGGTGAATTATGTAAG<br>ATTGATAGAGCTTATTCTGGAGAAATTGTTATTTTGCAAAATGAGTTTTTGAAGTTAAATAGTGTTCTTGGAGATACAAAACTATTGCCACAGAGAAAAAAGAT<br>TGAAAATCCGCACCCTCTACTACAAACAACTGTTGAACCGAGTAAACCTGAACAGAGAGAAATGTTGCTTGATGCCCTTTTGGAAATCTCAGATAGTGATCCGC<br>TTCTACGATATTACGTGGATTCTACGACACATGAAATTATACTTTCTTTCTTAGGGAAAGTACAAATGGAAGTGATTAGTGCACTGTTGCAAGAAAAGTATCAT<br>GTGGAGATAGAACTAAAAGAGCCTACAGTCATTTATATGGAGAGGCCGTTAAAAAATGCAGAATATACCATTCACATCGAAGTGCCGCCAAATCCTTTCTGGGC<br>TTCCATTGGTTTATCTGTATCACCGCTTCCGTTGGGAAGTGGAATGCAGTATGAGAGCTCGGTTTCTCTTGGATACTTAAATCAATCATTTCAAAATGCAGTTA<br>TGGAAGGGATACGCTATGGTTGTGAACAAGGATTGTATGGTTGGAATGTGACGGACTGTAAAATCTGTTTTAAGTATGGCTTATACTATAGCCCTGTTAGTACC<br>CCAGCAGATTTTCGGATGCTTGCTCCTATTGTATTGGAACAAGTCTTAAAAAAAGCTGGAACAGAATTGTTAGAGCCATATCTTAGTTTTAAAATTTATGCGCC<br>ACAGGAATATCTTTCACGAGCATACAACGATGCTCCTAAATATTGTGCGAACATCGTAGACACTCAATTGAAAAATAATGAGGTCATTCTTAGTGGAGAAATCC<br>CTGCTCGGTGTATTCAAGAATATCGTAGTGATTTAACTTTCTTTACAAATGGACGTAGTGTTTGTTTAACAGAGTTAAAAGGGTACCATGTTACTACCGGTGAA<br>CCTGTTTGCCAGCCCCGTCGTCCAAATAGTCGGATAGATAAAGTACGATATATGTTCAATAAAATAACTTAGTGTATTTTATGTTGTTATATAAATATGGTTTC<br>TTGTTAAATAAGATGAAATATTTTTTAATAAGATTTGAATTAAAGTGTCCGGCAATAGTTACCCTTATTATCAAGATAAGAAAGAAAAGGATTTTTCGCTACGC<br>TCAAATCCTTTAAAAAAACACAAAAGACCACATTTTTTAATGTGGTCTTTTATTCTTCAACTAAAGCACCCATTAGTTCAACAAACGAAAATTGGATAAAGTGG<br>GATATTTTTAAAATATATATTTATGTTACAGTAATATTGACTTTTAAAAAAGGATTGATTCTAATGAAGAAAGCAGACAAGTAAGCCTCCTAAATTCACTTTAG<br>ATAAAAATTTAGGAGGCATATCAAATGAACTTTAATAAAATTGATTTAGACAATTGGAAGAGAAAAGAGATATTTAATCATTATTTGAACCAACAAACGACTTT<br>TAGTATAACCACAGAAATTGATATTAGTGTTTTATACCGAAACATAAAACAAGAAGGATATAAATTTTACCCTGCATTTATTTTCTTAGTGACAAGGGTGATAA<br>ACTCAAATACAGCTTTTAGAACTGGTTACAATAGCGACGGAGAGTTAGGTTATTGGGATAAGTTAGAGCCACTTTATACAATTTTTGATGGTGTATCTAAAACA<br>TTCTCTGGTATTTGGACTCCTGTAAAGAATGACTTCAAAGAGTTTTATGATTTATACCTTTCTGATGTAGAGAAATATAATGGTTCGGGGAAATTGTTTCCCAA<br>AACACCTATACCTGAAAATGCTTTTTCTCTTTCTATTATTCCATGGACTTCATTTACTGGGTTTAACTTAAATATCAATAATAATAGTAATTACCTTCTACCCA<br>TTATTACAGCAGGAAAATTCATTAATAAAGGTAATTCAATATATTTACCGCTATCTTTACAGGTACATCATTCTGTTTGTGATGGTTATCATGCAGGATTGTTT<br>ATGAACTCTATTCAGGAATTGTCAGATAGGCCTAATGACTGGCTTTTATAATATGAGATAATGCCGACTGTACTTTTTACAGTCGGTTGTCTAATGTCACTAAC<br>CTGCCTTAGATAATTGGAAGAAGTACATCCGCAACTGTCCATACTCTGATGTCTAGAGGATCCCCGGGTACCGAGCTCGAATTCGCTATTTTATATCCATAGTT<br>GTTAATTAAATAAACTTAATTTAGTTTATTTATAGATTTCATTGGCTTCTAAATTTTTTATCTAGATAATAATTATTTTAGTTAATTTTATTCTAGATTATATA<br>TGATATGATCTTTCATTTCCATAAAACTAAAGTAAGTGTAAACCTATTCATTGTTTTAAAAATATCTCTTGCCAGTCACGTTACGTTATTAGTTATAGTTATTA<br>TAACATGTATTCACGAACGAAAATCGCCATTCGCCAGGGCTGCAGGAATTCGACTCTCTAGCTTGAGGCATCAAATAAAACGAAAGGCTCAGTCGAAAGACTGG<br>GCCTTTCGTTTTATCTGTTGTTTGTCGGTGAACGCTCTCCTGAGTAGGACAAATCCGCCGCTCTAGCTAAGCAGAAGGCCATCCTGACGGATGGCCTTTTTGCG<br>TTTCTACAAACTCTTGTTAACTCTAGAGCTGCCTGCCGCGTTTCGGTGATGAAGATCTTCCCGATGATTAATTAATTCAGAACGCTCGGTTGCCGCCGGGCGTT<br>TTTTATGCAGCAATGGCAAGAACGTTGCTCGAGGGTAAATGTGAGCACTCACAATTCATTTTGCAAAAGTATTTAGAAAGATTACTATATCCTCAAACAGGCGG<br>TAACCGGCCTCTTCATCGGGAATGCGCGCGACCTTCAACATCGCCGGCATGTCCCCCTGGCGGACGGGAAGTATCCAGCTCGAGGTCGGGCCGCGTTGCTGGCG<br>TT

pAMK.164	TTTCCATAGGCTCCGCCCCCCTGACGAGCATCACAAAAATCGACGCTCAAGTCAGAGGTGGCGAAACCCGACAGGACTATAAAGATACCAGGCGTTTCCCCCTG<br>GAAGCTCCCTCGTGCGCTCTCCTGTTCCGACCCTGCCGCTTACCGGATACCTGTCCGCCTTTCTCCCTTCGGGAAGCGTGGCGCTTTCTCATAGCTCACGCTGT<br>AGGTATCTCAGTTCGGTGTAGGTCGTTCGCTCCAAGCTGGGCTGTGTGCACGAACCCCCCGTTCAGCCCGACCGCTGCGCCTTATCCGGTAACTATCGTCTTGA<br>GTCCAACCCGGTAAGACACGACTTATCGCCACTGGCAGCAGCCACTGGTAACAGGATTAGCAGAGCGAGGTATGTAGGCGGTGCTACAGAGTTCTTGAAGTGGT<br>GGCCTAACTACGGCTACACTAGAAGGACAGTATTTGGTATCTGCGCTCTGCTGAAGCCAGTTACCTTCGGAAAAAGAGTTGATAGCTCTTGATCCGGCAAACAA<br>ACCACCGCTGGTAGCGGTGGTTTTTTTGTTTGCAAGCAGCAGATTACGCGCAGAAAAAAAGGATCTCAAGAAGATCCTTTGATCTTTTCTACGGGGTCTGACGC<br>TCAGTGGAACGAAAACTCACGTTAAGGGATTTTGGTCATGAGATTATCAAAAAGGATCTTCACCTAGATCCTTTTAAATTAAAAATGAAGTTTTAAATCAATCT<br>AAAGTATATATGAGTAAACTTGGTCTGACAGTTACCAATGCTTAATCAGTGAGGCACCTATCTCAGCGATCTGTCTATTTCGTTCATCCATAGTTGCCTGACTC<br>CCCGTCGTGTAGATAACTACGATACGGGAGGGCTTACCATCTGGCCCCAGTGCTGCAATGATACCGCGGGACCCACGCTCACCGGCTCCAGATTTATCAGCAAT<br>AAACCAGCCAGCCGGAAGGGCCGAGCGCAGAAGTGGTCCTGCAACTTTATCCGCCTCCATCCAGTCTATTAATTGTTGCCGGGAAGCTAGAGTAAGTAGTTCGC<br>CAGTTAATAGTTTGCGCAACGTTGTTGCCATTGCTGCAGGCATCGTGGTGTCACGCTCGTCGTTTGGTATGGCTTCATTCAGCTCCGGTTCCCAACGATCAAGG<br>CGAGTTACATGATCCCCCATGTTGTGCAAAAAAGCGGTTAGCTCCTTCGGTCCTCCGATCGTTGTCAGAAGTAAGTTGGCCGCAGTGTTATCACTCATGGTTAT<br>GGCAGCACTGCATAATTCTCTTACTGTCATGCCATCCGTAAGATGCTTTTCTGTGACTGGTGAGTACTCAACCAAGTCATTCTGAGAATAGTGTATGCGGCGAC<br>CGAGTTGCTCTTGCCCGGCGTCAACACGGGATAATACCGCGCCACATAGCAGAACTTTAAAAGTGCTCATCATTGGAAAACGTTCTTCGGGGCGAAAACTCTCA<br>AGGATCTTACCGCTGTTGAGATCCAGTTCGATGTAACCCACTCGTGCACCCAACTGATCTTCAGCATCTTTTACTTTCACCAGCGTTTCTGGGTGAGCAAAAAC<br>AGGAAGGCAAAATGCCGCAAAAAAGGGAATAAGGGCGACACGGAAATGTTGAATACTCATACTCTTCCTTTTTCAATATTATTGAAGCATTTATCAGGGTTATT<br>GTCTCATGAGCGGATACATATTTGAATGTATTTAGAAAAATAAACAAATAGGGGTTCCGCGCACATTTCCCCGAAAAGTGCCACCTTGCGCCGGATGACAACAG<br>AGGTGTTTTGATAGAAATGTCCGGTCAAGGTTGCCGTCAATTTGAATCCTTCTTGGAATGCCGGAAGAAAACATGGTACGACTTCTTTCAAGATTGCATGCAAC<br>AAGGCGGTTCGTTCACTCGCTTTGATTTAGCTATTGATGATAAAAAGACATACTTTTCTATACCAGAACTGTTAAAGAAAGCCCAAAAGGGCGAATGTATTTCA<br>CGATTTAGAAAATCTGATTTCAATGGCTCCTTTGATCTATCTGATGGCATTACAGGCGGGACGACAATATATTTCGGTTCTAAAAAGTCAGAAGCGTACCTTTG<br>CTTTTATGAGAAAAACTATGAACAAGCAGAAAAGTATAACATTCCGTTAGAAGAACTAGGCGATTGGAATCGTTATGAATTGCGGCTTAAAAATGAACGTGCAC<br>AAGTGGCGATAGATGCTTTATTGAAAACAAAGGATTTAACCTTAATCGCTATGCAGATCATTAATAACTATGTAAGGTTCGTTGATGCTGATGAAAACATCACT<br>AGAGAGCATTGGAAAACGAGCCTTTTTTGGTCTGATTTTATAGGCGATGTTGGGAGACTTCCTTTGTACGTCAAACCACAAAAAGACTTCTATCAGAAATCAAG<br>AAACTGGCTTCGTAACTCTTGTGCTCCCACTATGAAAATGGTGCTTGAAGCTGATGAACATTTAGGGAAAACCGATCTGTCAGATATGATTGCAGAAGCTGAAC<br>TGGCTGACAAACATAAAAAAATGTTAGATGTGTATATGGCTGATGTTGCTGACATGGTCGTTTAAAACCAAGCTGCTAAACCTTAATAAACACAGGTGATGCGA<br>TTAAACGTATGTGTCAACGGTACCGGAACGAAGTGAGGATAAGGAGTTGACATATTCAGCGCTACCCAAACCCTCTTGGCATTGAACAAACAACCGTTTGTTCC<br>TGCCTACCGGCGAGGGAGTCCCCCGATGATTCGGGGGAATAGGGGGATAGATTAACAGCAATCCAACGAGCTACCGTTGGTGGGTTTGGGTGAAACCCAAGAAC<br>TAACTTGGAGGGATAAGACTAGTGCCTCCTCATCCTCTTCATCCTCTTCGTCTTGGTAGCTTTTTAAATATGGCGCTTCATAGAGTAATTCTGTAAAGGTCCAA<br>TTCTCGTTTTCATACCTCGGTATAATCTTACCTATCACCTCAAATGGTTCTCTAGAGTCGACGAATTCAACAGCTAGAAACGCATGAGAAAGCCCCCGGAAGAT<br>CACCTTCCGGGGGCTTTTTTATTGCGCTCCTTGGCCCTCCATCCTTAGATAGTTCAGCCAAAAAACTTAAGACCGCCGGTCTTGTCCACTACCTTGCAGTAATG<br>CGGTGGACAGGATCGGCGGTTTTCTTTTCTCTTCTCAATCATAGGCAATACGATCGCATGTCC**TAATACGACTCACTATAGGGGAATTGTGAGCGGATAACAAT**<br>**T**ATTCCACTTGTGAATTAATCACAGTAATAGGAGGGTCGACATGCGTACTGGTAATGCAAACTAGTGGCAAAATATAAATGTCCATTAAATGCCACCCTGTACA<br>GGGTGGCATACAAATGCATTCTGCGAGGTTTATTTATGGATTATATATTGGGTCGCTATGTCAAAATTGCTCGATACGGGAGCGGTGGATTAGTTGGTGGAGGC<br>GGAAAGGAGCAATATGTTGAGGATTTAGCGCTTTGGGAAAATATTATAAAAACCGCATATTGTTTCATAACCCCATCGAGTTATACTGCAGCGTTGGAAACTGT<br>CAATATACCTGAAAAAGATTTCTCTAATTGCTTTAGGTTCCTTAAGGAAAATTTCTTTATTATACCTAGTGAGTATAATAACTCTACTGAGAATAATCGTTACT<br>CTCGTAACTTTTTACATTATCAAAGTTATGGTGCTAACCCTGTTCTTGTGCAAGATAAACTAAAAGATGCTAAGGTAGTAATTCTTGGTTGTGGGGGTATAGGA<br>AATCATGTATCTGTTATACTCGCAACGTCTGGTATTGGCGAAATTATACTAATTGATAATGATCAAATAGAAAATACAAATTTGACAAGGCAGGTTTTATTTTC<br>AGAGAATGATGTCGGTAAAAATAAAACAGAAGTTATAAAACGCGAGTTATTAAAAAGAAACTCTGAAATTTCGGTGTCTGAAATCGCTTTGAATATAAACGATT<br>ACACTGATCTTCATAAGGTACCAGAAGCTGATATATGGGTTGTTTCTGCAGATCATCCATTTAATCTGATCAACTGGGTTAATAAATATTGCGTTAGAGCAAAT<br>CAGCCCTATATAAATGCTGGGTATGTTAATGATATTGCAGTCTTTGGTCCATTATATGTTCCTGGTAAAACAGGATGTTATGAGTGCCAAAAAGTCGTGGCAGA<br>TCTATATGGTTCAGAAAAGGAAAACATTGATCATAAGATTAAGCTAATCAATAGCAGATTTAAACCAGCAACATTTGCACCTGTGAATAATGTTGCCGCTGCAT<br>TATGTGCGGCAGACGTAATTAAGTTCATTGGAAAATACTCCGAGCCATTATCACTTAATAAAAGAATAGGCATCTGGTCAGATGAAATAAAAATACATTCCCAA<br>AATATGGGCCGTTCGCCTGTTTGCTCTGTGTGTGGAAATAGAATGTGAATACTGCAAATATTTTATCTGTGAACTATGGAGCTTATAATGCTATACGTATGTTA<br>ATTGGTGTCTACCATGCAGCATTTCTTATATCTACAGGTGTCACTATATCTCAACTTGCTGTTTTACAAATCGTTTTTTCTGTGACAGTGCTGATTCTTGATTT<br>TCCTTTATCTGTCATTGCAGACAGATTTTACAGGAAACAGGTTGTAGTTAGTGGCGTATTTTTTACTATTTTATTTTACCCTCTTTGTATTCAGTCTCCTGATA<br>TGCTGGCATTGGTTTTTTCTGAAATATTTTATGCCATTGGTATTTGTTGTATATCTGGGGCTATAGAGGGATGGGTGTTAAGTGCTCTGGATGGTAGAAATGAA<br>AAATTTACCTTATGTGCTCATTTATGCGAAAGGGTATCTTCCTTTGGGAGTGTGATAACTGGTGCTGTTGGAATATTAGTTGTCTACTTCTATGATTCTTATAA<br>ATCTGGCTATATAGTATCGTTCTTCTTGATGTTTGTTGTCTTTATCGTTTTTTTATGTGTAAAAACTAACGGCAGTGGGGGTGAAAAAGAAGCACGAGATAGTA<br>TTATTAAACAAGCAAAAGAGAGTATTGGATTGATAACAGGAAGTTATAATGGGACATATTTTTTATCTATAACATGTATGTTTACACTTGGTATACAGGTAATT<br>TATCATTTTTGGCAGCCAATTATGTTATCTGGGGAAAGTATTGGCTCATTATCAAAAAGAGAGCTTATAACTTTACTGATGTGTCATATTGGTGCATTTTCAAT<br>GCAATATATAGCAAACTATTTTATGCCAAAGATAGATGTAAGATGTAAACTTTATATACTTGTTTGTACATTATGTTCATTTCTTAGTTCAATCGCCTGTATTT<br>TACTATTTATGTTAATAACAGATAATAATGTTGTTCTCGGTGTAGCTATTTTCTCTATAATCCATGGGATGACTTCAACAATACCTGTTGGAGCACAGAATATA<br>TTCATAAGCAGACTTAATGGCAATTATAAGAATATTATTTCGGGAGCTCTTGGTCTTGTTTCTCTTTCATGTCGTGTTGCTTCCATTTCTGTGTTGGGAGTTAT<br>ATCTGTATTACCAGGTAATATGCCTGTCACCACTTATATATTTATCTCTGCAATTGCATGTGTACTGTGTGGTTGTTTGTTCTTAATGTGGTCCTTTAAAAAGG<br>AGATGGTATGACGATTAAGCATGCTGAAGAATTGACGGAAGAAGTCATTTTAAATATGAATTACCCTGATTTTGTTGCTCTGATGCAACAGGATAACACTCCTC<br>CTGGAGCAGAGTACACCATAGACTACTGGATTAAGCATGGATGTATTAACAAAAAGTCTCACCTATTAGATTTAGCATGTTCCACGGGTTTTAGTTCACGTGAA<br>TGCTTCAAAAAAGAAGGTGCATCTGCAGAGGGTATTGATATATCTGAATCAGCTGTAATGGTCGCAAATGAAAAGGCAAAAAAGCTTAAGGCTAATAATTTATT<br>GAAATATTATGTAGCTGATGCTTGTGATTTACCATTTGAAGATAACACTTTTACACATGTTCTCGGTGGATGTAATTTTGCATTTATACAAAATCGGTTAATAG<br>CCTTGAATGAAACTCATCGATGTTTAAACCACATGGGAAGCATGTGCATTTCAAATTTTTATTACAGGCGTAAAATATCAGATAAACTTATCAATGATGTATAT<br>AATGCAATAAATTTCAGACCCAATCCTTTGTGGACTTTGGAATACTGGCATCAGTTTTTTTCTGAAAGATTTACTCTGGTTTCAGAAGAAAATCATGAGATGGA<br>ATCTCAAAGTGAAGATGAGTTAAAAGCCGATATTTATGATTACATTTTCAATAGAAATGAGTTTACAAAAGGTTTGAATGGTTCACTTCAGAATGTATTTTTTG<br>AAAGATTTTTAAAAATACGCAGACCATTAAATATCCAAAGAGATTATCAGGGAGTTACATTGCAAATATGGCGCAAAAAATAACACCATCTAAAATTGTTAAAC<br>ATGCCCGGGAGTTAATTATTAAGGGGATTGAATCGGGCGATAACTCATTTGTCATTTTTGATGTAGATGGAGCTCTGGAGCGTCTGGAACATTACAGGTGTCAA<br>CTTAAGAGTTTTTTCCCCAACAGCAGTATCGCATATTCATATAAGTCAAATAATCTGGCACAATGGTGCCAGATTATCTCAGGTAAAGGTTTGTATGCAGAGGT<br>GTGCTCTGTTGATGAAATGAACCTCGCTAAAAGAGACGGTTTTAACCGGATTGTTTTTGATGGCCCTTTAAAAAAAACGAGTGAACTATTAAAGGCAATAGAGA<br>TTGGGGCATTAATTGAAGTTGACAATATTGATGAATGTAAAAGACTTAATGAACTATGTAAATTGCATAAGTTGACATGTAGAATTCATTTGAGATTATCGCAT<br>TACTATGATGATAATTTATCAAGATTTGGCTTATCTGAAAGTGAGGCTATTAATTTACTGGAGATGTTGATCAGTAAGTCAGAGTATCTCATTTTAGACGGGTT<br>CCATTTACATGTTGGCTCTAATCTACCCAATGCGGAAAAAATATGTAAAGCTGTTATACAGTATCATGAGTTGATTCTTAGGTATATGCCAGATGATGGCACTC<br>TTAATCTTGGTAGTGGGATACCTGCAGATTCTTTCTCAGCATCCAGTGATAACCCAACTCCATGTCCGGAGGTATTTTTCTCATCGATATATGATACAATAAAA<br>AATTGCTTCGGTACTGTATGTGATAAATGGAACTATATTTTTGAACCTGGGAGACATCTGGTCGAAGACTTTGGCTACTTTATAGGGAAAGTTATTAGTACTAA<br>AAACAGGTATGGTGTTAAAGTTGCTCAAACTAACATTGGTATAAACTGGATACCTTCGATCAGAAATTGGGACCATTCATTTACATTGTTTCATAATCATAACC<br>ATATTTCAGATGATAAATCTGATGAGTATATTATTGCTGGATTCAATTGCTTTGAATGTGATTGCTTGTTTCCTTCAGTTATTCTTCCCAGTAACTTATCTGAT<br>TATTTATTTTCTGTTCGAGGGTGTGGTGCTTATGATATGCAAACAGGAAACCAGTGGACGCGTAATCTTTATGCTGTATATACCATAACAAATGATGTTGTTAA<br>TATATCAAGGATTCACAGAAGAGAACTTGATTTCCGGAAGTATGATGTATCTCTTACGCCATCAGGAATAAAAGTAAATGATGAGATTACATTATTGTATCCAG<br>CGTTAAAATACGCTGAAGAGTTATATTTGCTCATTAATCAAAACAAAATAAATTTCATTAAAAGCATGGCATGGCCAGCGTTTGTTAATAACATTTCTGATTCT<br>GTTTCTTTCATTGAGCAATCAATGATTGATAACCAGAATGAAAAAGCATTAATCCTCTTTATTAAATACAAAACTAAGATAGCTGGCGTTGTATCTTTTAATAT<br>TATTGACCATGCCAATAAAACAGCATATATTGGCTATTGGTTAGGCGCTAACTTTCAGGGAAAGGGGATTGTAACCAATGCTATAAATAAACTGATACAGGAGT<br>ATGGGGATTCGGGCGTTATAAAAAGATTTGTTATAAAATGTATTGTTGATAATAAAAAAAGTAATGCCACGGCATTGAGGTGTGGCTTCACCTTAGAGGGTGTT<br>CTGCAAAAAGCAGAAATACTCAACGGTGTATCATACGATCAAAATATTTATTCGAAAGTAATTGGTTAATGATGGTTCATTTTGACACTAAATGGAAAGGAAAA<br>ATCCTCATGATGATACAATCTCATCCGCTACTGGCCGCTCCCCTGGCAGTAGGAGATACAATTGGTTTCTTTTCATCATCTGCTCCGGCAACAGTTACTGCAAA<br>AAATCGTTTTTTTCGGGGAGTTGAGTTTCTTCAGAGAAAGGGATTTAAGCTGGTATCAGGGAAGCTTACCGGTAAAACAGATTTTTATCGTTCAGGTACTATTA<br>AAGAAAGAGCTCAAGAATTTAATGAGTTAGTCTACAATCCTGATATTACCTGTATAATGTCAACGATCGGTGGAGATAACAGTAATTCACTACTACCGTTTCTG<br>GACTATGATGCTATCATTGCAAACCCCAAAATTATCATAGGTTACTCAGATACAACTGCTTTATTAGCAGGAATATATGCAAAAACAGGGTTAATAACATTCTA<br>TGGGCCAGCTCTTATTCCTTCGTTTGGTGAGCATCCACCTCTTGTGGATATAACATATGAATCATTTATTAAAATACTAACAAGAAAACAATCAGGAATATATA<br>CCTACACATTACCTGAAAAGTGGAGTGATGAGAGCATAAACTGGAATGAAAACAAGATATTAAGGCCTAAGAAACTATATAAAAACAACTGTGCCTTTTATGGT<br>TCCGGAAAAGTTGAGGGGCGTGTAATTGGAGGAAATCTAAATACTTTGACAGGTATATGGGGGAGTGAATGGATGCCTGAAATTCGGAATGGAGATATATTGTT<br>TATTGAGGACAGTCGGAAAAGCATTGCAACAGTTGAACGATTATTCTCTATGCTAAAGCTTAATCGCGTGTTTGATAAAGTTAGTGCAATAATACTCGGGAAAC<br>ATGAGCTTTTTGATTGTGCAGGAAGTAAACGCAGACCATATGAAGTATTAACAGAGGTATTAGATGGGAAACAAATTCCTGTACTGGATGGATTTGATTGTTCA<br>CATACACATCCAATGCTAACTCTTCCACTTGGTGTAAAATTAGCTATTGACTTTGACAACAAAAATATATCTATAACAGAACAATATCTATCTACCGAGAAATA<br>ACTCGAAAAAAAAACCCCGCCCCTGACAGGGCGGGGTTTTTTTTTTGGATTTCCTCCGACCTAATTTAATCCATTGTTTGTAAACATTTCATATCTAATTATAT<br>AACATAGACTCCTATTCTAACTACTTATTTTAAAGCAGTCTGGATTGTTTGGGTAATTCATAAAAAAATAAAAGAAAGAAGGAGGAATAGCAAATATTGGTACA<br>TTATTACAGCTATTTTGTAATCACGTACTCTCTTTGATAAAAAATTGGAGATTCCTTTACAAATATGCTCTTACGTGCTATTATTTAAGTATCTATTTAAAAGG<br>AGTTAATAAATATGCGGCAAGGTATTATTAAATAAACTGTCAATTTGATAGCGGGAACAAATAATTGGATGTCCTTTTTTAGGAGGGCTTAGTTTTTTGTACCC<br>AGTTTAAGAATACCTTTATCATGTGATTCTAAAGTATCCGGAGAATATCTGTATGCTTTGTATGCCTATGGTTATGCATAAAAATCCCAGTGATAAGAGTATTT<br>ATCACTGGGATTTTTATGCCCTTTTGGGCTTTTGAATGGAGGAAAATCACATGAAAATTATTAATATTGGAGTTTTAGCTCATGTTGATGCAGGAAAAACTACC

	TTAACAGAAAGCTTATTATATAACAGTGGAGCGATTACAGAATTAGGAAGCGTGGACAAAGGTACAACGAGGACGGATAATACGCTTTTAGAACGTCAGAGAGG<br>AATTACAATTCAGACAGGAATAACCTCTTTTCAGTGGGAAAATACGAAGGTGAACATCATAGACACGCCAGGACATATGGATTTCTTAGCAGAAGTATATCGTT<br>CATTATCAGTTTTAGATGGGGCAATTCTACTGATTTCTGCAAAAGATGGCGTACAAGCACAAACTCGTATATTATTTCATGCACTTAGGAAAATGGGGATTCCC<br>ACAATCTTTTTTATCAATAAGATTGACCAAAATGGAATTGATTTATCAACGGTTTATCAGGATATTAAAGAGAAACTTTCTGCCGAAATTGTAATCAAACAGAA<br>GGTAGAACTGTATCCTAATGTGTGTGTGACGAACTTTACCGAATCTGAACAATGGGATACGGTAATAGAGGGAAACGATGACCTTTTAGAGAAATATATGTCCG<br>GTAAATCATTAGAAGCATTGGAACTCGAACAAGAGGAAAGCATAAGATTTCAGAATTGTTCTCTGTTCCCTCTTTATCATGGAAGTGCAAAAAGTAATATAGGG<br>ATTGATAACCTTATAGAAGTTATTACTAATAAATTTTATTCATCAACACATCGAGGTCCGTCTGAACTTTGCGGAAATGTTTTCAAAATTGAATATACAAAAAA<br>AAGACAACGTCTTGCATATATACGCCTTTATAGTGGAGTACTACATTTACGAGATTCGGTTAGAGTATCAGAAAAAGAAAAAATAAAAGTTACAGAAATGTATA<br>CTTCAATAAATGGTGAATTATGTAAGATTGATAGAGCTTATTCTGGAGAAATTGTTATTTTGCAAAATGAGTTTTTGAAGTTAAATAGTGTTCTTGGAGATACA<br>AAACTATTGCCACAGAGAAAAAAGATTGAAAATCCGCACCCTCTACTACAAACAACTGTTGAACCGAGTAAACCTGAACAGAGAGAAATGTTGCTTGATGCCCT<br>TTTGGAAATCTCAGATAGTGATCCGCTTCTACGATATTACGTGGATTCTACGACACATGAAATTATACTTTCTTTCTTAGGGAAAGTACAAATGGAAGTGATTA<br>GTGCACTGTTGCAAGAAAAGTATCATGTGGAGATAGAACTAAAAGAGCCTACAGTCATTTATATGGAGAGGCCGTTAAAAAATGCAGAATATACCATTCACATC<br>GAAGTGCCGCCAAATCCTTTCTGGGCTTCCATTGGTTTATCTGTATCACCGCTTCCGTTGGGAAGTGGAATGCAGTATGAGAGCTCGGTTTCTCTTGGATACTT<br>AAATCAATCATTTCAAAATGCAGTTATGGAAGGGATACGCTATGGTTGTGAACAAGGATTGTATGGTTGGAATGTGACGGACTGTAAAATCTGTTTTAAGTATG<br>GCTTATACTATAGCCCTGTTAGTACCCCAGCAGATTTTCGGATGCTTGCTCCTATTGTATTGGAACAAGTCTTAAAAAAAGCTGGAACAGAATTGTTAGAGCCA<br>TATCTTAGTTTTAAAATTTATGCGCCACAGGAATATCTTTCACGAGCATACAACGATGCTCCTAAATATTGTGCGAACATCGTAGACACTCAATTGAAAAATAA<br>TGAGGTCATTCTTAGTGGAGAAATCCCTGCTCGGTGTATTCAAGAATATCGTAGTGATTTAACTTTCTTTACAAATGGACGTAGTGTTTGTTTAACAGAGTTAA<br>AAGGGTACCATGTTACTACCGGTGAACCTGTTTGCCAGCCCCGTCGTCCAAATAGTCGGATAGATAAAGTACGATATATGTTCAATAAAATAACTTAGTGTATT<br>TTATGTTGTTATATAAATATGGTTTCTTGTTAAATAAGATGAAATATTTTTTAATAAGATTTGAATTAAAGTGTCCGGCAATAGTTACCCTTATTATCAAGATA<br>AGAAAGAAAAGGATTTTTCGCTACGCTCAAATCCTTTAAAAAAACACAAAAGACCACATTTTTTAATGTGGTCTTTTATTCTTCAACTAAAGCACCCATTAGTT<br>CAACAAACGAAAATTGGATAAAGTGGGATATTTTTAAAATATATATTTATGTTACAGTAATATTGACTTTTAAAAAAGGATTGATTCTAATGAAGAAAGCAGAC<br>AAGTAAGCCTCCTAAATTCACTTTAGATAAAAATTTAGGAGGCATATCAAATGAACTTTAATAAAATTGATTTAGACAATTGGAAGAGAAAAGAGATATTTAAT<br>CATTATTTGAACCAACAAACGACTTTTAGTATAACCACAGAAATTGATATTAGTGTTTTATACCGAAACATAAAACAAGAAGGATATAAATTTTACCCTGCATT<br>TATTTTCTTAGTGACAAGGGTGATAAACTCAAATACAGCTTTTAGAACTGGTTACAATAGCGACGGAGAGTTAGGTTATTGGGATAAGTTAGAGCCACTTTATA<br>CAATTTTTGATGGTGTATCTAAAACATTCTCTGGTATTTGGACTCCTGTAAAGAATGACTTCAAAGAGTTTTATGATTTATACCTTTCTGATGTAGAGAAATAT<br>AATGGTTCGGGGAAATTGTTTCCCAAAACACCTATACCTGAAAATGCTTTTTCTCTTTCTATTATTCCATGGACTTCATTTACTGGGTTTAACTTAAATATCAA<br>TAATAATAGTAATTACCTTCTACCCATTATTACAGCAGGAAAATTCATTAATAAAGGTAATTCAATATATTTACCGCTATCTTTACAGGTACATCATTCTGTTT<br>GTGATGGTTATCATGCAGGATTGTTTATGAACTCTATTCAGGAATTGTCAGATAGGCCTAATGACTGGCTTTTATAATATGAGATAATGCCGACTGTACTTTTT<br>ACAGTCGGTTGTCTAATGTCACTAACCTGCCTTAGATAATTGGAAGAAGTACATCCGCAACTGTCCATACTCTGATGTCTAGAGGATCCCCGGGTACCGAGCTC<br>GAATTCGCTATTTTATATCCATAGTTGTTAATTAAATAAACTTAATTTAGTTTATTTATAGATTTCATTGGCTTCTAAATTTTTTATCTAGATAATAATTATTT<br>TAGTTAATTTTATTCTAGATTATATATGATATGATCTTTCATTTCCATAAAACTAAAGTAAGTGTAAACCTATTCATTGTTTTAAAAATATCTCTTGCCAGTCA<br>CGTTACGTTATTAGTTATAGTTATTATAACATGTATTCACGAACGAAAATCGCCATTCGCCAGGGCTGCAGGAATTCGACTCTCTAGCTTGAGGCATCAAATAA<br>AACGAAAGGCTCAGTCGAAAGACTGGGCCTTTCGTTTTATCTGTTGTTTGTCGGTGAACGCTCTCCTGAGTAGGACAAATCCGCCGCTCTAGCTAAGCAGAAGG<br>CCATCCTGACGGATGGCCTTTTTGCGTTTCTACAAACTCTTGTTAACTCTAGAGCTGCCTGCCGCGTTTCGGTGATGAAGATCTTCCCGATGATTAATTAATTC<br>AGAACGCTCGGTTGCCGCCGGGCGTTTTTTATGCAGCAATGGCAAGAACGTTGCTCGAGGGTAAATGTGAGCACTCACAATTCATTTTGCAAAAGTATTTAGAA<br>AGATTACTATATCCTCAAACAGGCGGTAACCGGCCTCTTCATCGGGAATGCGCGCGACCTTCAACATCGCCGGCATGTCCCCCTGGCGGACGGGAAGTATCCAG<br>CTCGAGGTCGGGCCGCGTTGCTGGCGTT
pCAV.5063	TGATTCGCACGGGCCCATGGCTAATTCCCATGTCAGCCGTTAAGTGTTCCTGTGTCACTCAAAATTGCTTTGAGAGGCTCTAAGGGCTTCTCAGTGCGTTACAT<br>CCCTGGCTTGTTGTCCACAACCGTTAAACCTTAAAAGCTTTAAAAGCCTTATATATTCTTTTTTTTCTTATAAAACTTAAAACCTTAGAGGCTATTTAAGTTGC<br>TGATTTATATTAATTTTATTGTTCAAACATGAGAGCTTAGTACGTGAAACATGAGAGCTTAGTACGTTAGCCATGAGAGCTTAGTACGTTAGCCATGAGGGTTT<br>AGTTCGTTAAACATGAGAGCTTAGTACGTTAAACATGAGAGCTTAGTACGTGAAACATGAGAGCTTAGTACGTACTATCAACAGGTTGAACTGCTGATCTTCAG<br>ATCCTCTACGCCGGACGCATCGTGGCCGGATCTTCTTGTGCTTGGATTCTCACCAATAAAAAACGCCCGGCGGCAACCGAGCGTTCTGAACAAATCCAACATTT<br>AATAAAAAAAGGGCGGTCGCAAGATCGCCCTTTTTTACGTATGACACCTCGTCTCAACCAAAGCAATCAACCAGTGAAAAATGGCGCCCATCGGCGCCATTTTT<br>TTATGGTTCCTTATCATCTGGCGAATCGGATTGACAGCTTATCATCGATAAACTGTAATGCGGTAGTTTATCACAGTTAAATTGCTAACGCAGTCAGGCACCGT<br>GTATGAAATCTAACAATGCGCTCATCGTCATCCTCGGCACCGTCACCCTGGATGCTGTAGGCATAGGCTTGGTTATGCCGGTACTGCCGGGCCTCTTGCGGGAT<br>ATCGTCCATTCCGACAGCATCGCCAGTCACTATGGCGTGCTGCTAGCGCTATATGCGTTGATGCAATTTCTATGCGCACCCGTTCTCGGAGCACTGTCCGACCG<br>CTTTGGCCGCCGCCCAGTCCTGCTCGCTTCGCTACTTGGAGCCACTATCGACTACGCGATCATGGCGACCACACCCGTCCTGTGGATTCTCTACGCCGGACGCA<br>TCGTGGCCGGCATCACCGGCGCCACAGGTGCGGTTGCTGGCGCCTATATCGCCGACATCACCGATGGGGAAGATCGGGCTCGCCACTTCGGGCTCATGAGCGCT<br>TGTTTCGGCGTGGGTATGGTGGCAGGCCCCGTGGCCGGGGGACTGTTGGGCGCCATCTCCCTGCACGCACCATTCCTTGCGGCGGCGGTGCTCAACGGCCTCAA<br>CCTACTACTGGGCTGCTTCCTAATGCAGGAGTCGCATAAGGGAGAGCGCCGTCCGATGCCCTTGAGAGCCTTCAACCCAGTCAGCTCCTTCCGGTGGGCGCGGG<br>GCATGACCATTGTGGCCGCACTTATGACTGTCTTCTTTATCATGCAACTCGTAGGACAGGTGCCGGCAGCGCTCTGGGTCATTTTCGGCGAGGACCGCTTTCGC<br>TGGAGCGCGACGATGATCGGCCTGTCGCTTGCGGTATTCGGAATCTTGCACGCCCTCGCTCAAGCCTTCGTCACTGGTCCCGCCACCAAACGTTTCGGCGAGAA<br>GCAGGCCATTATCGCCGGCATGGCGGCCGACGCGCTGGGCTACGTCTTGCTGGCGTTCGCGACGCGAGGCTGGATGGCCTTCCCCATTATGATTCTTCTCGCTT<br>CCGGCGGCATCGGGATGCCCGCGTTGCAGGCCATGCTGTCCAGGCAGGTAGATGACGACCATCAGGGACAGCTTCAAGGATCGCTCGCGGCTCTTACCAGCCTA<br>ACTTCGATCACTGGACCGCTGATCGTCACGGCGATTTATGCCGCCTCGGCGAGCACATGGAACGGGTTGGCATGGATTGTAGGCGCCGCCCTATACCTTGTCTG<br>CCTCCCCGCGTTGCGTCGCGGTGCATGGAGCCGGGCCACCTCGACCTGAGACAATTGTCTAATTAATTGCGGACCCTAGAGGTCCCCTTTTTTATTTTAAAAAT<br>TTTTTCACAAAACGGTTTACAAGCATAAAATCTCTGAGAAGTTCCTATTCCGAAGTTCCTATTCTCTAGAAAGTATAGGAACTTCGGATCCGAATTCGAGCTCC<br>GTCGACACTAGTGCGGCCGCACTCGAGGTCC**TTGACGGCTAGCTCAGTCCTAGGTACAGTGCTAGC**GCGAAAGCAACTAAGGAGAATCTATATGAAAAACATAA<br>ATGCCGACGACACATACAGAATAATTAATAAAATTAAAGCTTTTAGAAGCAATAATGATATTAATCAATGCTTATCTGATATGACTAAAATGGTACATTGTGAA<br>TATTATTTACTCGCGATCATTTATCCTCATTCTATGGTTAAATCTGATATTTCAATCCTAGATAATTACCCTAAAAAATGGAGGCAATATTATGATGACGCTAA<br>TTTAATAAAATATGATCCTATAGTAGATTATTCTAACTCCAATCATTCACCAATTAATTGGAATATATTTGAAAACAATGCTGTAAATAAAAAATCTCCAAATG<br>TAATTAAAGAAGCGAAAACATCAGGTCTTATCACTGGGTTTAGTTTCCCTATTCATATGGCTAACAATGGCTTCGGAATGCTTAGTTTTGCATATTCAGAAAAA<br>GACAACTATATAGATAGTTTATTTTTACATGCGTGTATGAACATACCATTAATTGTTCCTTCTCTAGTTGATAATTATCGAAAAATAAATATAGCAAATAATAA<br>ATCAAACAACGATTTAACCAAAAGAGAAAAAGAATGTTTAGCGTGGGCATGCGAAGGAAAAAGCTCTTGGGATATTTCAAAAATATTAGGATGCAGTGAGCGTA<br>CTGTCACTTTCCATTTAACCAATGCGCAAATGAAACTCAATACAACAAACCGCTGCCAAAGTATTTCTAAAGCAATTTTAACAGGAGCAATTGATTGCCCATAC<br>TTTAAAAATTGATAACCGCAGCACCTAAGGAGGCTAATTCATGATTGAGAATACCTATAGCGAAAAGTTCGAGTCCGCGTTCGAACAGATCAAAGCGGCGGCCA<br>ACGTGGATGCCGCCATCCGTATTCTCCAGGCGGAATATAACCTCGATTTCGTCACCTACCATCTCGCCCAGACAATCGCGAGCAAGATCGATTCGCCCTTCGTG<br>CGCACCACCTATCCGGATGCCTGGGTTTCCCGTTACCTCCTCAACTGCTATGTGAAGGTCGATCCGATCATCAAGCAGGGCTTCGAACGCCAGCTGCCCTTCGA<br>CTGGAGCGAGGTCGAACCGACGCCGGAGGCCTATGCCATGCTGGTCGACGCCCAGAAACACGGCATCGATGACAATGGCTACTCCATCCCCGTCGCCGACAAGG<br>CGCAGCGCCGCGCCCTGCTGTCGCTGAATGCCCATATACCGGCCGACGAATGGACCGAGCTCGTGCGCCGCTACCGCAACGAGTGGATCGAGATCGCCCATCTG<br>ATCCACCGCAAGGCCGTATATGAGCTGCATGGCGAAAACGATCCGGTGCCGGCATTGTCGCCGCGCGAGATCGAGTGTCTGCACTGGACCGCCCTCGGCAAGGA<br>TTACAAGGATATTTCGGTCATCCTGGGCATATCAGAGCATACCACACGCGATTACCTGAAAACCGCCCGCTTCAGGCTCGGCTGCACCACGATCTCGGCCGCCG<br>CGTCGCGGGCTGTTCAATTGTGCATCATCAATCCCTATAGGATCCGCATGACGCGACGTAATTGGTAATAGTAAGGGCAGGTAAGGAGGTATTTTTATGGCCTT<br>GGTTGACGGTTTTCTTGAGCTGGAACGCTCAAGTGGAAAATTGGAGTTGAGCGCCATCCTGCAGAAGATGGCGAGCGACCTCGGATTCTCGAAGATCCTGTTCG<br>GCCTGTTGCCTAAGGACAGCCAGGACTACGAGAACGCCTTCATCGTCAGCAACTACCCGGCCGCCTGGCGCGAGCATTACGACCGAACTGGCTACGCGCGGGTC<br>GACCCGACGGTCAGTCACTGTACCCAGAGCGTACTGCCGATTTTCTGGGAACCGCCCATCTACCAGACGCGAAAGCAGCACGAGTTCTTCGAGGAAGCCTTGGC<br>CGCTGGCCTGGTGTATGGACTGACCATGCCGCTGCATGGTGCTCGCGGCGAACTCGGCGCGCTGAGCCTCAGCGTGGAAGCGGAAAACCGGGCCGAGGCCAACC<br>GTTTCATGGAGTCGGTCCTGCCGACCCTGTGGATGCTCAAGGACTACGCACTGCAGAGCGGTGTCGGAATGGCCTTCGAACATCCGATCAGCAAACCGGTGGTT<br>CTGACCAGCCGAGAGAAGGAAGTGTTACAGTGGTGCGCCATCGGCAAGACCAGCTGGGAGATATCGGTTATCTGCAACTGCTCGGAAGCCAATGTGAACTTCCA<br>TATGGGAAATATTCGGCGGAAGTTCGGTGTGACCTCCCGCCGCGTAGCGGCCATTATGGCCGTTAATTTGGGTCTTATTACTCTCTGATAATGGCAACAATAAG<br>GAGGTAGTTTCTATGATTGTGGGTGAAGATCAGCTGTGGGGTCGTCGTACACTGGAATTTGTTGATAGCGTTGAACGTCTGGAAGCACCGGCACTGATTAGCCG<br>TTTTGAAAGCCTGATTGCAAGCTGTGGTTTTACCGCCTATATCATGGCAGGTCTGCCGAGCCGTAATGCCGGTCTGCCGGAACTGACCCTGGCAAATGGTTGGC<br>CTCGTGATTGGTTTGATCTGTATGTTAGCGAAAACTTTAGCGCAGTTGATCCGGTTCCGCGTTATGGTGCAACCACTGTTCATCCGTTTGTTTGGAGTGATGCA<br>CCGTATGATCGTGACCGTGATCAGGCAGCACATCGTGTTATGACCCGTGCAGCAGAATTTGGTCTGGTTGAAGGTTATTGTATTCCGCTGCATTACGATGATGG<br>TAGCGCAGCAATTAGTATGGCAGGTGAAGATCCTGATCTGAGTCCGGCAGCCCGTGGTGTAATGCAGCTGGTTAGCATTTATGCACATAGCCGTCTGCGTGTAC<br>TGAGCCGTCCGAAACCGATTCGTCGTAATCGTCTGACACCGCGTGAATGTGAAATTCTGCAGTGGGCAGCACAGGGTAAAACCGCATGGGAAATTAGCGTTATT<br>CTGTGTATTACCGAACGCACCGTTAAATTTCATCTGATTGAAGCAGCACGTAAACTGGATGCAGCAAATCGTACCGCAGCAGTTGCAAAAGCACTGACACTGGG<br>TCTGATTCGTCTGTGATAAGGATCCTAATAATTGGTAACGAATCAGACAATTGACGGCTCGAGGGAGTAGCATAGGGTTTGCAGAATCCCTGCTTCGTCCATTT<br>GACAGGCACATTATGCATCGATGATAAGCTGTCAAACATGAGCAGATCCTCTACGCCGGACGCATCGTGGCCGGCATCACCGGCGCCACAGGTGCGGTTGCTGG<br>CGCCTATATCGCCGACATCACCGATGGGGAAGATCGGGCTCGCCACTTCGGGCTCATGAGCAAATATTTTATCTGGCGGCGCGCCATCGAATGGTGAAAAACCT<br>TTCGCGGTATGGCATGATAGCGCCCGGAAGAGAGTCAATTCATGGAGGTGAATATGAAACCAGTAACGTTATACGATGTCGCAGAGTATGCCGGTGTCTCTTAT<br>ATGACCGTTTCCCGCGTGGTGAACCAGGCCAGCCACGTTTCTGCGAAAACGCGGGAAAAAGTGGAAGCGGCGATGGTGGAGCTGAATTACATTCCCAACCGCGT<br>GGCACAACAACTGGCGGGCAAACAGTCGTTGCTGATTGGCGTTGCCACCTCCAGTCTGGCCCTGCACGCGCCGTCGCAAATTGTCGCGGCGATTAAATCTCGCG<br>CCGATCAACTGGGTGCCAGCGTGGTGGTGTCGATGGTAGAACGAAGCGGCGTCGAAGCCTGTAAAGCGGCGGTGCACAATCTTCTCGCGCAACGCGTCAGTGGG<br>CTGATCATTAACTATCCGCTGGATGACCAGGATGCCATTGCTGTGGAAGCTGCCTGCACTAATGTTCCGGCGTTATTTCTTGATGTCTCTGACCAGACACCCAT

	CAACAGTATTATTTACTCCCATGAGGACGGTACGCGACTGGGCGTGGAGCATCTGGTCGCATTGGGTCACCAGCAAATCGCGCTGTTAGCGGGCCCATTAAGTT<br>CTGTCTCGGCGCGTCTGCGTCTGGCTGGCTGGCATAAATATCTCACTCGCAATCAAATTCAGCCGATAGCGGAACGGGAAGGCGACTGGAGTGCCATGTCCGGT<br>TTTCAACAAACCATGCAAATGCTGAATGAGGGCATCGTTCCCACTGCGATGCTGGTTGCCAACGATCAGATGGCGCTGGGCGCAATGCGCGCCATTACCGAGTC<br>CGGGCTGCGCGTTGGTGCGGATATCTCGGTAGTGGGATACGACGATACCGAAGATAGCTCATGTTATATCCCGCCGTTAACCACCATCAAACAGGATTTTCGCC<br>TGCTGGGGCAAACCAGCGTGGACCGCTTGCTGCAACTCTCTCAGGGCCAGGCGGTGAAGGGCAATCAGCTGTTGCCAGTCTCACTGGTGAAAAGAAAAACCACC<br>CTGGCGCCCAATACGCAAACCGCCTCTCCCCGCGCGTTGGCCGATTCATTAATGCAGCTGGCACGACAGGTTTCCCGACTGGAAAGCGGGCAGTGAGTAATAAT<br>CAAGGTGGCAAAAAATGTCCAGATTAGATAAAAGTAAAGTGATTAACAGCGCATTAGAGCTGCTTAATGAGGTCGGAATCGAAGGTTTAACAACCCGTAAACTC<br>GCCCAGAAGCTAGGTGTAGAGCAGCCTACATTGTATTGGCATGTAAAAAATAAGCGGGCTTTGCTCGACGCCTTAGCCATTGAGATGTTAGATAGGCACCATAC<br>TCACTTTTGCCCTTTAGAAGGGGAAAGCTGGCAAGATTTTTTACGTAATAACGCTAAAAGTTTTAGATGTGCTTTACTAAGTCATCGCGATGGAGCAAAAGTAC<br>ATTTAGGTACACGGCCTACAGAAAAACAGTATGAAACTCTCGAAAATCAATTAGCCTTTTTATGCCAACAAGGTTTTTCACTAGAGAATGCATTATATGCACTC<br>AGCGCTGTGGGGCATTTTACTTTAGGTTGCGTATTGGAAGATCAAGAGCATCAAGTCGCTAAAGAAGAAAGGGAAACACCTACTACTGATAGTATGCCGCCATT<br>ATTACGACAAGCTATCGAATTATTTGATCACCAAGGTGCAGAGCCAGCCTTCTTATTCGGCCTTGAATTGATCATATGCGGATTAGAAAAACAACTTAAATGTG<br>AAAGTGGGTCCTAAGGATCCCAATGATGTGGGGTCCCGCTTTCAAGGAGGTAAATATGGCTGAAGCGCAAAATGATCCCCTGCTGCCGGGATACTCGTTTAATG<br>CCCATCTGGTGGCGGGTTTAACGCCGATTGAGGCCAACGGTTATCTCGATTTTTTTATCGACCGACCGCTGGGAATGAAAGGTTATATTCTCAATCTCACCATT<br>CGCGGTCAGGGGGTGGTGAAAAATCAGGGACGAGAATTTGTTTGCCGACCGGGTGATATTTTGCTGTTCCCGCCAGGAGAGATTCATCACTACGGTCGTCATCC<br>GGAGGCTCGCGAATGGTATCACCAGTGGGTTTACTTTCGTCCGCGCGCCTACTGGCATGAATGGCTTAACTGGCCGTCAATATTTGCCAATACGGGGTTCTTTC<br>GCCCGGATGAAGCGCACCAGCCGCATTTCAGCGACTTTTTTGGGCAAATCATTAACGCCGGGCAAGGGGAAGGGCGCTATTCGGAGCTGCTGGCGATAAATCTG<br>CTTGAGCAATTGTTACTGCGGCGCATGCTAGCGATTAACGGATCGCTCCATCCACCGATGGATAATCGGGTACGCGAGGCTTGTCAGTACATCAGCGATCACCT<br>GGCAGACAGCAATTTTGATATCGCCAGCGTCGCACAGCATGTTTGCTTGTCGCCGTCGCGTCTGTCACATCTTTTCCGCCAGCAGTTAGGGATTAGCGTCTTAA<br>GCTGGCGCGAGGACCAACGTATCAGCCAGGCGAAGCTGCTTTTGAGCACCACCCGGATGCCTATCGCCACCGTCGGTCGCAATGTTGGTTTTGACGATCAACTC<br>TATTTCTCGCGGGTATTTAAAAAATGCACCGGGGCCAGCCCGAGCGAGTTCCGTGCCGGTTTGGAAGAAAAAGTGAATGATGTAGCCGTCAAGTTGTCATGATA<br>AGATCCTATTCCAGCGGGATTAAAGAGGAGCGATTAAGCATGGTTACTATCAATACGGAATCTGCTTTAACGCCACGTTCTTTGCGGGATACGCGGCGTATGAA<br>TATGTTTGTTTCGGTAGCTGCTGCGGTCGCAGGATTGTTATTTGGTCTTGATATCGGCGTAATCGCCGGAGCGTTGCCGTTCATTACCGATCACTTTGTGCTGA<br>CCAGTCGTTTGCAGGAATGGGTGGTTAGTAGCATGATGCTCGGTGCAGCAATTGGTGCGCTGTTTAATGGTTGGCTGTCGTTCCGCCTGGGGCGTAAATACAGC<br>CTGATGGCGGGGGCCATCCTGTTTGTACTCGGTTCTATAGGGTCCGCTTTTGCGACCAGCGTAGAGATGTTAATCGCCGCTCGTGTGGTGCTGGGCATTGCTGT<br>CGGGATCGCGTCTTACACCGCTCCTCTGTATCTTTCTGAAATGGCAAGTGAAAACGTTCGCGGTAAGATGATCAGTATGTACCAGTTGATGGTCACACTCGGCA<br>TCGTGCTGGCGTTTTTATCCGATACAGCGTTCAGTTATAGCGGTAACTGGCGCGCAATGTTGGGGGTTCTTGCTTTACCAGCAGTTCTGCTGATTATTCTGGTA<br>GTATTTCTGCCAAATAGCCCGCGCTGGCTGGCGGAAAAGGGGCGTCATATTGAGGCGGAAGAAGTATTGCGTATGCTGCGCGATACGTCGGAAAAAGCGCGAGA<br>AGAACTCAACGAAATTCGTGAAAGCCTGAAGTTAAAACAGGGCGGTTGGGCACTGTTTAAGATCAACCGTAACGTCCGTCGTGCTGTGTTTCTCGGTATGTTGT<br>TGCAGGCGATGCAGCAGTTTACCGGTATGAACATCATCATGTACTACGCGCCGCGTATCTTCAAAATGGCGGGCTTTACGACCACAGAACAACAGATGATTGCG<br>ACTCTGGTCGTAGGGCTGACCTTTATGTTCGCCACCTTTATTGCGGTGTTTACGGTAGATAAAGCAGGGCGTAAACCGGCTCTGAAAATTGGTTTCAGCGTGAT<br>GGCGTTAGGCACTCTGGTGCTGGGCTATTGCCTGATGCAGTTTGATAACGGTACGGCTTCCAGTGGCTTGTCCTGGCTCTCTGTTGGCATGACGATGATGTGTA<br>TTGCCGGTTATGCGATGAGCGCCGCGCCAGTGGTGTGGATCCTGTGCTCTGAAATTCAGCCGCTGAAATGCCGCGATTTCGGTATTACCTGTTCGACCACCACG<br>AACTGGGTGTCGAATATGATTATCGGCGCGACCTTCCTGACACTGCTTGATAGCATTGGCGCTGCCGGTACGTTCTGGCTCTACACTGCGCTGAACATTGCGTT<br>TGTGGGCATTACTTTCTGGCTCATTCCGGAAACCAAAAATGTCACGCTGGAACATATCGAACGCAAACTGATGGCAGGCGAGAAGTTGAGAAATATCGGCGTCT<br>GAGATGTGGGGTCCGATCTAACTAAAAAGGCCGCTCTGCGGCCTTTTTTCTTTTCACTCGCTCCCTAATACGCAAGTCGATAACTCTCCTGGGAGCGTTGACAA<br>CTTGCGCACCCTGATCTGGGAAACACAGAAAAAAGCCCGCACCTGACAGTGCGGGCTTTTTTTTTCGACCAAAGGCTCGGTACCAAATTCCAGAAAAGACACCC<br>GAAAGGGTGTTTTTTCGTTTTGGTCCAGGCGGTAATACGGTTATCCACAGAATCAGGGGATAACGCAGGAAAGAACATGTGAGCAAAAGGCCAGCAAAAGGCCA<br>GGAACCGTAAAAA
pCHR.4	GACCTCAGCGCTAGCGGAGTGTATACTGGCTTACTATGTTGGCACTGATGAGGGTGTCAGTGAAGTGCTTCATGTGGCAGGAGAAAAAAGGCTGCACCGGTGCG<br>TCAGCAGAATATGTGATACAGGATATATTCCGCTTCCTCGCTCACTGACTCGCTACGCTCGGTCGTTCGACTGCGGCGAGCGGAAATGGCTTACGAACGGGGCG<br>GAGATTTCCTGGAAGATGCCAGGAAGATACTTAACAGGGAAGTGAGAGGGCCGCGGCAAAGCCGTTTTTCCATAGGCTCCGCCCCCCTGACAAGCATCACGAAA<br>TCTGACGCTCAAATCAGTGGTGGCGAAACCCGACAGGACTATAAAGATACCAGGCGTTTCCCCTGGCGGCTCCCTCGTGCGCTCTCCTGTTCCTGCCTTTCGGT<br>TTACCGGTGTCATTCCGCTGTTATGGCCGCGTTTGTCTCATTCCACGCCTGACACTCAGTTCCGGGTAGGCAGTTCGCTCCAAGCTGGACTGTATGCACGAACC<br>CCCCGTTCAGTCCGACCGCTGCGCCTTATCCGGTAACTATCGTCTTGAGTCCAACCCGGAAAGACATGCAAAAGCACCACTGGCAGCAGCCACTGGTAATTGAT<br>TTAGAGGAGTTAGTCTTGAAGTCATGCGCCGGTTAAGGCTAAACTGAAAGGACAAGTTTTGGTGACTGCGCTCCTCCAAGCCAGTTACCTCGGTTCAAAGAGTT<br>GGTAGCTCAGAGAACCTTCGAAAAACCGCCCTGCAAGGCGGTTTTTTCGTTTTCAGAGCAAGAGATTACGCGCAGACCAAAACGATCTCAAGAAGATCATCTTA<br>TTAAGGGGTCTGACGCTCAGTGGAACGAAAAATCAATCTAAAGTATATATGAGTAAACTTGGTCTGACAGTTACCTTAGAAAAACTCATCGAGCATCAAATGAA<br>ACTGCAATTTATTCATATCAGGATTATCAATACCATATTTTTGAAAAAGCCGTTTCTGTAATGAAGGAGAAAACTCACCGAGGCAGTTCCATAGGATGGCAAGA<br>TCCTGGTATCGGTCTGCGATTCCGACTCGTCCAACATCAATACAACCTATTAATTTCCCCTCGTCAAAAATAAGGTTATCAAGTGAGAAATCACCATGAGTGAC<br>GACTGAATCCGGTGAGAATGGCAAAAGCTTATGCATTTCTTTCCAGACTTGTTCAACAGGCCAGCCATTACGCTCGTCATCAAAATCACTCGCATCAACCAAAC<br>CGTTATTCATTCGTGATTGCGCCTGAGCGAGACGAAATACGCGATCGCTGTTAAAAGGACAATTACAAACAGGAATCGAATGCAACCGGCGCAGGAACACTGCC<br>AGCGCATCAACAATATTTTCACCTGAATCAGGATATTCTTCTAATACCTGGAATGCTGTTTTCCCGGGGATCGCAGTGGTGAGTAACCATGCATCATCAGGAGT<br>ACGGATAAAATGCTTGATGGTCGGAAGAGGCATAAATTCCGTCAGCCAGTTTAGTCTGACCATCTCATCTGTAACATCATTGGCAACGCTACCTTTGCCATGTT<br>TCAGAAACAACTCTGGCGCATCGGGCTTCCCATACAATCGATAGATTGTCGCACCTGATTGCCCGACATTATCGCGAGCCCATTTATACCCATATAAATCAGCA<br>TCCATGTTGGAATTTAATCGCGGCCTCGAGCAAGACGTTTCCCGTTGAATATGGCTCATAACACCCCTTGTATTACTGTTTATGTAAGCAGACAGTTTTATTGT<br>TCATGATGATATATTTTTATCTTGTGCAATGTACATCAGAGATTTTGAGACACAACCAATTATTGAAGGCCTCCCTAACGGGGGGCCTTTTTTTGTTTCTGGTC<br>TCCCGCTTAACGATCGTTGGCTG**TGTTGACAATTAATCATCGGCTCGTATAATGTGTGGAATTGTGAGCGCTCACAATT**AGCTGTCACCGGATGTGCTTTCCGG<br>TCTGATGAGTCCGTGAGGACGAAACAGCCTCTACAAATAATTTTGTTTAATGGTTCCAAAGCCAGATACTAAGGAGGTCCCTATGCAGGTTCATGTTATTCGTC<br>GTGAAAATCGTGCACTGTATGCAGGTCTGCTGGAAAAATACTTTCGTATTCGCCATCAGATCTATGTTGTTGAACGTGGTTGGAAAGAACTGGATCGTCCGGAT<br>GGTCGTGAAATTGATCAGTTTGATACCGAAGATGCAGTTTATCTGCTGGGTGTTGATAACGATGATATTGTTGCAGGTATGCGTATGGTTCCGACCACCAGTCC<br>GACCCTGCTGAGTGATGTTTTTCCGCAGCTGGCACTGGCAGGTCCGGTTCGTCGTCCTGATGCATATGAACTGAGCCGTATTTTTGTTGTTCCGCGTAAACGTG<br>GTGAACATGGTGGTCCGCGTGCAGAAGCAGTTATTCAGGCAGCAGCAATGGAATATGGTCTGAGCATTGGTCTGAGTGCATTTACCATTGTTCTGGAAACCTGG<br>TGGCTGCCTCGTCTGGTTGATCAGGGCTGGAAAGCAAAACCGCTGGGTCTGCCGCAGGATATTAATGGTTTTAGCACCACCGCAGTTATTGTGGATGTTGATGA<br>TGATGCATGGGTGGGTATTTGTAATCGTCGTAGCGTTCCGGGTCCGACACTGGAATGGCGTGGTCTGGAAGCAATTCGTCGTCATAGCCTGCCGGAATTTCAGG<br>TTATTAGCTAAAAGGAGCGCTCTCCAGCCCATGGAGAAGGACACGAAACAAGTCGACATCATTTTTCGTTCGAAGCTCCCCGACATTTACATCCCCAACCATCT<br>TCCGCTTCACTCGTACTGTTTTGAAAACATTTCCGAATTCAGCAGCCGGCCCTGTCTCATTAACGGCGCAAATAAGCAGATTTACACCTACGCGGATGTTGAAC<br>TTAATTCGCGCAAAGTCGCCGCCGGCCTCCATAAGCAGGGTATCCAGCCCAAAGATACGATCATGATCCTGCTTCCCAACTCGCCCGAATTCGTTTTCGCGTTT<br>ATTGGCGCGTCGTACCTGGGCGCAATCAGCACGATGGCCAACCCCCTTTTTACCCCCGCCGAGGTGGTTAAGCAAGCCAAGGCGAGCTCCGCCAAGATTATTGT<br>CACGCAGGCATGCCACGTCAATAAAGTTAAGGATTACGCATTTGAAAATGATGTGAAGATTATCTGCATCGACAGCGCCCCCGAGGGCTGCCTGCACTTCTCGG<br>TCCTTACGCAGGCCAATGAGCACGACATTCCCGAGGTGGAGATTCAGCCCGATGACGTCGTCGCGCTCCCCTATAGCAGCGGCACCACGGGTCTGCCCAAAGGT<br>GTCATGCTCACCCACAAAGGCCTCGTGACCAGCGTGGCACAACAGGTGGACGGCGAAAACCCCAACCTTTACATCCATTCCGAGGACGTTATGCTGTGTGTGCT<br>CCCCCTGTTTCACATTTACTCCCTTAACTCGGTCCTTCTTTGCGGCCTCCGTGTTGGCGCAGCAATCCTTATTATGCAAAAGTTCGATATTGTCAGCTTCCTGG<br>AGCTCATTCAACGCTACAAAGTCACGATTGGCCCCTTCGTCCCCCCGATTGTGCTCGCGATTGCGAAGTCCCCCATGGTGGATGACTACGATCTCAGCAGCGTC<br>CGTACGGTCATGTCCGGCGCGGCACCGCTCGGTAAAGAACTGGAGGATACGGTCCGGGCCAAATTCCCGAATGCGAAGCTGGGTCAGGGTTACGGCATGACCGA<br>GGCAGGTCCCGTGCTCGCCATGTGCCTGGCATTCGCGAAGGAGCCCTTCGAGATCAAGAGCGGTGCGTGCGGCACGGTCGTTCGGAACGCGGAAATGAAAATCG<br>TGGACCCCAAAACCGGCAATTCCCTTCCCCGCAACCAATCCGGCGAAATTTGCATCCGGGGTGACCAGATTATGAAGGGCTATCTTAATGACCCGGAAGCCACG<br>GCGCGGACGATTGACAAAGAAGGTTGGCTGTATACCGGTGACATTGGTTACATCGACGATGACGACGAGCTTTTTATTGTGGACCGGCTCAAAGAACTCATCAA<br>ATATAAAGGCTTTCAAGTTGCGCCCGCGGAGCTGGAGGCACTGCTTCTTAACCATCCCAACATCTCGGATGCCGCGGTCGTGCCGATGAAGGATGAACAAGCGG<br>GTGAAGTGCCGGTCGCGTTTGTGGTTCGGAGCAACGGCTCCACGATTACGGAGGATGAGGTTAAGGATTTTATTTCCAAGCAGGTTATCTTTTATAAACGGATT<br>AAACGCGTTTTCTTCGTCGATGCCATCCCCAAATCCCCCAGCGGCAAGATCCTTCGGAAAGATCTTCGTGCGAAACTGGCAGCAGGTCTTCCGAATTAGCTCGG<br>TACCAAATTCCAGAAAAGAGGCCTCCCGAAAGGGGGGCCTTTTTTCGTTTTGGTCCAATG**GCGGCGCGCCATCGAATGGCGCAAAACCTTTCGCGGTATGGCAT**<br>**GATAGCGCCCGGAAGAGAGTCAATTCAGGGTGGTGAAT**ATGAAACCAGTAACGTTATACGATGTCGCAGAGTATGCCGGTGTCTCTTATATGACCGTTTCCCGC<br>GTGGTGAACCAGGCCAGCCACGTTTCTGCGAAAACGCGGGAAAAAGTGGAAGCGGCGATGGTGGAGCTGAATTACATTCCCAACCGCGTGGCACAACAACTGGC<br>GGGCAAACAGTCGTTGCTGATTGGCGTTGCCACCTCCAGTCTGGCCCTGCACGCGCCGTCGCAAATTGTCGCGGCGATTAAATCTCGCGCCGATCAACTGGGTG<br>CCAGCGTGGTGGTGTCGATGGTAGAACGAAGCGGCGTCGAAGCCTGTAAAGCGGCGGTGCACAATCTTCTCGCGCAACGCGTCAGTGGGCTGATCATTAACTAT<br>CCGCTGGATGACCAGGATGCCATTGCTGTGGAAGCTGCCTGCACTAATGTTCCGGCGTTATTTCTTGATGTCTCTGACCAGACACCCATCAACAGTATTATTTA<br>CTCCCATGAGGACGGTACGCGACTGGGCGTGGAGCATCTGGTCGCATTGGGTCACCAGCAAATCGCGCTGTTAGCGGGCCCATTAAGTTCTGTCTCGGCGCGTC<br>TGCGTCTGGCTGGCTGGCATAAATATCTCACTCGCAATCAAATTCAGCCGATAGCGGAACGGGAAGGCGACTGGAGTGCCATGTCCGGTTTTCAACAAACCATG<br>CAAATGCTGAATGAGGGCATCGTTCCCACTGCGATGCTGGTTGCCAACGATCAGATGGCGCTGGGCGCAATGCGCGCCATTACCGAGTCCGGGCTGCGCGTTGG<br>TGCGGATATCTCGGTAGTGGGATACGACGATACCGAAGATAGCTCATGTTATATCCCGCCGTTAACCACCATCAAACAGGATTTTCGCCTGCTGGGGCAAACCA<br>GCGTGGACCGCTTGCTGCAACTCTCTCAGGGCCAGGCGGTGAAGGGCAATCAGCTGTTGCCAGTCTCACTGGTGAAAAGAAAAACCACCCTGGCGCCCAATACG<br>CAAACCGCCTCTCCCCGCGCGTTGGCCGATTCATTAATGCAGCTGGCACGACAGGTTTCCCGACTGGAAAGCGGGCAGTGATAAGGATCCTAATTGGTAACGAA<br>TCAGACAATTGACGGCTCGAGGGAGTAGCATAGGGTTTGCAGAATCCCTGCTTCGTCCATTTGACAGGCACATTATGCATCGATGATAAGCTGTCAAACATGAG<br>CAGATCCTCTACGCCGGACGCATCGTGGCCGGCATCACCGGCGCCACAGGTGCGGTTGCTGGCGCCTATATCGCCGACATCACCGATGGGGAAGATCGGGCTCG

	CCACTTCGGGCTCATGAGCAAATATTTTATCTGAGGTGCTTCCTCGCTCACTGACTCGCTGCACGAGGCA
pJSBS.RPU [B]	GACCTCAGCGCTAGCGGAGTGTATACTGGCTTACTATGTTGGCACTGATGAGGGTGTCAGTGAAGTGCTTCATGTGGCAGGAGAAAAAAGGCTGCACCGGTGCG<br>TCAGCAGAATATGTGATACAGGATATATTCCGCTTCCTCGCTCACTGACTCGCTACGCTCGGTCGTTCGACTGCGGCGAGCGGAAATGGCTTACGAACGGGGCG<br>GAGATTTCCTGGAAGATGCCAGGAAGATACTTAACAGGGAAGTGAGAGGGCCGCGGCAAAGCCGTTTTTCCATAGGCTCCGCCCCCCTGACAAGCATCACGAAA<br>TCTGACGCTCAAATCAGTGGTGGCGAAACCCGACAGGACTATAAAGATACCAGGCGTTTCCCCCTGGCGGCTCCCTCGTGCGCTCTCCTGTTCCTGCCTTTCGG<br>TTTACCGGTGTCATTCCGCTGTTATGGCCGCGTTTGTCTCATTCCACGCCTGACACTCAGTTCCGGGTAGGCAGTTCGCTCCAAGCTGGACTGTATGCACGAAC<br>CCCCCGTTCAGTCCGACCGCTGCGCCTTATCCGGTAACTATCGTCTTGAGTCCAACCCGGAAAGACATGCAAAAGCACCACTGGCAGCAGCCACTGGTAATTGA<br>TTTAGAGGAGTTAGTCTTGAAGTCATGCGCCGGTTAAGGCTAAACTGAAAGGACAAGTTTTGGTGACTGCGCTCCTCCAAGCCAGTTACCTCGGTTCAAAGAGT<br>TGGTAGCTCAGAGAACCTTCGAAAAACCGCCCTGCAAGGCGGTTTTTTCGTTTTCAGAGCAAGAGATTACGCGCAGACCAAAACGATCTCAAGAAGATCATCTT<br>ATTAAGGGGTCTGACGCTCAGTGGAACGAAAAATCAATCTAAAGTATATATGAGTAAACTTGGTCTGACAGTTACCTTAGAAAAACTCATCGAGCATCAAATGA<br>AACTGCAATTTATTCATATCAGGATTATCAATACCATATTTTTGAAAAAGCCGTTTCTGTAATGAAGGAGAAAACTCACCGAGGCAGTTCCATAGGATGGCAAG<br>ATCCTGGTATCGGTCTGCGATTCCGACTCGTCCAACATCAATACAACCTATTAATTTCCCCTCGTCAAAAATAAGGTTATCAAGTGAGAAATCACCATGAGTGA<br>CGACTGAATCCGGTGAGAATGGCAAAAGCTTATGCATTTCTTTCCAGACTTGTTCAACAGGCCAGCCATTACGCTCGTCATCAAAATCACTCGCATCAACCAAA<br>CCGTTATTCATTCGTGATTGCGCCTGAGCGAGACGAAATACGCGATCGCTGTTAAAAGGACAATTACAAACAGGAATCGAATGCAACCGGCGCAGGAACACTGC<br>CAGCGCATCAACAATATTTTCACCTGAATCAGGATATTCTTCTAATACCTGGAATGCTGTTTTCCCGGGGATCGCAGTGGTGAGTAACCATGCATCATCAGGAG<br>TACGGATAAAATGCTTGATGGTCGGAAGAGGCATAAATTCCGTCAGCCAGTTTAGTCTGACCATCTCATCTGTAACATCATTGGCAACGCTACCTTTGCCATGT<br>TTCAGAAACAACTCTGGCGCATCGGGCTTCCCATACAATCGATAGATTGTCGCACCTGATTGCCCGACATTATCGCGAGCCCATTTATACCCATATAAATCAGC<br>ATCCATGTTGGAATTTAATCGCGGCCTGGAGCAAGACGTTTCCCGTTGAATATGGCTCATAACACCCCTTGTATTACTGTTTATGTAAGCAGACAGTTTTATTG<br>TTCATGATGATATATTTTTATCTTGTGCAATGTACATCAGAGATTTTGAGACACAACCAATTATTGAAGGCCTCCCTAACGGGGGGCCTTTTTTTGTTTCTGGT<br>CTCCCGCTTGATAAGTCCCTAACT**TTTACAGCTAGCTCAGTCCTAGGTATTATGCTAGC**CTGAAGCTGTCACCGGATGTGCTTTCCGGTCTGATGAGTCCGTGA<br>GGACGAAACAGCCTCTACAAATAATTTTGTTTAATACTAGAGAAAGAGGGGAAATACTAGATGGTGAGCAAGGGCGAGGAGCTGTTCACCGGGGTGGTGCCCAT<br>CCTGGTCGAGCTGGACGGCGACGTAAACGGCCACAAGTTCAGCGTGTCCGGCGAGGGCGAGGGCGATGCCACCTACGGCAAGCTGACCCTGAAGTTCATCTGCA<br>CCACAGGCAAGCTGCCCGTGCCCTGGCCCACCCTCGTGACCACCTTCGGCTACGGCCTGCAATGCTTCGCCCGCTACCCCGACCACATGAAGCTGCACGACTTC<br>TTCAAGTCCGCCATGCCCGAAGGCTACGTCCAGGAGCGCACCATCTTCTTCAAGGACGACGGCAACTACAAGACCCGCGCCGAGGTGAAGTTCGAGGGCGACAC<br>CCTGGTGAACCGCATCGAGCTGAAGGGCATCGACTTCAAGGAGGACGGCAACATCCTGGGGCACAAGCTGGAGTACAACTACAACAGCCACAACGTCTATATCA<br>TGGCCGACAAGCAGAAGAACGGCATCAAGGTGAACTTCAAGATCCGCCACAACATCGAGGACGGCAGCGTGCAGCTCGCCGACCACTACCAGCAGAACACCCCA<br>ATCGGCGACGGCCCCGTGCTGCTGCCCGACAACCACTACCTTAGCTACCAGTCCGCCCTGAGCAAAGACCCCAACGAGAAGCGCGATCACATGGTCCTGCTGGA<br>GTTCGTGACCGCCGCCGGGATCACTCTCGGCATGGACGAGCTGTACAAGTAACTCGGTACCAAATTCCAGAAAAGAGGCCTCCCGAAAGGGGGGCCTTTTTTCG<br>TTTTGGTCCGATCCTCTACGCCGGACGCATCGTGGCCGGCATCACCGGCGCCACAGGTGCGGTTGCTGGCGCCTATATCGCCGACATCACCGATGGGGAAGATC<br>GGGCTCGCCACTTCGGGCTCATGAGCAAATATTTTATCTGAGGTGCTTCCTCGCTCACTGACTCGCTGCACGAGGCA
pJYP.053 [C]	CCGGTTGTCAGCCGTTAAGTGTTCCTGTGTCACTCAAAATTGCTTTGAGAGGCTCTAAGGGCTTCTCAGTGCGTTACATCCCTGGCTTGTTGTCCACAACCGTT<br>AAACCTTAAAAGCTTTAAAAGCCTTATATATTCTTTTTTTTCTTATAAAACTTAAAACCTTAGAGGCTATTTAAGTTGCTGATTTATATTAATTTTATTGTTCA<br>AACATGAGAGCTTAGTACGTGAAACATGAGAGCTTAGTACGTTAGCCATGAGAGCTTAGTACGTTAGCCATGAGGGTTTAGTTCGTTAAACATGAGAGCTTAGT<br>ACGTTAAACTTGAGAGCTTAGTACGTGAAACATGAGAGCTTAGTACGTACTATCAACAGGTTGAACTGCCCATGTTCTTTCCTGCGTTATCAGAGCTTATCGGC<br>CAGCCTCGCAGAGCAGGATTCCCGTTGAGCACCGCCAGGTGCGAATAAGGGACAGTGAAGAAGGAACACCCGCTCGCGGGTGGGCCTACTTCACCTATCCTGCC<br>CGGCTGACGCCGTTGGATACACCAAGGAAAGTCTACACGAACCCTTTGGCAAAATCCTGTATATCGTGCGAAAAAGGATGGATATACCGAAAAAATCGCTATAA<br>TGACCCCGAAGCAGGGTTATGCAGCGGAAAGTATACCTTAACATGTTCTTTCCTGCGTTATCCCCTGATTCTGTGGATAACCGTATTACCGCCTTTGAGTGAGC<br>TGATACCGCTCGCCGCAGCCGAACGACCGAGCGCAGCGAGTCAGTGAGCGAGGAAGCGGAAGAGCGCCCAATACGCAAACCGCCTCTCCCCGCGCGTTGGCCGA<br>TTCATTAATGCAGCTGGCACGACAGGTTTCCCGACTGGAAAGCGGGCAGTGAGCGCAACGCAATTAATGTGAGTTAGCTCACTCATTAGGCACCCCAGGCTTTA<br>CACTTTATGCTTCCGGCTCGTATGTTGTGTGGAATTGTGAGCGGATAACAATTTCACACAGGAAACAGCTATGACCATGATTACGAATTCGGCTTGCGGCCGCT<br>TCTAGAG**GGCGCGCCTTTACAGCTAGCTCAGTCCTAGGTATTATGCTAGCCAGTT**AGCTGTCACCGGATGTGCTTTCCGGTCTGATGAGTCCGTGAGGACGAAA<br>CAGCCTCTACAAATAATTTTGTTTAATCACACAGGAAAGTACTAGATGCCGATTGCACCGGAATTTCTGAGCCTGGCATATCCGGGTCAAGAATTTCCGGCATA<br>TCTGTATGGTCGTGCAAGCCGTGATCCGAAACGTAAAGGTCGTAGCGTTCAGAGCCAGCTGGATGAAGGTCGTGCCACCTGTCTGGATGCAGGTTGGCCGATTG<br>CCGGTGAATTTAAAGATGTTGATCGTAGCGCAAGCGCATATGCACGTCGTACCCGTGATGAATTTGAAGAAATGATTGCAGGTATTCAGGCAGGCGAATGTCGT<br>ATTCTGGTTGCATTTGAAGCAAGCCGTTATTATCGTGATCTGGAAGCATATGTTCGTCTGCGTCGTGTTTGTCGTGAAGCCGGTGTTCTGCTGTGTTATAATGG<br>TCAGGTTTATGATCTGAGCAAAAGCGCAGATCGTAAAGCAACCGCACAGGATGCAGTTAATGCCGAAGGTGAAGCAGATGATATTCGTGAACGTAATCTGCGTA<br>CCACCCGTCTGAATGCAAAACGTGGTGGTGCACATGGTCCGGTTCCGGATGGTTATAAACGTCGTTATGATCCGGATAGCGGTGATCTGGTTGATCAGATTCCG<br>CATCCTGATCGTGCAGGTCTGATTACCGAAATTTTTCGTCGTGCAGCAGCAGCCGAACCGCTGGCAGCAATTTGCCGTGATCTGAATGAACGTGGTGAAACCAC<br>CCATCGTGGTAAAGCATGGCAGCGTCATCATCTGCATGCAATTCTGCGTAATCCTGCATATATTGGTCATCGTCGTCATCTGGGTGTTGATACCGGTAAAGGTA<br>TGTGGGCACCGATTTGTGATGATGAAGATTTTGCAGAAACCTTTCAGGCCGTTCAAGAAATTCTGAGTCTGCCTGGTCGTCAGCTGAGTCCGGGTCCGGAAGCA<br>CAGCATCTGCAGACCGGTATTGCACTGTGTGGTGAACATCCTGATGAACCGCCTCTGCGTAGCGTTACCGTTCGTGGTCGTACCAATTATAACTGTAGCACCCG<br>TTATGATGTTGCCATGCGTGAAGATCGTATGGATGCATTTGTTGAAGAAAGCGTGATTACCTGGCTGGCAAGTGATGAAGCAGTTGCAGCCTTTGAAGATAACA<br>CCGATGATGAACGTACCCGTAAAGCACGTATTCGTCTGAAAGTTCTGGAAGAACAGCTGGAAGCAGCACAGAAACAGGCACGTACCCTGCGTCCGGATGGCATG<br>GGTATGCTGCTGAGCATTGATAGCCTGGCAGGTCTGGAAGCCGAACTGACACCGCAGATTGATAAAGCCCGTCAAGAAAGCCGTAGCCTGCATGTTCCGGCACT<br>GCTGCGTGATCTGCTGGGTAAACCGCGTGCAGATGTGGATCGTGCATGGAATGAAGCACTGACCCTGCCGCAGCGTCGCATGATTCTGCGCATGGTTGTTACCA<br>TTCGTCTGTTTAAAGCAGGTAGCCGTGGTGTTCGTGCAATTGAACCGGGTCGTATTACCCTGAGCTATGTTGGCGAACCGGGTTTTAAACCGGTTGGTGGTAAT<br>CGTGCAAAACAGTAATCACACAGGAAAGTACTAGATGCCTGGTATGACCACCGAAACCGGTCCGGATCCTGCAGGTCTGATTGACCTGTTTTGTCGTAAAAGCA<br>AAGCAGTTAAAAGCCGTGCAAATGGTGCAGGTCAGCGTCGTAAACAAGAAATTAGCATTGCAGCACAAGAAACCCTGGGTCGTAAAGTTGCAGCACTGCTGGGT<br>ATGCAGGTTCGTCATGTTTGGAAAGAAGTTGGTAGCGCAAGCCGTTTTCGTAAAGGTAAAGCACGTGATGATCAGAGCAAAGCACTGAAAGCCCTGGAAAGCGG<br>TGAAGTTGGTGCACTGTGGTGTTATCGTCTGGATCGTTGGGATCGTGGTGGTGCCGGTGCAATTCTGAAAATTATCGAACCGGAAGATGGTATGCCTCGTCGTC<br>TGCTGTTTGGTTGGGATGAAGATACCGGTCGTCCGGTTCTGGATAGCACCAATAAACGTGATCGCGGTGAACTGATTCGTCGTGCAGAAGAAGCACGCGAAGAA<br>GCAGAAAAACTGAGCGAACGTGTTCGTGATACCAAAGCACATCAGCGTGAAAATGGTGAATGGGTTAATGCCCGTGCACCGTATGGTCTGCGTGTTGTTCTGGT<br>TACCGTTAGTGATGAAGAGGGTGATGAATATGATGAACGTAAACTGGCAGCAGATGATGAAGATGCGGGTGGTCCTGATGGTCTGACCAAAGCAGAAGCAGCCC<br>GTCTGGTTTTTACCCTGCCGGTTACCGATCGTCTGAGCTATGCAGGCACCGCACATGCAATGAATACCCGTGAAATTCCGAGCCCGACCGGTGGTCCGTGGATT<br>GCAGTTACCGTGCGTGATATGATTCAGAATCCGGCATATGCGGGTTGGCAGACCACAGGTCGTCAGGATGGTAAACAGCGTCGTCTGACCTTTTATAACGGTGA<br>AGGTAAACGTGTTAGCGTTATGCATGGTCCTCCGCTGGTGACCGATGAAGAACAAGAAGCCGCAAAAGCAGCCGTTAAAGGTGAAGATGGTGTTGGTGTTCCGC<br>TGGATGGTAGCGATCATGATACCCGTCGCAAACATCTGCTGAGCGGTCGTATGCGTTGTCCGGGTTGTGGTGGTAGCTGTAGCTATAGCGGTAATGGTTATCGT<br>TGTTGGCGTAGCAGTGTGAAAGGTGGTTGTCCGGCACCGACCTATGTTGCACGTAAAAGCGTTGAAGAATATGTTGCATTTCGTTGGGCAGCAAAATTAGCAGC<br>AAGCGAACCGGATGATCCGTTTGTTATTGCAGTTGCAGATCGCTGGGCAGCACTGACCCATCCGCAGGCAAGCGAAGATGAAAAGTATGCAAAAGCCGCAGTTC<br>GTGAAGCCGAAAAAAATCTGGGTCGCCTGCTGCGTGATCGTCAGAATGGTGTTTATGATGGTCCGGCAGAACAGTTTTTTGCCCCTGCATATCAAGAAGCACTG<br>AGCACCCTGCAGGCAGCCAAAGATGCAGTTAGCGAAAGCAGCGCAAGCGCAGCAGTTGATGTTAGCTGGATTGTTGATAGCAGCGATTATGAAGAACTGTGGCT<br>GCGTGCAACCCCGACCATGCGTAATGCAATTATTGATACCTGCATCGATGAAATTTGGGTTGCAAAAGGCCAGCGTGGTCGTCCGTTTGATGGTGATGAACGCG<br>TTAAAATCAAATGGGCAGCCCGTACCTAATCACACAGGAAAGTACTAGATGAAAGTGGCCATTTATGTTCGTGTTAGCACCGATGAACAGGCCAAAGAAGGTTT<br>TAGCATTCCGGCACAGCGTGAACGTCTGCGTGCATTTTGTGCAAGCCAGGGTTGGGAAATTGTGCAAGAATATATTGAAGAAGGTTGGAGCGCAAAAGATCTGG<br>ATCGTCCGCAGATGCAGCGTCTGCTGAAAGATATCAAAAAAGGCAACATTGATATTGTGCTGGTGTATCGTCTGGATCGCCTGACCCGTAGCGTTCTGGATCTG<br>TATCTGCTGCTGCAGACCTTTGAAAAATACAATGTGGCATTTCGTAGCGCCACCGAAGTTTATGATACCAGCACCGCAATGGGTCGTCTGTTTATTACCCTGGT<br>TGCAGCACTGGCACAGTGGGAACGTGAAAATCTGGCAGAACGTGTTAAATTTGGTATCGAGCAGATGATCGATGAAGGTAAAAAACCGGGTGGTCATAGCCCGT<br>ATGGTTACAAATTTGATAAAGACTTCAATTGCACCATTATTGAGGAAGAAGCAGACGTTGTTCGTATGATCTATCGCATGTATTGTGATGGTTATGGCTATCGT<br>AGCATTGCAGATCGTCTGAATGAACTGATGGTTAAACCGCGTATTGCCAAAGAATGGAATCATAATAGCGTGCGTGATATCCTGACCAACGATATCTATATTGG<br>CACCTATCGTTGGGGTGATAAAGTTGTTCCGAATAATCATCCGCCTATTATTAGCGAAACCCTGTTCAAAAAAGCCCAGAAAGAAAAAGAAAAACGTGGCGTTG<br>ATCGTAAACGCGTTGGTAAATTTCTGTTTACCGGTCTGCTGCAGTGTGGTAATTGTGGTGGCCATAAAATGCAGGGCCATTTTGATAAACGTGAGCAGAAAACC<br>TATTACCGTTGTACCAAATGTCACCGCATTACCAACGAAAAAAACATTCTGGAACCGCTGCTGGATGAAATTCAGCTGCTGATTACCAGCAAAGAATACTTTAT<br>GAGCAAATTCAGCGACCGCTATGATCAGCAAGAGGTTGTTGATGTTAGCGCACTGACAAAAGAACTGGAAAAAATCAAACGCCAGAAAGAGAAATGGTACGATC<br>TGTATATGGATGATCGTAACCCGATTCCGAAAGAAGAACTGTTTGCCAAAATTAACGAACTGAACAAAAAAGAAGAAGAAATCTATAGCAAGCTGAGCGAAGTG<br>GAAGAAGATAAAGAACCGGTTGAAGAGAAATATAACCGCCTGAGCAAAATGATCGATTTTAAACAGCAGTTTGAGCAGGCCAACGACTTTACCAAAAAAGAGCT<br>GCTGTTCAGCATCTTCGAAAAGATTGTGATTTATCGCGAGAAAGGCAAGCTGAAAAAAATCACCCTGGATTACACCCTGAAATAATCCGGCAATTAAAAAAGCG<br>GCTAACCACGCCGCTTTTTTTACGTCTGCACTCGGTACCAAATTCCAGAAAAGAGGCCTCCCGAAAGGGGGGCCTTTTTTCGTTTTGGTCCGTGGTTAGGCTAG<br>CTACATTTAATGACAATGTGCAGGTTGTACATATTGGTCATTTATTCCGTAACTCGGGTCATAAGGAGTGGCGTATTTTTGTTTGGTTTAATCCAATGCAAGAA<br>CGGAAATGGACTCGATTTACTCATTTGCCTTTATTAAGTCGAGCTAAGGTGGTTAACAGTACAACAAAGCAAATAAATAAGGCGGATCGTGTGATTGAGTTTGA<br>AGCATCGGATCTTCAACGAGCCAAAATAATCGGGGGATCCTCTAGAGTCGACCTGCAGGCATGCAAGCTTGGCACTGGCCGTCGTTTTACAACGTCGTGACTGG<br>GAAAACCCTGGCGTTACCCAACTTAATCGCCTTGCAGCACATCCCCCTTTCGCCAGCTGGCGTAATAGCGAAGAGGCCCGCACCGATCGCCCTTCCCAACAGTT<br>GCGCAGCCTGAATGGCGAATGGCGCCTGATGCGGTATTTTCTCCTTACGCATCTGTGCGGTATTTCACACCGCATATGGTGCACTCTCAGTACAATCTGCTCTG<br>ATGCCGCATAGTTAAGCCAGCCCCGACACCCGCCAACACCCGCTGACGCGCCCTGACGGGCTTGTCTGCTCCCGGCATCCGCTTACAGACAAGCTGTGACCGTC<br>TCCGGGAGCTGCATGTGTCAGAGGTTTTCACCGTCATCACCGAAACGCGCGAGACGAAAGGGCCTCGTGATACGCCTATTTTTATAGGTTAATGTCATGATAAT<br>AATGGTTTCTTAGACGTCAGGTGGCACTTTTCGGGGAAATGTGCGCGGAACCCCTATTTGTTTATTTTTCTAAATACATTCAAATATGTATCCGCTCATGAGAC

	AATAACCCTGATAAATGCTTCAATAATATTGAAAAAGGAAGAGTATGAGTATTCAACATTTCCGTGTCGCCCTTATTCCCTTTTTTGCGGCATTTTGCCTTCCT<br>GTTTTTGCTCACCCAGAAACGCTGGTGAAAGTAAAAGATGCTGAAGATCAGTTGGGTGCACGAGTGGGTTACATCGAACTGGATCTCAACAGCGGTAAGATCCT<br>TGAGAGTTTTCGCCCCGAAGAACGTTTTCCAATGATGAGCACTTTTAAAGTTCTGCTATGTGGCGCGGTATTATCCCGTATTGACGCCGGGCAAGAGCAACTCG<br>GTCGCCGCATACACTATTCTCAGAATGACTTGGTTGAGTACTCACCAGTCACAGAAAAGCATCTTACGGATGGCATGACAGTAAGAGAATTATGCAGTGCTGCC<br>ATAACCATGAGTGATAACACTGCGGCCAACTTACTTCTGACAACGATCGGAGGACCGAAGGAGCTAACCGCTTTTTTGCACAACATGGGGGATCATGTAACTCG<br>CCTTGATCGTTGGGAACCGGAGCTGAATGAAGCCATACCAAACGACGAGCGTGACACCACGATGCCTGTAGCAATGGCAACAACGTTGCGCAAACTATTAACTG<br>GCGAACTACTTACTCTAGCTTCCCGGCAACAATTAATAGACTGGATGGAGGCGGATAAAGTTGCAGGACCACTTCTGCGCTCGGCCCTTCCGGCTGGCTGGTTT<br>ATTGCTGATAAATCTGGAGCCGGTGAGCGTGGGTCTCGCGGTATCATTGCAGCACTGGGGCCAGATGGTAAGCCCTCCCGTATCGTAGTTATCTACACGACGGG<br>GAGTCAGGCAACTATGGATGAACGAAATAGACAGATCGCTGAGATAGGTGCCTCACTGATTAAGCATTGGTAACTGTCAGACCAAGTTTACTCATATATACTTT<br>AGATTGATTTAAAACTTCATTTTTAATTTAAAAGGATCTAGGTGAAGATCCTTTTTGATAATCTCATGACCAAAATCCCTTAACGTGAGTTTTCGTTCCACTGA<br>GCGTCAGACCCCGTAGAAAAGATCAAAGGATCTTCTTGAGATCCTTTTTTTCTGCGCGTAATCTGCTGCTTGCAAACAAAAAAACCACCGCTACCAGCGGTGGT<br>TTGTTTGCCGGATCAAGAGCTACCAACTCTTTTTCCGAAGGTAACTGGCTTCAGCAGAGCGCAGATACCAAATACTGTCCTTCTAGTGTAGCCGTAGTTAGGCC<br>ACCACTTCAAGAACTCTGTAGCACCGCCTACATACCTCGCTCTGCTAATCCTGTTACCAG

[A] Annotations: **Promoters**; Ribozymes; CDS; Terminators; RBSs; Antibiotic resistance gene; origin of rep

[B] Reported in (Meyer *et al.*, 2019); [C] Reported in (Park *et al*, 2020)

References

Chen Y, Kim JK, Hirning AJ, Josic K, Bennett MR (2015) Emergent genetic oscillations in a synthetic microbial consortium. *Science* 349: 986-989

Lithgow JK, Wilkinson A, Hardman A, Rodelas B, Wisniewski-Dye F, Williams P, Downie JA (2000) The regulatory locus cinRI in Rhizobium leguminosarum controls a network of quorum-sensing loci. *Mol Microbiol* 37: 81-97

Meyer AJ, Segall-Shapiro TH, Glassey E, Zhang J, Voigt CA (2019) Escherichia coli "Marionette" strains with 12 highly optimized small-molecule sensors. *Nat Chem Biol* 15: 196-204

Moon TS, Lou C, Tamsir A, Stanton BC, Voigt CA (2012) Genetic programs constructed from layered logic gates in single cells. *Nature* 491: 249-253

Park Y, Espah Borujeni A, Gorochowski TE, Shin J, Voigt CA (2020) Precision design of stable genetic circuits carried in highly-insulated E. coli genomic landing pads. *Mol Syst Biol* 16: e9584

Schaefer AL, Greenberg EP, Oliver CM, Oda Y, Huang JJ, Bittan-Banin G, Peres CM, Schmidt S, Juhaszova K, Sufrin JR *et al* (2008) A new class of homoserine lactone quorum-sensing signals. *Nature* 454: 595-599

Tamsir A, Tabor JJ, Voigt CA (2011) Robust multicellular computing using genetically encoded NOR gates and chemical 'wires'. *Nature* 469: 212-215
