# Supplementary material for: Characterizing chemical signaling between engineered “microbial sentinels” in porous microplates
Source: Mol Syst Biol. 2022 Mar 22;18(3):e10785. doi: 10.15252/msb.202110785 (PMC8938921; doi:10.15252/msb.202110785)
Supplement: Supplementary file 3 [file MSB-18-e10785-s001.pdf]

molecular systems biology

Characterizing chemical signaling between engineered "microbial sentinels" in porous microplates

Christopher Vaiana, Hyungseok Kim, Jonathan Cottet, Keiko Oai, Zhifei Ge, Kameron Conforti, Andrew King, Adam Meyer, Haorong Chen, Christopher Voigt, and Cullen Buie
DOI: 10.15252/msb.202110785

Corresponding author(s): Christopher Voigt (cavoigt@gmail.com), Cullen Buie (crb@mit.edu)

Review Timeline:

Submission Date:	1st Nov 21
Editorial Decision:	30th Nov 21
Revision Received:	29th Dec 21
Editorial Decision:	10th Jan 22
Revision Received:	24th Jan 22
Accepted:	25th Jan 22

Editor: Maria Polychronidou

Transaction Report:

(Note: With the exception of the correction of typographical or spelling errors that could be a source of ambiguity, letters and reports are not edited. Depending on transfer agreements, referee reports obtained elsewhere may or may not be included in this compilation. Referee reports are anonymous unless the Referee chooses to sign their reports.)

1st Editorial Decision **30th Nov 2021**

Thank you again for submitting your work to Molecular Systems Biology. We have now heard back from the three reviewers who agreed to evaluate your study. As you will see below, the reviewers acknowledge that the study is interesting. They raise however a series of concerns, which we would ask you to address in a revision.

I think that the reviewers' recommendations are clear and therefore there is no need to repeat the points listed below. All issues raised by the reviewers need to be satisfactorily addressed. Please contact me in case you would like to discuss in further detail any of the issues raised.

On a more editorial level, we would ask you to address the following points:

REFEREE REPORTS

Reviewer #1:

This manuscript provides a clear description of a new materials platform technology for studying cell-cell signaling through porous matrices and they describe the development of an expanded set of transcriptional regulators that can be used orthogonally from one another. The authors present a nice set of proof of concept experiments for evaluating the movement of a variety of molecules through this material, ranging from chemical dyes to cell-cell signals to synthetic chemical inducers to antibiotics. These experiments show how chemical concentrations vary with distance and time, and a quantitative model is used to fit an analyze the data that considers effective diffusivity and adsorption isotherm models. In general, the manuscript is well written, the data is clearly presented, and the conclusions are supported by the data. The technologies presented will be of interest to the readership of the journal.

Additional comments:

Long chain AHL data. The modeling seem to only consider diffusivity and adsorption, and a supplemental figure is presented suggesting that these parameters are largely responsible for the observed trends. Long chain AHLs have been shown to exhibit pH-dependent hydrolysis (DOI, 10.1128/IAI.70.10.5635-5646.2002) which has been shown to be modified by porous environmental materials (DOI, 10.1021/acsomega.6b00085). These pH dependent kinetics are relatively fast on the time scale of the experiments presented, and it would be good to note that some of the data points shown in Figure S9 may be influenced by such processes.

Abstract: "quorums" is not clear; suggest changing to autoinducers or quorum signal

Reviewer #2:

Vaiana et al. present a very interesting study on the development of living materials composed of a bespoke porous microplate with hydrogel (methacrylate) barriers intercalated between wells that manage transport phenomenon, which contain engineered E. coli chassis cells. The authors developed an engineered E. coli chassis cell with biosensing capabilities to IPTG, anhydrotetracycline, L-arabinose, and four quorums (luxR, cinR, lasR, and rpaR) - fashioned after the previously reported "Marionette Strain". Transport characteristics of dyes, antibiotics, inducers, and quorums between wells were assessed. The resulting "sentinel" strain's response to inducer diffusion through the wells was quantified up to 14 mm, and quorum and antibacterial signaling were measured over 16 h. The author's assess how adsorption impacts the transport of each molecule differently, and deduce an adsorption coefficient for each inducer with respect to the methacrylate scaffold using a linear adsorption model.

In general, I enjoyed reading this manuscript and found the results to be of high-quality. At times, I was somewhat puzzled by seemingly disconnected objectives and the purpose of certain aspects of the work - however I believe this could be remedied by additional discussion rather than by more experiments. All things considered, this work should be of significant interest to the MSB community and I am supportive of publication after some revision.

Comment: To test the permeability of the microplate walls, the authors measured the effective diffusion of the visible dyes cotton blue and rhodamine B from the center well. Why were these two dyes selected (i.e., what are the dyes physical and chemical properties, and justification for use)?

Comment: Figure 1d can the authors include error bars. As indicated, each data point represents a single device and each condition was measured three times.

Comment: The authors put considerable effort into engineering a sensor strain that responds orthogonally to 7 small molecules. Why? Were the authors interested in facilitating the detection of a diverse collection of small molecules - i.e., with different physicochemical properties? Will this chassis be used for a specific purpose in future applications?

Comment: Please consider replacing "Henry's law" with "Henry's adsorption isotherm". Also, what was the purpose of the simulations for transport through the microplate of the seven inducers that activate the sensor strain (Fig. 3a)? Will this aid in the design of new materials?

Comment: The authors surmise that the predominant factor determining the effective diffusion distance of each molecule is the propensity of the hydrogel matrix to adsorb that inducer, and this adsorption is related to the solubility of the molecule. Can the

authors propose what alternate materials would potentially mitigate adsorption?

Reviewer #3:

The manuscript by Vaiana et. al. shows the design and construction of a porous microplate with and its use as living material. In particular, they apply thy demonstrate that it is possible to use their platform to study to detect several small molecules orthogonally, cell to cell communication in the form of quorum sensing, the detection and performance of antibiotics and antimicrobial activity of bacteria.
The manuscript is written in a clear and easy to understand style, the methodology properly described, and the results support the conclusions drawn from them. However, the authors should address several points before the manuscript is acceptable for publication in Molecular System Biology:

Major points:

The term absorption is used several times probably referring to adsorption, for example in line 244: 'adsorption coefficients ranging from 0 mol m-3 (no absorption)' Authors should define the interaction between the diffusing molecules and the porous microplate and use the right term.

Interactions between the solute and the HEMA-EDMA microplate walls: the authors state that the diffusion of rhodamine B is impaired by its adsorption in the polymer. However, the Fig 1 g. and h. show the walls stained to a higher extent when using cotton blue than with rhodamine B. Authors should consider other interactions with the polymer besides adsorption such as electrostatic attraction repulsion to explain the differences in the polymer-dye interactions. Besides that, as it seems from the graphs on these figures, the concentration of dye added in the central well is different for both dyes, being higher that for cotton blue. Authors should justify that difference or use the same concentration for both of them.

Authors should justify why they have used a simulation of the diffusion and adsorption of the inducers through the microplate instead a direct measurement of the concentration of the inducers in each well. They should also consider to experimentally validate the profiles of their model with the direct measurement of their concentration besides the indirect method of predicting the RPU profile and comparing it with the observed RPU of each output strain. This might be a more direct way to estimate the value of KL.

Authors should explain in more detail the reason behind the selection of the concentration of each inducer in the mix introduced in the center well for the experiment in Figure 3. Is it based in the detection limits found in the dose-response curves? Authors should discuss about the importance, or not, of the concentration of the inducer in the center well on its diffusion and adsorption in the walls of the microplate.

Minor points:

- In the abstract use signal/signaling molecules instead quorums? Review this for the entire paper, for example in line 159 use 'signaling molecules sensor' instead of 'quorum sensors'
- Lines 58 and 62, use the same nomenclature, either um or microns
- Line 62, write Escherichia Coli since it is the first time mentioned in the text. The same applies for all the other species mentioned in the manuscript
- Figure 1 h: re-size the y axis of the graph
- Figure 4 d: check the labels of the y axes, the 0.1 should be 1?
- Figure 4 e and experiment with the strain producing Microcin A: in the text authors mention that they "observed a greater than 50 % cell death at a distance of one well away from the center microcin producing culture", however in the graph it appears that the inhibition was around 90% for all the cases. Authors should check and correct this discrepancy.
- Line 446: specify the concentration of the dye.
- Please indicate the mutations found on the optimized activators.
- In table 2, the partition coefficient is named as KL, while in equation 2 us just K. Please unify.

Reviewer #1:

This manuscript provides a clear description of a new materials platform technology for studying cell-cell signaling through porous matrices and they describe the development of an expanded set of transcriptional regulators that can be used orthogonally from one another. The authors present a nice set of proof of concept experiments for evaluating the movement of a variety of molecules through this material, ranging from chemical dyes to cell-cell signals to synthetic chemical inducers to antibiotics. These experiments show how chemical concentrations vary with distance and time, and a quantitative model is used to fit an analyze the data that considers effective diffusivity and adsorption isotherm models. In general, the manuscript is well written, the data is clearly presented, and the conclusions are supported by the data. The technologies presented will be of interest to the readership of the journal.

Additional comments:

Long chain AHL data. The modeling seems to only consider diffusivity and adsorption, and a supplemental figure is presented suggesting that these parameters are largely responsible for the observed trends. Long chain AHLs have been shown to exhibit pH-dependent hydrolysis (DOI, 10.1128/IAI.70.10.5635-5646.2002) which has been shown to be modified by porous environmental materials (DOI, 10.1021/acsomega.6b00085). These pH dependent kinetics are relatively fast on the time scale of the experiments presented, and it would be good to note that some of the data points shown in Figure S9 may be influenced by such processes.

We agree with the reviewer’s point regarding the effects of pH-dependent kinetics on the half-life of homoserine lactones due to a ring-opening hydrolysis transformation, or “lactonolysis.” We have added the following paragraph to discuss the potential impact of lactonolysis on our observations at Line 303:

“It is important to note that other environmental factors may influence the bioavailability of signaling molecules in porous materials. For instance, homoserine lactones will undergo lactonolysis, a ring opening transformation that is dependent on environmental factors like pH and temperature, and that is stabilized to different degrees based on acyl chain length (Yates et al, 2002). In liquid culture, the half-life of OC6 can decreases from 32 h in LB media at pH = 6.0, to ~ 9 h in M9 media at pH = 7 (Politi et al, 2014). The implications of lactonolysis is apparent on environmentally relevant quorum signaling, such as the changes in homoserine lactone accumulation in biocharred soils (Gao et al, 2016b). Given that lactonolysis kinetics occur on timescales relevant to our studies, these processes may influence the observed signaling distances presented in Supplementary Fig. 9 and Fig. 4A, in addition to solubility and hydrogel adsorption.”

Abstract: "quorums" is not clear; suggest changing to autoinducers or quorum signal

We agree, and “quorums” has been replaced by “quorum signals” in the abstract.

Reviewer #2:

Vaiana et al. present a very interesting study on the development of living materials composed of a bespoke porous microplate with hydrogel (methacrylate) barriers intercalated between wells that manage transport phenomenon, which contain engineered E. coli chassis cells. The authors developed an engineered E. coli chassis cell with biosensing capabilities to IPTG, anhydrotetracycline, L-arabinose, and four quorums (luxR, cinR, lasR, and rpaR) - fashioned after the previously reported "Marionette Strain". Transport characteristics of dyes, antibiotics, inducers, and quorums between wells were assessed. The resulting "sentinel" strain's response to inducer diffusion through the wells was quantified up to 14 mm, and quorum and antibacterial signaling were measured over 16 h. The author's assess how adsorption impacts the transport of each molecule differently, and deduce an adsorption coefficient for each inducer with respect to the methacrylate scaffold using a linear adsorption model.

In general, I enjoyed reading this manuscript and found the results to be of high-quality. At times, I was somewhat puzzled by seemingly disconnected objectives and the purpose of certain aspects of the work - however I believe this could be remedied by additional discussion rather than by more experiments. All things considered, this work should be of significant interest to the MSB community and I am supportive of publication after some revision.

Comment: To test the permeability of the microplate walls, the authors measured the effective diffusion of the visible dyes cotton blue and rhodamine B from the center well. Why were these two dyes selected (i.e., what are the dyes physical and chemical properties, and justification for use)?

These dyes were selected because they have similar Molecular Weights (MW) but different water solubilities. The manuscript text has been updated to include this clarification at line 162.

Comment: Figure 1d can the authors include error bars. As indicated, each data point represents a single device and each condition was measured three times.

We refer to a specific decanol / cyclohexanol ratio as a “condition.” There are three devices fabricated per condition, and one measurement was taken for each of those devices. All collected data are plotted in Figure 1d, and therefore do not warrant error bars. This has been clarified in the Figure 1d legend.

Comment: The authors put considerable effort into engineering a sensor strain that responds orthogonally to 7 small molecules. Why? Were the authors interested in facilitating the detection of a diverse collection of small molecules - i.e., with different physicochemical properties? Will this chassis be used for a specific purpose in future applications?

Our original intent was to study layers of multi-input induction and sequential auto-induced quorum signaling cascades using the microplate, but the effects of signal adsorption were too strong to achieve this scope. We do hope this strain can be applied toward these studies in the future.

Comment: Please consider replacing "Henry's law" with "Henry's adsorption isotherm". Also, what was the purpose of the simulations for transport through the microplate of the

seven inducers that activate the sensor strain (Fig. 3a)? Will this aid in the design of new materials?

We agree with the change from "Henry's law" to "Henry's adsorption isotherm" and have made this change in the manuscript at line 238. The purpose of the simulations was to assign a quantitative parameter – in this case adsorption coefficient – to explain the transport of different biological signaling molecules. This quantification, we hope, will in fact aid in the design of new materials that may rely on biological signaling cascades, so that signaling distances can be better predicted.

Comment: The authors surmise that the predominant factor determining the effective diffusion distance of each molecule is the propensity of the hydrogel matrix to adsorb that inducer, and this adsorption is related to the solubility of the molecule. Can the authors propose what alternate materials would potentially mitigate adsorption?

We have discussed alternative hydrogel materials with varying properties in the introduction. In addition, we have included the following statement in the discussion referencing some work done in alginate-acrylamides polymers that has shown less absorption of biological inducers at line 376:

"Some applications may warrant the use of porous materials that allow inducers to pass unhindered, such as alginate, acrylamide, and agarose (Liu et al., 2017; Liu et al., 2018)."

Reviewer #3:

The manuscript by Vaiana et. al. shows the design and construction of a porous microplate with and its use as living material. In particular, they apply thy demonstrate that it is possible to use their platform to study to detect several small molecules orthogonally, cell to cell communication in the form of quorum sensing, the detection and performance of antibiotics and antimicrobial activity of bacteria.
The manuscript is written in a clear and easy to understand style, the methodology properly described, and the results support the conclusions drawn from them. However, the authors should address several points before the manuscript is acceptable for publication in Molecular System Biology:

Major points:

The term absorption is used several times probably referring to adsorption, for example in line 244: 'adsorption coefficients ranging from 0 mol m-3 (no absorption)' Authors should define the interaction between the diffusing molecules and the porous microplate and use the right term.

We acknowledge that the proper term is "adsorption" and we have corrected the text accordingly.

Interactions between the solute and the HEMA-EDMA microplate walls: the authors state that the diffusion of rhodamine B is impaired by its adsorption in the polymer. However, the Fig 1 g. and h. show the walls stained to a higher extent when using cotton blue than with rhodamine B. Authors should consider other interactions with the polymer besides adsorption such as electrostatic attraction repulsion to explain the differences in the polymer-dye interactions. Besides that, as it seems from the graphs on these figures, the concentration of dye added in the central well is different for both dyes, being higher that for cotton blue. Authors should justify that difference or use the same concentration for both of them.

Our initial experiment varied the starting concentration of dye slightly; 12.5 mM for methyl blue and 15.7 mM for rhodamine B. However, we do agree that there was no justification for this difference, therefore we repeated the methyl blue diffusion experiment with a starting concentration of 15.7 mM, and we have added the new data to Figure 1 with the axes of Fig 1G and H normalized. We can now directly compare the results of the two dyes. The fact that the center well was nearly depleted of rhodamine suggests that the adsorption of rhodamine to the hydrogel was significantly more than that of methyl blue.

We agree that electrostatic interactions play a role in molecule transport and we have added the following lines and additional references in the discussion to acknowledge this point at line 372:

"Environmental factors like pH and temperature play a role in dictating the solute interaction with the matrix, which could include attractive or repulsive electrostatics, hydrogen bonding, and hydrophobic effects (Hernandez-Martínez et al, 2018; Kudaibergenov et al, 2016). Our simplified model summarizes these forces as a single "adsorption coefficient," acknowledging that the entirety of these interactions has not been accounted for."

Authors should justify why they have used a simulation of the diffusion and adsorption of the inducers through the microplate instead a direct measurement of the concentration of

the inducers in each well. They should also consider to experimentally validate the profiles of their model with the direct measurement of their concentration besides the indirect method of predicting the RPU profile and comparing it with the observed RPU of each output strain. This might be a more direct way to estimate the value of KL.

While we agree that a direct measurement of the concentration of each molecule would be a direct way to calculate K_H, through the dose-response curves we have demonstrated that we can use the engineered sentinel strain as a tool to measure trace amounts of each inducer molecule in the small volume within each well. It would be technically challenging to accurately quantify these amounts by other means (mass spectrometry for example). Furthermore, it is ultimately the biological response that we are interested in quantifying, as it serves as a proof of concept for the design of a "living material" with chemical sensing capabilities, and therefore we chose to rely on this response to define the adsorption parameters of the model.

Authors should explain in more detail the reason behind the selection of the concentration of each inducer in the mix introduced in the center well for the experiment in Figure 3. Is it based in the detection limits found in the dose-response curves? Authors should discuss about the importance, or not, of the concentration of the inducer in the center well on its diffusion and adsorption in the walls of the microplate.

The following paragraph has been added to the manuscript in order to clarify the choice of inducer concentration at line 258:

"We concentrated the inducers to 20 times the maximum concentration as determined by the dose response curves. These concentrations were chosen in an effort to ensure a detectable signal would be achieved several wells away from the center once the inducer diffuses away, but were within the water solubility limits of the inducer molecules."

Minor points:

• In the abstract use signal/signaling molecules instead quorums? Review this for the entire paper, for example in line 159 use 'signaling molecules sensor' instead of 'quorum sensors'
We changed quorums to "quorum signals" in the abstract in response to this comment as well as comments from Reviewer #1.

• Lines 58 and 62, use the same nomenclature, either um or microns
We corrected this by using "μm" instead of micron throughout the manuscript.

• Line 62, write Escherichia Coli since it is the first time mentioned in the text. The same applies for all the other species mentioned in the manuscript
We have corrected this throughout the manuscript.

• Figure 1 h: re-size the y axis of the graph
We have re-sized the y axis in Figure 1H.

• Figure 4 d: check the labels of the y axes, the 0.1 should be 1?
We have corrected the y-axis labels in Figure 4d.

• Figure 4 e and experiment with the strain producing Microcin A: in the text authors mention that they "observed a greater than 50 % cell death at a distance of one well

away from the center microcin producing culture", however in the graph it appears that the inhibition was around 90% for all the cases. Authors should check and correct this discrepancy.

We observe ~ 90 % inhibition due to diffusion of the antibiotics that are depicted in Figure 4d; however, the inhibition resulting from the Microcin producer is correctly annotated as greater than ~ 50% as indicated in Figure 4e.

• Line 446: specify the concentration of the dye.
We have updated the text to indicate the dye concentration.

• Please indicate the mutations found on the optimized activators.
We have added the specific sequences of the original and optimized promoters and activators in Appendix Table S1 and S2.

• In table 2, the partition coefficient is named as KL, while in equation 2 us just K. Please unify.
We actually chose to label the partition coefficient K_H in both locations; to delineate from K used in the Hill coefficients in Table 1, and because it is derived from Henry's Adsorption Isotherm (as opposed to the Langmuir Isotherm which often uses a constant annotated K_L).

1st Revision - Editorial Decision 10th Jan 2022

Thank you for sending us your revised manuscript. We have now evaluated your revised study and we think that the performed revisions have satisfactorily addressed the issues raised by the reviewers. As such I am glad to inform your that we can soon proceed with formally accepting the study for publication. Before we can proceed with the formal acceptance, we would also ask you to address some minor editorial issues listed below.

2nd Authors' Response to Reviewers 24th Jan 2022

The authors have made all requested editorial changes.

Accepted **25th Jan 2022**

Thank you again for sending us your revised manuscript. We are now satisfied with the modifications made and I am pleased to inform you that your paper has been accepted for publication.

EMBO PRESS

YOU MUST COMPLETE ALL CELLS WITH A PINK BACKGROUND ↓

PLEASE NOTE THAT THIS CHECKLIST WILL BE PUBLISHED ALONGSIDE YOUR PAPER

Corresponding Author Name: Cullen R Buie
Molecular Systems Biology
Manuscript Number: MSB-2021-10785

Reporting Checklist For Life Sciences Articles (Rev. June 2017)

This checklist is used to ensure good reporting standards and to improve the reproducibility of published results. These guidelines are consistent with the Principles and Guidelines for Reporting Preclinical Research issued by the NIH in 2014. Please follow the journal's authorship guidelines in preparing your manuscript.

A- Figures
1. Data
The data shown in figures should satisfy the following conditions:

- the data were obtained and processed according to the field's best practice and are presented to reflect the results of the experiments in an accurate and unbiased manner.
- figure panels include only data points, measurements or observations that can be compared to each other in a scientifically meaningful way.
- graphs include clearly labeled error bars for independent experiments and sample sizes. Unless justified, error bars should not be shown for technical replicates.
- if n< 5, the individual data points from each experiment should be plotted and any statistical test employed should be justified
- Source Data should be included to report the data underlying graphs. Please follow the guidelines set out in the author ship guidelines on Data Presentation.

2. Captions

Each figure caption should contain the following information, for each panel where they are relevant:

- a specification of the experimental system investigated (eg cell line, species name).
- the assay(s) and method(s) used to carry out the reported observations and measurements
- an explicit mention of the biological and chemical entity(ies) that are being measured.
- an explicit mention of the biological and chemical entity(ies) that are altered/varied/perturbed in a controlled manner.

- the exact sample size (n) for each experimental group/condition, given as a number, not a range;
- a description of the sample collection allowing the reader to understand whether the samples represent technical or biological replicates (including how many animals, litters, cultures, etc.).
- a statement of how many times the experiment shown was independently replicated in the laboratory.
- definitions of statistical methods and measures:
  - common tests, such as t-test (please specify whether paired vs. unpaired), simple χ2 tests, Wilcoxon and Mann-Whitney tests, can be unambiguously identified by name only, but more complex techniques should be described in the methods section;
  - are tests one-sided or two-sided?
  - are there adjustments for multiple comparisons?
  - exact statistical test results, e.g., P values = x but not P values < x;
  - definition of 'center values' as median or average;
  - definition of error bars as s.d. or s.e.m.

Any descriptions too long for the figure legend should be included in the methods section and/or with the source data.

In the pink boxes below, please ensure that the answers to the following questions are reported in the manuscript itself. Every question should be answered. If the question is not relevant to your research, please write NA (non applicable). We encourage you to include a specific subsection in the methods section for statistics, reagents, animal models and human subjects.

USEFUL LINKS FOR COMPLETING THIS FORM

http://www.antibodypedia.com
http://1degreebio.org
http://www.equator-network.org/reporting-guidelines/improving-bioscience-research-repor
http://grants.nih.gov/grants/olaw/olaw.htm
http://www.mrc.ac.uk/Ourresearch/Ethicsresearchguidance/Useofanimals/index.htm
http://ClinicalTrials.gov
http://www.consort-statement.org
http://www.consort-statement.org/checklists/view/32-consort/66-title
http://www.equator-network.org/reporting-guidelines/reporting-recommendations-for-tumo
http://datadryad.org
http://figshare.com
http://www.ncbi.nlm.nih.gov/gap
http://www.ebi.ac.uk/ega
http://biomodels.net/
http://biomodels.net/miriam/
http://jjj.biochem.sun.ac.za
https://osp.od.nih.gov/biosafety-biosecurity-and-emerging-biotechnology/
http://www.selectagents.gov/

B- Statistics and general methods

Please fill out these boxes ↓ (Do not worry if you cannot see all your text once you press return)

1.a. How was the sample size chosen to ensure adequate power to detect a pre-specified effect size?	At least Data from more than 20,000 E. coli cells were gathered and analyzed by Flow Cytometry to avoid statistical bias. Data were collected on three different devices for each experiment to avoid material fabrication bias.
1.b. For animal studies, include a statement about sample size estimate even if no statistical methods were used.	n/a
2. Describe inclusion/exclusion criteria if samples or animals were excluded from the analysis. Were the criteria pre-established?	n/a
3. Were any steps taken to minimize the effects of subjective bias when allocating animals/samples to treatment (e.g. randomization procedure)? If yes, please describe.	n/a
For animal studies, include a statement about randomization even if no randomization was used.	n/a
4.a. Were any steps taken to minimize the effects of subjective bias during group allocation or/and when assessing results (e.g. blinding of the investigator)? If yes please describe.	no
4.b. For animal studies, include a statement about blinding even if no blinding was done	n/a
5. For every figure, are statistical tests justified as appropriate?	Yes
Do the data meet the assumptions of the tests (e.g., normal distribution)? Describe any methods used to assess it.	n/a
Is there an estimate of variation within each group of data?	Variation in promoter activity of the conditions tested are visualized in the representative cytometry trases and available to view in the appendix.

Is the variance similar between the groups that are being statistically compared?	yes

C- Reagents

6. To show that antibodies were profiled for use in the system under study (assay and species), provide a citation, catalog number and/or clone number, supplementary information or reference to an antibody validation profile. e.g., Antibodypedia (see link list at top right), 1DegreeBio (see link list at top right).	n/a
7. Identify the source of cell lines and report if they were recently authenticated (e.g., by STR profiling) and tested for mycoplasma contamination.	n/a

* for all hyperlinks, please see the table at the top right of the document

D- Animal Models

8. Report species, strain, gender, age of animals and genetic modification status where applicable. Please detail housing and husbandry conditions and the source of animals.	n/a
9. For experiments involving live vertebrates, include a statement of compliance with ethical regulations and identify the committee(s) approving the experiments.	n/a
10. We recommend consulting the ARRIVE guidelines (see link list at top right) (PLoS Biol. 8(6), e1000412, 2010) to ensure that other relevant aspects of animal studies are adequately reported. See author guidelines, under 'Reporting Guidelines'. See also: NIH (see link list at top right) and MRC (see link list at top right) recommendations. Please confirm compliance.	n/a

E- Human Subjects

11. Identify the committee(s) approving the study protocol.	n/a
12. Include a statement confirming that informed consent was obtained from all subjects and that the experiments conformed to the principles set out in the WMA Declaration of Helsinki and the Department of Health and Human Services Belmont Report.	n/a
13. For publication of patient photos, include a statement confirming that consent to publish was obtained.	n/a
14. Report any restrictions on the availability (and/or on the use) of human data or samples.	n/a
15. Report the clinical trial registration number (at ClinicalTrials.gov or equivalent), where applicable.	n/a
16. For phase II and III randomized controlled trials, please refer to the CONSORT flow diagram (see link list at top right) and submit the CONSORT checklist (see link list at top right) with your submission. See author guidelines, under 'Reporting Guidelines'. Please confirm you have submitted this list.	n/a
17. For tumor marker prognostic studies, we recommend that you follow the REMARK reporting guidelines (see link list at top right). See author guidelines, under 'Reporting Guidelines'. Please confirm you have followed these guidelines.	n/a

F- Data Accessibility

18: Provide a "Data Availability" section at the end of the Materials & Methods, listing the accession codes for data generated in this study and deposited in a public database (e.g. RNA-Seq data: Gene Expression Omnibus GSE39462, Proteomics data: PRIDE PXD000208 etc.) Please refer to our author guidelines for 'Data Deposition'.<br><br>Data deposition in a public repository is mandatory for:<br>a. Protein, DNA and RNA sequences<br>b. Macromolecular structures<br>c. Crystallographic data for small molecules<br>d. Functional genomics data<br>e. Proteomics and molecular interactions	yes
19. Deposition is strongly recommended for any datasets that are central and integral to the study; please consider the journal's data policy. If no structured public repository exists for a given data type, we encourage the provision of datasets in the manuscript as a Supplementary Document (see author guidelines under 'Expanded View' or in unstructured repositories such as Dryad (see link list at top right) or Figshare (see link list at top right).	
20. Access to human clinical and genomic datasets should be provided with as few restrictions as possible while respecting ethical obligations to the patients and relevant medical and legal issues. If practically possible and compatible with the individual consent agreement used in the study, such data should be deposited in one of the major public access-controlled repositories such as dbGAP (see link list at top right) or EGA (see link list at top right).	
21. Computational models that are central and integral to a study should be shared without restrictions and provided in a machine-readable form. The relevant accession numbers or links should be provided. When possible, standardized format (SBML, CellML) should be used instead of scripts (e.g. MATLAB). Authors are strongly encouraged to follow the MIRIAM guidelines (see link list at top right) and deposit their model in a public database such as Biomodels (see link list at top right) or JWS Online (see link list at top right). If computer source code is provided with the paper, it should be deposited in a public repository or included in supplementary information.	

G- Dual use research of concern

22. Could your study fall under dual use research restrictions? Please check biosecurity documents (see link list at top right) and list of select agents and toxins (APHIS/CDC) (see link list at top right). According to our biosecurity guidelines, provide a statement only if it could.	n/a
